# Amyloid Precursor Protein Processing Links Female Urgency Urinary Incontinence with Alzheimer’s Disease: Implications for Treatment

**DOI:** 10.3390/ijms27146156

**Published:** 2026-07-09

**Authors:** Wilke M. Post, Joanna Widomska, Egbert Oosterwijk, Ward De Witte, Dorien M. Tiemessen, Cornelius J. H. M. Klemann, Il Hyun Ruisch, Marieke J. H. Coenen, Dick A. W. Janssen, Frank Martens, Megan U. Carnes, Jesse A. Marks, Grier P. Page, Holly E. Richter, Rufus Cartwright, Vatche A. Minassian, Laurent F. Thomas, Anne H. Skogholt, Signe N. Stafne, Kristian Hveem, Kirsten B. Kluivers, Geert Poelmans

**Affiliations:** 1 Department of Obstetrics and Gynecology, Radboud University Medical Center, 6525 GA Nijmegen, The Netherlands; wilke.post@radboudumc.nl (W.M.P.); kirsten.kluivers@radboudumc.nl (K.B.K.); 2 Donders Institute for Brain, Cognition and Behaviour, Radboud University, 6525 GA Nijmegen, The Netherlands; joanna.widomska@radboudumc.nl (J.W.);; 3 Department of Medical Neuroscience, Radboud University Medical Center, 6525 GA Nijmegen, The Netherlands; 4 Department of Urology, Radboud University Medical Center, 6525 GA Nijmegen, The Netherlands; 5 Department of Clinical Chemistry, ErasmusMC, 3000 CA Rotterdam, The Netherlands; 6 Department of Human Genetics, Radboud University Medical Center, 6525 GA Nijmegen, The Netherlands; 7 Genomics, Bioinformatics, and Translational Research Center, RTI International, Research Triangle Park, Durham, NC 27713, USA; 8 RTI International, Research Triangle Park, Atlanta, GA 30341, USA; 9 Department of Obstetrics and Gynecology, University of Alabama at Birmingham, Birmingham, AL 35233, USA; 10 Department of Gynaecology, Chelsea and Westminster NHS Foundation Trust, London SW10 9NH, UK; 11 Department of Epidemiology and Biostatistics, Imperial College London, London W2 1PG, UK; 12 Department of Obstetrics and Gynecology, Brigham and Women’s Hospital, Boston, MA 02115, USA; 13 HUNT Center for Molecular and Clinical Epidemiology, Department of Public Health and Nursing, Faculty of Medicine and Health Sciences, Norwegian University of Science and Technology, 7491 Trondheim, Norway; 14 Department of Clinical and Molecular Medicine, Norwegian University of Science and Technology, 7491 Trondheim, Norway; 15 BioCore-Bioinformatics Core Facility, Norwegian University of Science and Technology, 7030 Trondheim, Norway; 16 Clinic of Laboratory Medicine, St. Olavs Hospital, Trondheim University Hospital, 7030 Trondheim, Norway; 17 Department of Public Health and Nursing, Norwegian University of Science and Technology, 7491 Trondheim, Norway; 18 Department of Clinical Services, St. Olav’s Hospital, Trondheim University Hospital, 7030 Trondheim, Norway

**Keywords:** urgency urinary incontinence (UUI), molecular landscape, amyloid precursor protein (APP) processing, Alzheimer’s disease

## Abstract

Urgency urinary incontinence (UUI) and Alzheimer’s disease (AD) are highly comorbid conditions in women, but the underlying molecular mechanisms are largely unknown. Therefore, we used network enrichment analyses and an elaborate literature search to integrate the most significant genes from four genome-wide association studies (GWASs) and other genetic, expression and functional evidence into a molecular landscape of female UUI. This molecular landscape centers around local, i.e., bladder-based, processing of the AD-associated amyloid precursor protein (APP). To further elucidate how APP processing is implicated in the comorbidity between UUI and AD, we conducted polygenic risk score (PRS)-based analyses, which showed that genetic risk factors associated with AD and a decreased amyloid beta 42/40 blood level ratio (also) contribute to UUI susceptibility. In conclusion, APP processing constitutes a putative molecular link between UUI and AD, adding further weight to their clinical comorbidity and having implications for the treatment (and prevention) of both traits.

## 1. Introduction

UUI can be defined as an involuntary loss of urine, associated with a compelling desire to void [1]. UUI is considered a symptom of the overarching overactive bladder syndrome (OAB) [1], which consists of several clinical subphenotypes that are based on various putative underlying mechanisms, comorbidities and contributing factors [2]. Furthermore, the prevalence of UUI varies widely depending on the definition and population examined. Estimates range between 1.7–36.4% and it increases with age [3]. In addition, UUI is more prevalent in women than men [3,4]. In men, bladder outlet obstruction, often due to benign prostatic hyperplasia, plays an important role in UUI etiology [5]. Therefore, the current study will address UUI in the female gender only.

The etiology of UUI is largely unknown, in part because of the clinical heterogeneity of the overarching OAB phenotype [2]. Multiple factors could contribute to developing OAB: urothelial and suburothelial dysfunction [2,6], myogenic dysfunction [2], urethra-related OAB [2,6], low bladder compliance [6], chronic bladder ischemia [6], chronic bladder inflammation [6], central sensitization [6], and OAB originating from the brain and brainstem [2,6]. These factors could also interact with and reinforce each other and likely influence OAB treatment outcome [2]. It is likely that UUI has a multifactorial etiology, with both environmental and genetic factors playing a role [2,4], but a thorough understanding of the molecular mechanisms underlying UUI is essentially lacking.

Alzheimer’s disease (AD), the most common form of dementia and a neurodegenerative disorder that results in a progressive decline of memory and cognitive functions [7], is often comorbid with UUI, and there is a positive correlation between their progression stages [8,9,10]. One (partial) explanation for diseases that are comorbid with AD, such as UUI, becoming more prevalent is the increasing age of the world’s population, partially because of increased life expectancy rates and decreased fertility rates [11]. In this respect, both AD [12] and UUI [13] negatively affect quality of life and independently raise the risk of falls and admissions to nursing homes [3,4,5]. Moreover, the risk of urinary incontinence is higher in AD patients compared to people with normal cognitive function (hazard ratio: 1.54, 95% confidence interval: 1.13–2.09) [14].

Aberrant processing of amyloid precursor protein (APP) is one of the key pathophysiological mechanisms contributing to AD [7]. Specifically, APP-derived amyloid beta (Aβ) peptides are the products of APP processing, and Aβ peptides aggregate to form extraneuronal amyloid plaques, one of the two main pathological features of AD. The two most common forms of Aβ are those peptides with 40 and 42 amino acids, i.e., Aβ40 and Aβ42, with Aβ42 having a greater propensity to aggregate—and hence form pathological plaques—than Aβ40 [7].

The current study aims at integrating the most significant genetic findings for UUI into a ‘molecular landscape’ in order to identify the main molecular processes contributing to UUI and, importantly and especially, how these processes relate to its comorbidity with AD (and APP processing).

## 2. Results

### 2.1. Landscape Input Genes

In total, 154 unique genes from the four GWASs of female UUI met our criterion of a ‘best’ gene-wide *p* value of <1.00 × 10^−3^ in at least one GWAS, i.e., *p* < 1.00 × 10^−3^ for “gene only’ or “gene and 100 kb up- and downstream flanking regions”. These 154 genes were used as input data for the landscape: 36 genes from the GWAS by Richter et al. (update) [15], 44 genes from the GWAS by Penney et al. [16], 49 genes from the GWAS by Cartwright et al. [17], and 26 genes from the GWAS in the HUNT sample [18]. Table 1 shows the 154 genes and their best gene-wide *p* values for each of the four GWASs and for the meta-analysis of the GWASs by Penney et al. [16], Cartwright et al. [17], and in the HUNT sample [18]. One gene, *NHLRC1*, had a gene-wide *p* value < 1.00 × 10^−3^ in two GWASs (Richter et al. (update) [15] and Cartwright et al. [17]). In addition, of the 154 candidate genes, 83 (54%, including *NHLRC1*) also had a nominally significant gene-wide *p* < 0.05 in at least one of the other three GWASs and/or the meta-analysis (Table 1). This considerable overlap between the genes emerging from the different GWASs at the nominally significant level implies that in different populations of European ancestry and despite other differences between the GWASs—e.g., in sample size and UUI phenotype definition—the same molecular processes are at least to some extent involved in UUI.

### 2.2. Enriched Networks

Our analysis showed that nine networks—containing at least two genes/proteins—were enriched within the 154 input genes, of which six networks were overlapping (Table 2). In Supplementary Figure S1, the radial representation of the large network that merged these six networks is shown, and amyloid precursor protein (APP) is the central protein in this network.

### 2.3. The Molecular Landscape of UUI

**Figure 1:**
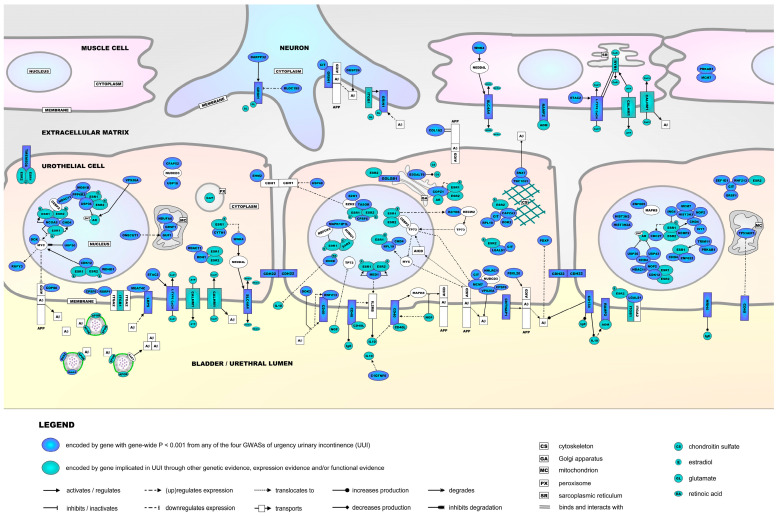
Molecular landscape of UUI. There are two main functional themes within the landscape: local, bladder-based processing of amyloid precursor protein (APP) and female sex hormone signaling. Firstly, APP is located in the cell membrane of urothelial cells, as well as the (innervating) neurons and muscle cells surrounding these cells. Several cytoplasmic, membrane and extracellular proteins in the landscape are involved in the degradation and processing of APP into extracellular amyloid beta (Aβ) peptides and the APP intracellular domain (AICD) that functions as a nuclear transcription factor. Multiple landscape proteins also functionally interact and/or are regulated by secreted Aβ in the urine and the extracellular matrix between urothelial cells, neurons, and bladder muscle cells. Secondly, in addition to APP signaling, estrogen receptor signaling plays an important role in the molecular landscape. Specifically, when bound by the active female sex hormone estradiol, the estrogen receptors 1 and 2 (ESR1 and ESR2) control multiple proteins and signaling cascades in the landscape, including the regulation of gene expression (in the nucleus) and forming functional complexes with other landscape proteins (in the cytoplasm). A more detailed description of all interactions in the landscape is provided in the Supplementary Text S1, and in Supplementary Figure S2a–c, the specific interactions in and around urothelial cells, a neuron, and muscle cells are shown, respectively.

Based on the network enrichment analysis and an extensive literature search, we built a molecular landscape of UUI that contains 111 interacting proteins and nine molecules (ATP, bicarbonate, calcium, chondroitin sulfate, dihydrotestosterone, estradiol, glutamate, IgE and retinoic acid) (Figure 1). In total, 82 (53%) of the 154 GWAS candidate genes encode proteins that could be included in the landscape. In addition, 12 proteins that have been implicated in UUI through other evidence could be placed in the landscape, while 15 other proteins were added although they have not been directly linked to UUI (yet), but show multiple functional interactions within the landscape (Supplementary Table S1) [15,16,17,19,20,21,22,23,24,25,26,27,28,29,30,31,32,33,34,35,36,37,38]. Of note, 12 of these 27 additional proteins (44%) are encoded by genes that are nominally significantly associated with UUI (gene-wide *p* < 0.05) in at least one of the four GWASs and/or the meta-analysis of the GWASs by Penney et al., Cartwright et al., and in the HUNT sample (Supplementary Table S1). Supplementary Table S2 shows the corroborating evidence for the link(s) of the nine abovementioned molecules with UUI [24,25,26,29,30,31,39,40,41,42,43,44,45,46,47,48].

Based on all gathered information, the molecular landscape was drawn within urothelial cells, muscle cells, a neuron, and their surrounding extracellular matrix. There are two main functional themes within the landscape, i.e., local, bladder-based APP processing and female sex hormone signaling. In Supplementary Text S1, a detailed description of all interactions in the landscape is provided [2,6,19,20,21,23,24,25,26,27,28,35,36,37,38,39,40,41,42,43,44,45,46,47,48,49,50,51,52,53,54,55,56,57,58,59,60,61,62,63,64,65,66,67,68,69,70,71,72,73,74,75,76,77,78,79,80,81,82,83,84,85,86,87,88,89,90,91,92,93,94,95,96,97,98,99,100,101,102,103,104,105,106,107,108,109,110,111,112,113,114,115,116,117,118,119,120,121,122,123,124,125,126,127,128,129,130,131,132,133,134,135,136,137,138,139,140,141,142,143,144,145,146,147,148,149,150,151,152,153,154,155,156,157,158,159,160,161,162,163,164,165,166,167,168,169,170,171,172,173,174,175,176,177,178,179,180,181,182,183,184,185,186,187,188,189,190,191,192,193,194,195,196,197,198,199,200,201,202,203,204,205,206,207,208,209,210,211,212,213,214,215,216,217,218,219,220,221,222,223,224,225,226,227,228,229,230,231,232,233,234,235]. In Supplementary Figure S2a–c, the specific interactions in and around urothelial cells, a neuron, and muscle cells are shown, respectively. In addition, we have provided a short description of the two main functional landscape themes in the legend of Figure 1 below.

### 2.4. PRS-Based Analyses

A complete overview of the PRS-based analyses is provided in Supplementary Table S3 and S4. After correcting for multiple testing, we identified significant genetic sharing/overlap between AD, based on the GWAS by Bellenguez et al. [236], and UUI, based on the GWAS by Cartwright et al. (*p*T = 0.05, *p* = 4.85 × 10^−8^; Table 3, Supplementary Figure S3) [17]. The subsequent SECA analysis for this PRS-based analysis revealed a positive genetic concordance, i.e., implying that genetic risk factors for AD also contribute to an increased risk of UUI. In addition, we found significant genetic sharing between the Aβ42/Aβ40 blood level ratio, based on the GWAS by Damotte et al. [237] and UUI, based on the GWAS by Cartwright et al. [17] (*p*T = 0.1, *p* = 2.31 × 10^−3^; Table 3, Supplementary Figure S4), with the SECA analysis revealing a negative genetic concordance. This indicates that genetic risk factors contributing to a decreased Aβ42/Aβ40 blood level ratio also contribute to an increased risk of UUI.

## 3. Discussion

To increase our knowledge of the molecular mechanisms governing UUI, which is highly comorbid with AD, a molecular landscape was built of female UUI that contains 111 interacting proteins and nine additional signaling molecules (Figure 1). The landscape provides insights into the mechanisms and processes that are dysregulated in UUI and it converges on local, i.e., bladder-based, APP processing by urothelial cells. APP is a transmembrane protein that is involved in multiple neuronal and non-neuronal processes, such as (neuronal) development, cell proliferation and differentiation, transcriptional regulation, and metabolic processes [239]. APP can be ‘processed’ through cleavage by proteases into a number of small peptides that have distinct physiological properties and functions. There are three major isoforms of APP that can all be processed into amyloid beta (Aβ): APP695 is (predominantly) expressed in neurons while APP751 and APP770 are expressed in multiple tissues outside of the brain [240].

APP has been extensively studied in the context of AD. In the brain, the APP-derived Aβ peptide is a major constituent of the extraneuronal Aβ plaques that, together with intraneuronal tangles that contain a high amount of phosphorylated tau proteins, are characteristic of the disease and—ultimately—lead to the loss of neurons and synapses [7,239]. The role of Aβ plaques in AD pathology is not fully understood. Other fragments resulting from APP processing, such as the APP intracellular domain (AICD) that functions as a transcription factor, may also play a role in AD pathophysiology [239].

The landscape contains multiple protein interactions in and around the cell membrane of urothelial cells that converge on local APP processing and/or involve (urinary) Aβ. In addition, some landscape proteins regulate or are involved in APP signaling and processing in (innervating) neurons and muscle cells surrounding urothelial cells. In this respect and although APP processing by cells other than neurons has been studied less, the non-neuronal isoforms of APP (APP751 and APP770) have been found to be (also) expressed in urothelial cells (https://www.proteinatlas.org, accessed on 15 January 2024). Moreover, non-neuronal APP-isoforms [77] and processed APP fragments, including Aβ [241], have been detected in the urine of both AD patients and cognitively healthy individuals. Further, determining urinary Aβ levels has been proposed as a non-invasive detection and monitoring tool for Alzheimer’s disease [241], with higher levels indicating disease progression. In this respect, approximately half of the Aβ produced in the brain of healthy individuals is transported to the peripheral blood and subsequently cleared into the urine by the kidneys [242].

Importantly, as already indicated above, there are clinical links between UUI and AD (which has aberrant APP processing as one of its main pathophysiological characteristics), as they are often comorbid and there is a positive correlation between their progression stages [8]. Previously, the comorbidity of UUI symptoms in AD patients was thought to be mostly due to the loss of cognitive and social control associated with AD. However, recent research suggests that discoordination of the bladder muscle in APP transgenic mice—a frequently used animal model of AD—due to functionally deficient autonomic, adrenergic neurons that innervate bladder muscle cells, at least partially contributes to the development of UUI symptoms [243]. Interestingly, the authors of this study speculate that the adrenergic neurons become functionally deficient due to local amyloidosis, i.e., deposits of Aβ plaques around these neurons [243]. This is in keeping with our UUI landscape, where Aβ could not only be produced by urothelial cells but also by afferent neurons and hence have an (additional) effect on bladder muscle cells. Moreover, Aβ reduces acetylcholine synthesis and release [244], and there is an inverse relationship between cholinergic signaling and Aβ accumulation [245]. In this respect, it is interesting that muscarinic receptors, which are both located in central nervous system (CNS) neurons and urothelial cells of the bladder, as well as their surrounding muscle cells and (peripheral) neurons, are treatment targets for both UUI and AD [246]. Intriguingly, the use of antimuscarinics (a type of anticholinergics) to reduce the symptoms of OAB/UUI has been associated with symptoms of dementia, such as cognitive decline [246,247,248] and, conversely, procholinergics that are used for treating AD could worsen OAB/UUI [246].

Given the observed clinical links between AD and UUI, it is interesting that we found that genetic risk factors associated with AD also contribute to a higher risk of UUI. In addition, we identified genetic overlap between a decreased Aβ42/Aβ40 blood level ratio and UUI. A decreased Aβ42/Aβ40 blood level ratio is both a measure of altered APP processing and is associated with an increased risk of (developing) AD. Therefore, altered APP processing, be it locally in the bladder or CNS based, constitutes a potential molecular link between UUI and AD, adding further weight to their clinical comorbidity.

Apart from APP processing, the molecular landscape indicates a role for (especially) female sex hormone signaling in UUI pathophysiology. The estrogen receptors 1 and 2 (ESR1 and ESR2), when bound by the active female sex hormone estradiol, control multiple proteins and signaling cascades in the landscape. Interestingly, sex hormone deficiency has been previously associated with (female) UUI [2] and estrogen therapy can be useful for relieving (female) OAB symptoms [249]. In addition, there is an increased risk of AD progression due to decreased estrogen levels in (aging) women after the menopause [245,250]. Nevertheless, the precise role of (female) sex hormone signaling in the pathophysiology of UUI is still unclear and further studies are needed to understand how altered sex hormone signaling could affect the molecular mechanisms underlying UUI, including altered APP processing, and interact with or be affected by other known risk factors for UUI such as obesity and aging.

Taken together, the current findings point towards a putative involvement of local—i.e., bladder/urothelial cell-based—APP processing in (female) UUI etiology, as well as genetic and molecular links between UUI and AD. This holds high potential for developing personalized therapeutic and preventive strategies. In this respect, women with UUI and an increased genetic predisposition towards AD should preferentially not receive anticholinergic treatment for their UUI symptoms, as this may provoke or worsen AD symptoms. Likewise, women with AD and an increased genetic risk for developing UUI should ideally not receive procholinergics, as these may provoke or worsen UUI symptoms. However, further validation studies in independent clinical cohorts and studies examining the exact contribution of local APP processing to UUI and establishing direct causal links between APP processing and UUI are needed before our findings could be further translated into novel preventive and treatment strategies. The molecular processes as described in the molecular landscape of UUI could be used as a starting point for this research. For example, a follow-up study could collect a new cohort of—in the first instance—women with UUI from whom blood samples would be collected for (1) genotyping and PRS-based analyses, and (2) determining the blood levels of Aβ40 and Aβ42, both at baseline and 12 and 24 months later, when the women would also undergo a cognitive assessment to identify (early) symptoms of AD. Through combining the PRS-based analysis results with the measured blood levels of Aβ40 and Aβ42 and the results of the cognitive assessments, we could then validate our findings of genetic overlap between AD/APP processing and UUI.

The findings from this study must be interpreted in light of some strengths and limitations. A particular strength is that this study is the first to integrate the results from four independent GWASs, including two updated or new GWASs, with genes/proteins implicated in UUI into a single, testable model, i.e., the molecular landscape of UUI. In addition, through the PRS-based analyses, we were able to add further weight to both the hypothesis of local, bladder-based APP processing (by both urothelial cells and neurons) playing a role in UUI etiology and the often observed clinical comorbidity between UUI and AD. A limitation of this study is that most of the genes that we considered for inclusion in the landscape are only based on common genetic variants associated with UUI, i.e., from GWASs. Studies on rare genetic variants contributing to UUI have not been conducted, but could provide additional UUI candidate genes. In addition, although all GWAS analyses were restricted to women of European ancestry, providing some homogeneity, they (still) had limited power to detect multiple significantly associated and overlapping genes, although there is considerably more overlap at the nominally significant level. This could be at least partially due to low sample sizes for the individual GWASs, heterogeneous and self-reported UUI phenotypes, or different definitions of the UUI and control groups. As such, future genetic studies on UUI, preferably with large sample sizes and objectively confirmed—and based on the same definition—UUI (and AD) may provide a more comprehensive and robust list of UUI-associated variants. Lastly, as no or not enough specific GWASs and other genetic data are currently available for other ethnicities (other than of European ancestry) and males with UUI, the findings from our study may not be directly generalizable to these groups. Therefore, future GWASs and other genetic studies—of sufficiently large size and with a uniform UUI phenotype/diagnosis—are also needed, and further analyses of the data from these studies could then help in determining whether APP processing, estrogen receptor signaling and/or other molecular mechanisms (also) contribute to UUI in these groups.

## 4. Materials and Methods

### 4.1. Selection of Landscape Input Genes

Significant results from four genome-wide association studies (GWASs) were used as main input data for building the molecular landscape of female UUI. Specifically, we used data from two previously published GWASs (Penney et al. and Cartwright et al.) [16,17], a re-analysis of a previously published GWAS, henceforth referred to as Richter et al. (update) from the Women’s Health Initiative Genomics & Randomized Trials Network (GARNET) [15], and one new GWAS conducted in a sample from the Trøndelag Health Study (HUNT) study [18], all including female UUI patients and controls. To allow for a comparison between the results, we restricted the GWAS analyses to women of European ancestry. Table 4 shows the main characteristics of the four GWASs. The methodology that was used to conduct each of the GWASs is described in more detail in Supplementary Text S2 [15,16,17,18,251,252,253,254,255,256,257,258,259,260,261,262,263,264]. This Supplementary Text includes more information about the UUI phenotype, genotyping platform(s), quality control criteria, study design, and statistical analytic methods. As can be derived from Table 4 and Supplementary Text S2, there are some differences between the four GWASs—e.g., in sample size and UUI phenotype definition—but also some similarities, e.g., in that all GWASs studied women of European ancestry and in the possibly confounding factors that were adjusted for.

All participants of the four GWASs gave their informed consent after the nature and possible consequences of their participation were explained. In addition, we conducted a meta-analysis using the results from the GWASs by Penney et al. [16], Cartwright et al. [17], and in the HUNT sample [18], as described in Supplementary Text S2 [264].

We then used the summary statistics data from each of the four GWASs to conduct gene-wide analyses using the MAGMA tool [265]. MAGMA combines multiple single nucleotide polymorphisms (SNPs) that are mapped to a gene and its upstream and downstream flanking regions, while adjusting for the linkage disequilibrium between those SNPs and tests the joint association of all SNPs in the (vicinity of the) gene with the phenotype, resulting in a single gene-wide *p* value for each gene. Evidence suggests that expression quantitative trait loci (eQTLs), genetic variants surrounding a gene, up to approximately 100 kilobase (kb) pair upstream and downstream from a gene can influence its expression [266,267]. Therefore, we conducted MAGMA analyses for the SNPs within all protein-coding genes only (without flanking regions) and the SNPs within each gene plus 100 kb up- and downstream flanking regions. Experimental evidence has revealed that single SNPs in (the vicinity of) a gene with subthreshold *p* values as high as 1.00 × 10^−4^, which is considered as suggestive evidence of genetic association, can represent truly associated variants that have an effect on gene expression and function [266,267]. Therefore, we considered the protein-coding genes with a ‘best’ gene-wide *p* value—i.e., the ‘weighted’ *p* value for all SNPs within the gene itself (“gene only”) or for the SNPs within the gene and 100 kb up- and downstream flanking regions (“gene and 100 kb up- and downstream flanking regions”)—of <1.00 × 10^−3^ as potentially associated genes for further analyses and building of the landscape (see below).

### 4.2. Network Enrichment Analysis

Subsequently, a network enrichment analysis of all candidate genes from the UUI GWASs was conducted using the Ingenuity pathway analysis (IPA) software package (QIAGEN, Aarhus, Denmark) (http://www.ingenuity.com, accessed on 9 January 2023).

### 4.3. Building the Molecular Landscape of UUI

To build the molecular landscape of UUI, we applied an approach that we used previously [268]. The UniProt Protein Knowledge Base (http://www.uniprot.org/uniprot, accessed on 23 January 2023) was used to gather basic information on the function(s) and subcellular localization(s) of all the landscape candidate genes/proteins [20]. In addition, we used PubMed (http://www.ncbi.nlm.nih.gov/sites/entrez), accessed on 30 January 2023) to identify the functional, experimental evidence-based interactions between the landscape candidate proteins, and with proteins implicated in UUI through other evidence, including candidate gene association studies, mRNA/protein expression studies and/or functional studies. This ensured inclusion of genes/proteins with a direct link with UUI and/or genes/proteins with functional interactions with UUI-implicated proteins. If possible, we also included proteins that are (functionally) linked to the results from the network enrichment analysis.

Based on these results, the landscape was then drawn using the program Serif version 4.0. In line with previous research and based on the knowledge about the function(s) and location(s) of the candidate proteins, the landscape contains urothelial cells, muscle cells, and a neuron. In this respect, the Human Protein Atlas (https://www.proteinatlas.org, accessed on 6 February 2023) and PubMed were screened to check for the functional interaction and tissue/cell expression profiles of the landscape candidate genes/proteins in pelvic tissues, in order to determine their most likely location in the landscape. Repetitive drawing of protein-protein interactions was avoided as much as possible. If multiple locations of a protein-protein interaction were possible, functional interaction and expression data or other protein-protein interactions were used to identify the (most) appropriate location.

### 4.4. PRS-Based Analyses

By performing polygenic risk score (PRS)-based analyses, we first assessed the presence and level of genetic overlap (or shared genetic etiology) between AD and UUI. For this, the publicly available summary statistics data from three GWASs of AD—i.e., the study by the FinnGen consortium (https://www.finngen.fi/en/access_results, accessed on 20 March 2023), Jansen et al. [238], and Bellenguez et al. [236]—were used, as well as the summary statistics from all four GWASs of UUI. Secondly, similar PRS-based analyses were performed to assess the presence and level of genetic overlap between the ratio of the blood levels of Aβ42 and Aβ40—with a decreased Aβ42/Aβ40 blood level ratio increasing the risk of developing AD [269]—and UUI, using the publicly available summary statistics data of the GWAS of the Aβ42/40 blood level ratio by Damotte et al. [237] and the GWASs by Penney et al. [16], Cartwright et al. [17], and in the HUNT sample [18]. Further details about the methodology of the PRS-based analyses are provided in Supplementary Text S3 [16,17,236,237,238,270,271,272,273,274].

## 5. Conclusions

The molecular landscape that we built implicates a putative role for APP processing in female UUI and provides novel molecular insights that increase our understanding of UUI etiology. Further studies validating and examining the exact contributions of the proposed molecular pathways in relation to UUI etiology are needed in order to develop new preventative and therapeutic strategies for this burdensome problem.

## Figures and Tables

**Table 1:** The 154 UUI candidate genes that were selected based on a ‘best’ gene-wide *p* value < 1.00 × 10^−3^ in any of the four GWASs of UUI—indicated by *—are shown. For each best *p* value, it is also indicated whether it applies to the gene only (a) or the gene and 100 kb up- and downstream flanking regions (b). For 83 of the 154 selected genes (incl. *NHLRC1*), the gene also showed a nominally significant gene-wide *p* < 0.05 in at least one other UUI GWAS and/or the meta-analysis of the GWASs by Penney et al. [16], Cartwright et al. [17], and in the HUNT sample [18], which is indicated by †. Further, all genes encoding proteins that operate in the molecular landscape of UUI (see below) are indicated in **bold**. “NA” indicates that the *p*-values are ‘not available’, as they could not be computed.

	Best *p* Values				
Gene Name	Richter et al. (Update)	Penney et al.	Cartwright et al.	HUNT	Meta GWAS
*ACSS3*	9.42 × 10^−1^	9.26 × 10^−1^	9.95 × 10^−4^ *	5.65 × 10^−1^	4.36 × 10^−1^ (b)
*ADAMTSL3*	9.65 × 10^−1^	8.12 × 10^−4^ *	2.10 × 10^−1^	3.87 × 10^−1^	4.22 × 10^−1^
*ARHGAP24*	9.42 × 10^−1^	5.91 × 10^−4^ *	9.43 × 10^−1^	1.17 × 10^−1^	5.65 × 10^−3^ † (a)
*ASB4*	4.67 × 10^−1^	1.96 × 10^−4^ *	5.60 × 10^−1^	3.38 × 10^−1^	2.77 × 10^−2^ †
***B3GALT6***	2.29 × 10^−1^	2.10 × 10^−1^	4.51 × 10^−4^ *	6.80 × 10^−1^	3.94 × 10^−2^ †
*BCOX1*	3.03 × 10^−1^	8.52 × 10^−4^ *	8.74 × 10^−1^	NA	NA
***BDH1***	7.37 × 10^−1^	6.94 × 10^−1^	4.86 × 10^−2^ †	8.06 × 10^−4^ *	2.59 × 10^−2^ †
***BLOC1S5***	2.07 × 10^−1^	1.05 × 10^−1^	6.21 × 10^−1^	7.15 × 10^−4^ *	6.58 × 10^−1^
*BTNL10*	1.10 × 10^−4^ *	3.62 × 10^−1^	3.81 × 10^−1^	5.59 × 10^−1^	NA
*C11orf16*	5.08 × 10^−1^	3.57 × 10^−1^	7.38 × 10^−1^	3.39 × 10^−4^ *	2.21 × 10^−1^
***C1QTNF6***	8.18 × 10^−1^	4.39 × 10^−1^	6.24 × 10^−4^ *	9.83 × 10^−1^	1.22 × 10^−1^
*C2orf70*	3.08 × 10^−1^	8.77 × 10^−1^	4.30 × 10^−2^ †	1.33 × 10^−4^ *	NA
***CACNB1***	2.52 × 10^−1^	9.06 × 10^−4^ *	9.24 × 10^−2^	1.25 × 10^−1^	2.42 × 10^−2^ †
*CADM2*	9.90 × 10^−2^	9.13 × 10^−4^ *	1.99 × 10^−1^	9.92 × 10^−1^	5.75 × 10^−2^
*CAPN13*	6.14 × 10^−1^	8.33 × 10^−2^	5.68 × 10^−1^	8.20 × 10^−4^ *	1.99 × 10^−2^ †
***CAPZA2***	2.25 × 10^−1^	5.87 × 10^−4^*	6.30 × 10^−1^	3.91 × 10^−1^	6.04 × 10^−3^ †
*CCDC151*	5.30 × 10^−5^ *	1.17 × 10^−1^	2.99 × 10^−1^	5.50 × 10^−1^	NA
*CCDC66*	9.63 × 10^−1^	9.70 × 10^−4^ *	7.09 × 10^−1^	7.19 × 10^−1^	7.74 × 10^−2^
***CD40***	5.38 × 10^−1^	1.97 × 10^−1^	2.04 × 10^−4^ *	3.55 × 10^−1^	1.48 × 10^−1^
***CDC27***	6.05 × 10^−1^	1.26 × 10^−1^	7.20 × 10^−1^	6.76 × 10^−4^ *	2.62 × 10^−2^ †
***CDH22***	2.17 × 10^−1^	6.66 × 10^−1^	8.07 × 10^−5^ *	2.69 × 10^−1^	2.24 × 10^−1^
***CDK12***	4.01 × 10^−2^ †	8.48 × 10^−5^ *	5.72 × 10^−1^	7.94 × 10^−1^	6.29 × 10^−3^ †
***CFAP52***	3.28 × 10^−1^	2.12 × 10^−1^	6.89 × 10^−4^ *	7.22 × 10^−1^	1.10 × 10^−1^
***CHD4***	2.09 × 10^−1^	2.57 × 10^−1^	1.46 × 10^−1^	5.71 × 10^−4^ *	2.86 × 10^−2^ †
***CIT***	2.36 × 10^−5^ *	4.85 × 10^−1^	5.02 × 10^−1^	8.35 × 10^−1^	6.29 × 10^−1^
*CLCA4*	8.69 × 10^−4^ *	1.13 × 10^−3^ †	2.51 × 10^−1^	1.00 × 10^−1^	1.65 × 10^−3^ †
***CNTNAP1***	3.42 × 10^−1^	7.49 × 10^−5^ *	8.64 × 10^−1^	2.75 × 10^−1^	4.41 × 10^−3^ †
***COL1A2***	1.92 × 10^−2^ †	4.38 × 10^−1^	1.84 × 10^−1^	7.66 × 10^−4^ *	1.14 × 10^−2^ †
***COPS6***	3.93 × 10^−1^	4.13 × 10^−1^	5.87 × 10^−4^ *	1.47 × 10^−1^	1.74 × 10^−1^
***COPZ1***	3.83 × 10^−1^	2.30 × 10^−1^	8.49 × 10^−1^	9.59 × 10^−4^ *	8.30 × 10^−1^
***CPSF6***	8.49 × 10^−4^ *	1.13 × 10^−1^	7.58 × 10^−1^	2.03 × 10^−1^	5.38 × 10^−1^
***CYTH1***	2.53 × 10^−1^	3.75 × 10^−4^ *	8.61 × 10^−1^	1.36 × 10^−1^	5.94 × 10^−2^
***DCK***	1.11 × 10^−4^ *	6.16 × 10^−1^	4.38 × 10^−1^	1.88 × 10^−1^	6.99 × 10^−1^
*DEAF1*	2.88 × 10^−4^ *	6.90 × 10^−1^	6.19 × 10^−1^	2.62 × 10^−1^	7.04 × 10^−1^
*DGAT2*	3.68 × 10^−1^	2.00 × 10^−1^	6.18 × 10^−1^	3.07 × 10^−4^ *	2.30 × 10^−2^ †
*DIRAS1*	7.75 × 10^−4^ *	2.66 × 10^−1^	2.17 × 10^−1^	4.53 × 10^−1^	8.59 × 10^−1^
***DOK2***	5.54 × 10^−2^	4.06 × 10^−4^ *	5.11 × 10^−1^	6.75 × 10^−1^	4.68 × 10^−1^
***DUSP26***	6.00 × 10^−4^ *	8.53 × 10^−1^	3.08 × 10^−1^	1.43 × 10^−1^	8.78 × 10^−1^
***EEF1E1***	4.67 × 10^−2^ †	1.58 × 10^−1^	8.80 × 10^−1^	8.10 × 10^−4^ *	4.18 × 10^−1^
*ELK3*	7.38 × 10^−1^	6.57 × 10^−1^	1.27 × 10^−1^	3.45 × 10^−4^ *	3.99 × 10^−1^
***EPB41L4B***	5.58 × 10^−1^	8.89 × 10^−4^ *	3.46 × 10^−1^	6.86 × 10^−2^	1.62 × 10^−3^ †
*EPS8L2*	2.43 × 10^−4^ *	2.91 × 10^−1^	4.89 × 10^−1^	4.29 × 10^−1^	2.53 × 10^−1^
***EZH1***	2.65 × 10^−1^	4.87 × 10^−4^ *	7.69 × 10^−1^	1.44 × 10^−1^	1.26 × 10^−3^ †
***FBXL20***	2.75 × 10^−2^ †	4.54 × 10^−5^ *	4.22 × 10^−1^	6.09 × 10^−1^	3.24 × 10^−3^ †
*FBXO33*	3.94 × 10^−1^	9.85 × 10^−4^ *	7.72 × 10^−1^	6.64 × 10^−2^	1.85 × 10^−1^
*GC*	1.64 × 10^−1^	3.60 × 10^−5^ *	2.70 × 10^−1^	3.11 × 10^−1^	6.79 × 10^−3^ †
***GOLGB1***	9.21 × 10^−4^ *	2.49 × 10^−1^	4.05 × 10^−2^ †	7.36 × 10^−1^	6.99 × 10^−1^
***GRIN1***	7.97 × 10^−2^	1.58 × 10^−4^ *	4.44 × 10^−1^	2.44 × 10^−1^	2.91 × 10^−2^ †
***GRSF1***	1.69 × 10^−4^ *	5.95 × 10^−1^	1.01 × 10^−1^	2.99 × 10^−1^	2.11 × 10^−1^
***GUF1***	7.75 × 10^−4^ *	5.33 × 10^−1^	7.69 × 10^−1^	2.86 × 10^−1^	8.31 × 10^−1^
***HDAC11***	6.90 × 10^−1^	1.67 × 10^−1^	8.41 × 10^−4^ *	3.91 × 10^−1^	4.63 × 10^−2^ †
***HIST3H2A***	5.95 × 10^−5^ *	6.98 × 10^−1^	8.26 × 10^−1^	4.95 × 10^−1^	NA
*HIST3H2BB*	6.32 × 10^−5^ *	6.95 × 10^−1^	8.35 × 10^−1^	3.51 × 10^−1^	NA
***HIST3H3***	8.92 × 10^−5^ *	6.38 × 10^−1^	8.22 × 10^−1^	3.02 × 10^−1^	NA
***HRH4***	4.96 × 10^−1^	2.11 × 10^−1^	2.25 × 10^−1^	6.09 × 10^−4^ *	1.98 × 10^−1^
*IFFO1*	3.86 × 10^−1^	2.65 × 10^−1^	3.22 × 10^−2^ †	7.23 × 10^−4^ *	5.59 × 10^−2^
***ING4***	6.61 × 10^−1^	7.61 × 10^−2^	3.52 × 10^−1^	8.45 × 10^−4^ *	2.41 × 10^−2^ †
*IQCF2*	2.22 × 10^−1^	7.44 × 10^−1^	8.94 × 10^−4^ *	6.47 × 10^−1^	1.06 × 10^−2^ †
*IQCF5*	7.99 × 10^−1^	7.57 × 10^−1^	8.25 × 10^−4^ *	6.24 × 10^−1^	1.12 × 10^−2^ †
***ISY1***	8.77 × 10^−4^ *	5.23 × 10^−1^	1.74 × 10^−1^	1.26 × 10^−1^	7.96 × 10^−3^ †
***KCTD6***	6.44 × 10^−1^	5.08 × 10^−1^	7.62 × 10^−4^ *	3.78 × 10^−1^	8.36 × 10^−2^
***LGALS1***	2.11 × 10^−1^	9.24 × 10^−1^	5.32 × 10^−4^ *	1.68 × 10^−1^	2.64 × 10^−2^ †
*LMCD1*	1.58 × 10^−1^	7.54 × 10^−1^	2.61 × 10^−1^	9.47 × 10^−4^*	2.00 × 10^−1^
***LRP5***	1.35 × 10^−1^	5.33 × 10^−4^ *	1.31 × 10^−1^	1.90 × 10^−1^	5.52 × 10^−2^
***MAPK1IP1L***	3.89 × 10^−1^	2.48 × 10^−1^	1.62 × 10^−6^ *	5.86 × 10^−1^	1.01 × 10^−2^ †
***MCM7***	4.12 × 10^−1^	3.31 × 10^−1^	8.40 × 10^−4^ *	1.18 × 10^−1^	2.57 × 10^−1^
***MED1***	3.94 × 10^−2^ †	5.11 × 10^−5^ *	6.67 × 10^−1^	7.39 × 10^−1^	3.16 × 10^−3^ †
***MGAT4C***	6.03 × 10^−1^	2.50 × 10^−4^ *	8.34 × 10^−1^	6.22 × 10^−1^	1.10 × 10^−1^
***MOB1B***	1.86 × 10^−5^ *	4.97 × 10^−1^	1.67 × 10^−1^	2.74 × 10^−1^	5.59 × 10^−1^
*MRGPRX3*	5.27 × 10^−1^	2.61 × 10^−1^	2.36 × 10^−4^ *	5.36 × 10^−1^	4.49 × 10^−2^ †
*MRGPRX4*	4.15 × 10^−1^	5.06 × 10^−1^	2.15 × 10^−4^ *	3.81 × 10^−1^	2.34 × 10^−2^ †
***MTTP***	3.14 × 10^−1^	1.07 × 10^−1^	9.35 × 10^−4^ *	3.37 × 10^−1^	1.01 × 10^−2^ †
*NAGA*	4.07 × 10^−1^	2.18 × 10^−4^ *	2.59 × 10^−1^	9.18 × 10^−2^	1.78 × 10^−2^ †
*NAGLU*	8.22 × 10^−2^	8.70 × 10^−4^ *	8.55 × 10^−1^	2.47 × 10^−1^	1.43 × 10^−1^
***NCBP2***	6.98 × 10^−1^	2.22 × 10^−1^	1.29 × 10^−1^	4.40 × 10^−4^ *	1.17 × 10^−1^
***NCOA5***	5.06 × 10^−1^	3.01 × 10^−1^	9.95 × 10^−4^ *	4.48 × 10^−1^	2.11 × 10^−2^ †
***NDUFA6***	6.19 × 10^−1^	3.25 × 10^−4^*	2.21 × 10^−1^	2.00 × 10^−1^	2.80 × 10^−2^ †
*NEUROD2*	1.19 × 10^−1^	1.37 × 10^−4^*	1.22 × 10^−1^	6.95 × 10^−1^	1.38 × 10^−2^ †
***NHLRC1***	4.03 × 10^−4^ *	5.94 × 10^−1^	4.28 × 10^−4^*	7.39 × 10^−1^	9.91 × 10^−3^ †
*NLRP12*	3.78 × 10^−1^	4.71 × 10^−1^	2.78 × 10^−1^	8.80 × 10^−4^*	2.36 × 10^−1^
***NOP2***	4.40 × 10^−1^	2.24 × 10^−1^	1.33 × 10^−1^	8.19 × 10^−4^*	2.58 × 10^−2^ †
*NOX3*	7.82 × 10^−4^ *	5.62 × 10^−1^	8.02 × 10^−1^	2.51 × 10^−2^ †	3.89 × 10^−1^
*NUBP1*	1.02 × 10^−2^ †	4.91 × 10^−1^	9.88 × 10^−5^ *	2.44 × 10^−1^	2.28 × 10^−1^
***ONECUT1***	9.57 × 10^−3^ †	2.79 × 10^−1^	4.03 × 10^−4^ *	1.23 × 10^−1^	NA
*OR1M1*	3.55 × 10^−1^	3.44 × 10^−1^	2.25 × 10^−4^ *	5.97 × 10^−2^	2.61 × 10^−2^ †
*OR51E1*	4.80 × 10^−1^	8.04 × 10^−1^	8.80 × 10^−4^ *	7.94 × 10^−1^	6.48 × 10^−2^
*OR7G1*	4.52 × 10^−1^	3.07 × 10^−1^	6.24 × 10^−4^ *	6.57 × 10^−2^	1.39 × 10^−2^ †
*OR7G2*	4.81 × 10^−1^	2.16 × 10^−1^	8.70 × 10^−4^ *	6.55 × 10^−2^	3.97 × 10^−2^ †
*OR7G3*	4.52 × 10^−1^	3.81 × 10^−1^	5.09 × 10^−4^ *	4.50 × 10^−2^ †	7.69 × 10^−2^
*PDDC1*	9.87 × 10^−4^ *	8.29 × 10^−1^	5.27 × 10^−1^	7.71 × 10^−1^	NA
***PDXP***	7.38 × 10^−2^	8.75 × 10^−1^	4.15 × 10^−4^ *	3.15 × 10^−1^	2.13 × 10^−2^ †
*PHETA2*	5.96 × 10^−1^	4.26 × 10^−4^ *	2.53 × 10^−1^	1.44 × 10^−1^	2.34 × 10^−2^ †
***PPP1R1B***	1.43 × 10^−1^	6.08 × 10^−4^ *	1.29 × 10^−1^	2.30 × 10^−1^	1.84 × 10^−2^ †
***PPP6R3***	3.21 × 10^−2^ †	5.20 × 10^−4^ *	2.01 × 10^−1^	4.83 × 10^−1^	3.81 × 10^−2^ †
***PRKAB1***	8.46 × 10^−5^ *	5.89 × 10^−1^	1.32 × 10^−1^	8.58 × 10^−1^	2.07 × 10^−1^
*PYGO2*	8.56 × 10^−1^	7.39 × 10^−1^	1.31 × 10^−4^ *	3.20 × 10^−1^	1.34 × 10^−1^
*RAB43*	7.48 × 10^−4^ *	7.88 × 10^−1^	1.96 × 10^−1^	1.53 × 10^−1^	2.42 × 10^−2^ †
***RAMP2***	5.07 × 10^−1^	4.58 × 10^−4^ *	8.77 × 10^−1^	7.91 × 10^−3^ †	1.98 × 10^−3^ †
*RAPH1*	1.14 × 10^−1^	1.69 × 10^−1^	6.45 × 10^−1^	8.15 × 10^−4^ *	1.26 × 10^−1^
*RHCE*	5.19 × 10^−1^	6.48 × 10^−1^	3.29 × 10^−4^ *	9.39 × 10^−1^	1.00 × 10^−1^
*RHD*	7.15 × 10^−1^	8.61 × 10^−1^	2.86 × 10^−4^ *	9.32 × 10^−1^	2.43 × 10^−1^
*RNF180*	5.88 × 10^−4^ *	2.39 × 10^−1^	3.91 × 10^−1^	3.99 × 10^−1^	5.13 × 10^−1^
*RNF187*	8.39 × 10^−5^ *	1.96 × 10^−1^	6.83 × 10^−1^	5.19 × 10^−1^	5.33 × 10^−1^
***RNF213***	4.66 × 10^−4^ *	6.56 × 10^−1^	5.78 × 10^−1^	2.28 × 10^−1^	5.16 × 10^−1^
***RPL19***	2.55 × 10^−1^	5.33 × 10^−4^ *	3.12 × 10^−1^	2.29 × 10^−1^	2.38 × 10^−2^ †
***RSRP1***	6.32 × 10^−1^	9.26 × 10^−1^	9.80 × 10^−4^ *	3.43 × 10^−1^	6.19 × 10^−2^
***RUFY3***	4.15 × 10^−4^ *	4.63 × 10^−1^	6.87 × 10^−2^	2.37 × 10^−1^	3.68 × 10^−1^
***RXRB***	3.92 × 10^−2^ †	9.79 × 10^−4^ *	3.67 × 10^−1^	NA	9.96 × 10^−2^
***SAA2***	4.34 × 10^−1^	5.41 × 10^−1^	2.07 × 10^−4^ *	4.97 × 10^−1^	9.86 × 10^−2^
***SAA4***	4.59 × 10^−1^	4.43 × 10^−1^	1.50 × 10^−4^ *	3.74 × 10^−1^	8.49 × 10^−2^
*SAAL1*	5.24 × 10^−1^	7.45 × 10^−2^	3.38 × 10^−4^ *	4.53 × 10^−1^	4.58 × 10^−2^ †
*SERTAD4*	5.53 × 10^−1^	1.12 × 10^−1^	6.97 × 10^−1^	8.18 × 10^−4^ *	2.39 × 10^−2^ †
***SHISA7***	2.24 × 10^−2^ †	3.45 × 10^−4^ *	1.53 × 10^−1^	3.23 × 10^−1^	3.42 × 10^−3^ †
*SLC14A2*	6.01 × 10^−1^	1.12 × 10^−1^	3.59 × 10^−1^	7.97 × 10^−4^ *	3.12 × 10^−3^ †
*SLC39A3*	8.21 × 10^−4^ *	5.88 × 10^−1^	2.07 × 10^−1^	1.39 × 10^−1^	8.46 × 10^−1^
***SLC4A4***	1.30 × 10^−2^ †	2.39 × 10^−1^	6.78 × 10^−4^ *	5.22 × 10^−1^	4.30 × 10^−2^ †
*SMDT1*	4.78 × 10^−1^	3.14 × 10^−4^ *	2.59 × 10^−1^	5.42 × 10^−2^	2.52 × 10^−2^ †
***SNX7***	7.20 × 10^−1^	2.70 × 10^−1^	2.37 × 10^−1^	7.06 × 10^−4^ *	1.12 × 10^−3^ †
*SOCS4*	6.45 × 10^−1^	2.81 × 10^−1^	1.43 × 10^−4^ *	6.63 × 10^−1^	2.08 × 10^−2^ †
*SOX1*	5.38 × 10^−1^	1.68 × 10^−1^	7.51 × 10^−3^ †	4.23 × 10^−4^ *	1.62 × 10^−1^
*SPDYE1*	4.67 × 10^−2^ †	3.72 × 10^−1^	1.61 × 10^−4^*	2.87 × 10^−1^	4.51 × 10^−2^ †
*SSNA1*	7.69 × 10^−1^	6.63 × 10^−4^ *	7.89 × 10^−1^	3.49 × 10^−1^	1.02 × 10^−1^
*SSTR3*	6.14 × 10^−1^	6.77 × 10^−1^	7.78 × 10^−4^ *	9.58 × 10^−1^	1.49 × 10^−1^
***STAC2***	2.47 × 10^−1^	3.55 × 10^−4^ *	4.37 × 10^−2^ †	2.94 × 10^−1^	2.43 × 10^−2^ †
*STARD3*	1.55 × 10^−1^	2.78 × 10^−4^ *	4.14 × 10^−1^	6.00 × 10^−1^	2.46 × 10^−2^ †
***TASOR***	8.68 × 10^−1^	4.80 × 10^−4^ *	6.89 × 10^−1^	7.37 × 10^−1^	3.31 × 10^−2^ †
***TBC1D21***	2.27 × 10^−1^	7.98 × 10^−4^ *	5.33 × 10^−1^	6.68 × 10^−1^	3.65 × 10^−2^ †
*TEKT5*	2.30 × 10^−2^ †	6.87 × 10^−1^	1.21 × 10^−4^ *	1.54 × 10^−2^ †	1.06 × 10^−1^
*TLE1*	1.04 × 10^−1^	2.27 × 10^−1^	9.22 × 10^−5^ *	3.01 × 10^−1^	8.95 × 10^−2^
*TLL1*	1.91 × 10^−1^	9.98 × 10^−2^	4.21 × 10^−5^ *	7.54 × 10^−1^	3.99 × 10^−2^ †
*TMEM225*	1.52 × 10^−4^ *	7.03 × 10^−1^	3.82 × 10^−2^ †	1.60 × 10^−2^ †	3.63 × 10^−1^
*TMEM233*	1.47 × 10^−4^ *	6.69 × 10^−1^	4.32 × 10^−1^	5.30 × 10^−1^	5.57 × 10^−1^
***TMEM50A***	6.01 × 10^−1^	5.90 × 10^−1^	2.64 × 10^−4^ *	6.98 × 10^−1^	6.29 × 10^−2^
*TMEM80*	2.92 × 10^−4^ *	3.12 × 10^−1^	3.48 × 10^−1^	1.64 × 10^−1^	1.31 × 10^−2^ †
***TNFRSF4***	3.46 × 10^−1^	1.55 × 10^−1^	8.65 × 10^−4^ *	7.17 × 10^−1^	3.28 × 10^−1^
***TP53AIP1***	9.75 × 10^−4^ *	8.03 × 10^−1^	7.48 × 10^−1^	6.01 × 10^−1^	4.02 × 10^−1^
*TPMT*	6.55 × 10^−4^ *	1.62 × 10^−1^	1.34 × 10^−3^ †	8.20 × 10^−1^	9.73 × 10^−3^ †
***TRIM11***	1.25 × 10^−4^ *	4.69 × 10^−1^	8.66 × 10^−1^	2.35 × 10^−1^	2.99 × 10^−1^
*TRIM17*	1.48 × 10^−4^ *	2.81 × 10^−1^	8.64 × 10^−1^	2.60 × 10^−1^	1.50 × 10^−1^
*TRIM49D2*	4.92 × 10^−1^	8.80 × 10^−1^	1.41 × 10^−1^	9.78 × 10^−4^ *	9.53 × 10^−2^
*TVP23A*	1.57 × 10^−2^ †	6.00 × 10^−1^	1.39 × 10^−4^ *	4.42 × 10^−1^	1.41 × 10^−1^
***USP18***	1.15 × 10^−1^	5.88 × 10^−1^	7.73 × 10^−4^ *	6.11 × 10^−1^	2.71 × 10^−1^
***USP36***	2.09 × 10^−1^	9.27 × 10^−4^ *	9.67 × 10^−1^	1.84 × 10^−1^	2.34 × 10^−2^ †
***USP43***	1.06 × 10^−1^	5.59 × 10^−2^	7.00 × 10^−4^ *	9.41 × 10^−1^	2.80 × 10^−2^ †
***USP48***	6.18 × 10^−4^ *	2.36 × 10^−1^	2.22 × 10^−1^	1.18 × 10^−1^	1.31 × 10^−1^
*VPS25*	5.08 × 10^−1^	4.54 × 10^−4^ *	6.87 × 10^−1^	1.88 × 10^−1^	1.79 × 10^−3^ †
***VPS26A***	2.72 × 10^−1^	3.96 × 10^−4^ *	9.62 × 10^−1^	5.53 × 10^−1^	2.31 × 10^−2^ †
*WBP2NL*	6.34 × 10^−1^	6.08 × 10^−4^ *	2.36 × 10^−1^	6.27 × 10^−2^	6.77 × 10^−3^ †
***WDHD1***	8.64 × 10^−1^	4.30 × 10^−1^	7.05 × 10^−4^ *	8.06 × 10^−1^	4.87 × 10^−2^ †
***WNK4***	2.17 × 10^−1^	4.63 × 10^−4^ *	8.59 × 10^−1^	2.22 × 10^−1^	1.58 × 10^−3^ †
*XKR4*	4.30 × 10^−1^	6.73 × 10^−1^	2.07 × 10^−4^*	5.85 × 10^−1^	4.93 × 10^−1^
***ZNF605***	5.81 × 10^−1^	1.44 × 10^−1^	9.85 × 10^−5^ *	2.22 × 10^−1^	5.80 × 10^−2^
***ZNF622***	8.20 × 10^−1^	1.61 × 10^−1^	1.52 × 10^−1^	1.29 × 10^−4^ *	2.40 × 10^−1^
*ZSCAN20*	3.62 × 10^−1^	4.04 × 10^−4^ *	6.75 × 10^−1^	7.03 × 10^−2^	6.48 × 10^−3^ †

**Table 2:** Networks that are enriched in the 154 landscape candidate genes from the four GWASs of UUI. The genes encoding proteins that operate in the molecular landscape of UUI (Figure 1) are indicated in **bold**. In addition, the six overlapping networks that have been merged into the large network shown in Supplementary Figure S1 are designated with *.

Network	Genes	Score	Focus Molecules	Top Diseases and Functions
1	***B3GALT6***, ***CD40***, ***CDC27***, ***CDK12***, ***CYTH1***, ***DOK2***, *EPS8L2*, ***EZH1***, *GC*, ***LGALS1***, ***LRP5***, ***MED1***, ***MGAT4C***, ***MTTP***, *NAGLU*, *NOX3*, *OR51E1*, ***RUFY3***, ***SAA2***, ***SAA4***, *SAAL1*, *SOX1*, *TLL1*, ***TP53AIP1***, ***TRIM11***, ***WNK4***	1.00 × 10^−55^	26	Cellular Movement, Hematological System Development and Function, and Immune Cell Trafficking
2	***BDH1***, ***BLOC1S5***, ***C1QTNF6***, ***CHD4***, ***COPS6***, ***GRIN1***, ***HIST3H2A***, ***HIST3H3***, ***HDAC11***, ***ING4***, ***ISY1***, ***MOB1B***, ***NCOA5***, *NEUROD2*, ***NHLRC1***, ***NOP2***, ***PPP1R1B***, ***PPP6R3***, ***RNF213***, *SOCS4*, *STARD3*, ***USP18***, ***USP36***, ***USP43***, ***USP48***, ***ZNF622***	1.00 × 10^−55^	26	Cardiovascular Disease, Cell-To-Cell Signaling and Interaction, Nervous System Development and Function
3 *	*ACSS3*, *ASB4*, ***CAPZA2***, ***CIT***, ***CPSF6***, ***DCK***, *DGAT2*, ***EEF1E1***, ***GRSF1***, *HIST3H2BB*, ***MCM7***, ***NDUFA6***, ***ONECUT1***, ***PRKAB1***, ***RPL19***, ***RSRP1***, *SLC14A2*, *TEKT5*, *TLE1*, ***VPS26A***, ***WDHD1***	1.00 × 10^−41^	21	Developmental Disorder, DNA Replication, Recombination, and Repair, Hereditary Disorder
4 *	*ADAMTSL3*, *BCOX1*, *CADM2*, ***CFAP52***, ***CNTNAP1***, ***COPZ1***, ***EPB41L4B***, ***GOLGB1***, *IQCF2*, ***KCTD6***, ***NCBP2***, *OR1M1*, *PHETA2*, *SSNA1*, ***TASOR***, ***TMEM50A***	1.00 × 10^−29^	16	Cell Cycle, Developmental Disorder, Organismal Injury and Abnormalities
5	***CDH22***, ***COL1A2***, *DIRAS1*, *ELK3*, ***GUF1***, ***HRH4***, *NLRP12*, ***RAMP2***, *RNF180*, *RNF187*, ***RXRB***, *SSTR3*, ***TNFRSF4***, *TPMT*	1.00 × 10^−24^	14	Developmental Disorder, Hereditary Disorder, and Neurological Disease
6 *	***CACNB1***, ***DUSP26***, *IFFO1*, *LMCD1*, *MRGPRX3*, *OR7G1*, *RAB43*, *RHCE*, *RHD*, ***SNX7***, ***TBC1D21***, *TMEM80*, *WBP2NL*, *ZSCAN20*	1.00 × 10^−22^	14	Cellular Compromise, Hypersensitivity Response, and Inflammatory Response
7 *	*C11orf16*, *CAPN13*, *CCDC151*, *CLCA4*, *DEAF1*, ***FBXL20***, *IQCF5*, ***MAPK1IP1L***, *MRGPRX4*, *NAGA*, ***SHISA7***, *SMDT1*, *TMEM225*	1.00 × 10^−22^	13	Cell Cycle, Glomerular Injury, Organismal Injury, and Abnormalities
8 *	*ARHGAP24*, *FBXO33*, *OR7G2*, *OR7G3*, ***PDXP***, *PYGO2*, *SERTAD4*, ***SLC4A4***, ***STAC2***, *TRIM17*, *VPS25*, *XKR4*	1.00 × 10^−20^	12	Amino Acid Metabolism, Drug Metabolism, Endocrine System Development and Function
9 *	*CCDC66*, *NUBP1*, *PDDC1*, *RAPH1*, *SLC39A3*, *TVP23A*	1.00 × 10^−8^	6	Digestive System Development and Function, Gastrointestinal Disease, and Hepatic System Development and Function

**Table 3:** Firstly, we conducted PRS-based analyses, with three GWASs of AD as base sample (in bold) and four GWASs of UUI as target sample. The *p* values that remained significant after Bonferroni correction for all PRS-based analyses (=12) were indicated with †. Secondly, we conducted PRS-based analyses, with one GWAS of the Aβ42/Aβ40 blood level ratio as base sample (in bold) and three GWASs of UUI as target sample. The *p* values that remained significant after Bonferroni correction for all PRS-based analyses (=3) were indicated with †.

FinnGen	Best *p*T	N SNPs	*p* Value	Variance Explained R^2^
Richter et al. (update) [15]	0.001	1918	3.96 × 10^−3^	0.0022438348
Penney et al. [16]	0.3	146,587	1.15 × 10^−3^	0.0013751369
Cartwright et al. [17]	0.05	55,274	3.10 × 10^−3^	0.0008344947
HUNT	0.1	169,806	2.21 × 10^−1^	0.0000319658
**Jansen et al.** [238]				
Richter et al. (update) [15]	0.5	445,721	3.45 × 10^−1^	0.0000503918
Penney et al. [16]	0.5	248,600	5.98 × 10^−3^	0.0009349944
Cartwright et al. [17]	0.4	367,515	4.35 × 10^−3^	0.0007669877
HUNT	0.001	3472	3.37 × 10^−3^	0.0003959924
**Bellenguez et al.** [236]				
Richter et al. (update) [15]	0.2	228,958	9.55 × 10^−2^	0.0005443668
Penney et al. [16]	0.4	198,042	2.13 × 10^−2^	0.0006091996
Cartwright et al. [17]	0.05	70,273	4.85 × 10^−8^ †	0.0031641935
HUNT	0.05	121,328	9.93 × 10^−2^	0.0000891644
**Damotte et al.** [237]	**Best *p*T**	**N SNPs**	***p* Value**	**Variance Explained R^2^**
Penney et al. [16]	0.001	1247	5.29 × 10^−2^	0.000387
Cartwright et al. [17]	0.1	108,908	2.31 × 10^−3^ †	0.000894
HUNT	0.5	588,577	1.62 × 10^−1^	0.000079

**Abbreviations:** Aβ, amyloid beta; AD, Alzheimer’s disease; GWAS, genome-wide association study; PRS, polygenic risk score; *p*T, *p* value threshold; SNPs, single nucleotide polymorphisms; UUI, urgency urinary incontinence.

**Table 4:** Characteristics of the genome-wide association studies (GWASs).

GWAS Dataset	Population	UUI Patients	Controls	Diagnosis	Definition of UUI	Definition of Controls	Adjusted for Confounding Factors
Richter et al. (update) [15]	USA	2322	817	Self-reported via questionnaire	UUI more than once a month and who leaked sufficiently to wet or soak their underpants or clothes	No UUI	PCs, age, obesity (BMI ≥ 30), diabetes, and parity
Penney et al. [16]	USA	1942	4811	Self-reported on (seven) biennial questionnaires	At least weekly leakage of urine and most leaking episodes related to a feeling of urgency	Never or no more than leaking a few drops less than once a month on all questionnaires	PCs, age, BMI, parity, and type II diabetes
Cartwright et al. [17]	European	870	8127	Self-reported via questionnaire	Self-reported ‘UUI’, leakage of urine when ‘rushing to the toilet’	No UUI	PCs, age, BMI, and parity
HUNT	European	693	23,055	Self-reported via questionnaire	Urgency urinary leakage ≥1×/month (no SUI)	No UUI	Birth year, BMI, and parity

**Abbreviations:** BMI body mass index, ICD-10 International Classification of Diseases version 10, PCs principal component analysis, SUI stress urinary incontinence, UUI urgency urinary incontinence.

## Data Availability

All key data that support the findings of this study are available in the main text or the Supplementary Materials. Other, more extensive data are available from the corresponding author upon reasonable request.

## Supplementary Materials

The following supporting information can be downloaded at: https://www.mdpi.com/article/10.3390/ijms27146156/s1.

## Abbreviations

The following abbreviations are used in this manuscript:

AD	Alzheimer’s disease
AICD	APP intracellular domain
APP	Amyloid Precursor Protein
ATP	Adenosine triphosphate
Aβ	Amyloid beta
CNS	Central nervous system
eQTL	expression quantitative trait loci
ESR1	Estrogen receptor 1
ESR2	Estrogen receptor 2
GARNET	Women’s Health Initiative Genomics & Randomized Trials Network
GWAS	Genome-wide association study
HUNT	Trøndelag Health Study
IPA	Ingenuity pathway analysis
kb	kilobase
OAB	Overactive bladder syndrome
PRS	Polygenic risk score
SNP	Single nucleotide polymorphism
UUI	Urgency urinary incontinence
