# Supplementary material for: Amyloid Precursor Protein Processing Links Female Urgency Urinary Incontinence with Alzheimer’s Disease: Implications for Treatment
Source: Int J Mol Sci. 2026 Jul 9;27(14):6156. doi: 10.3390/ijms27146156 (PMC13409906; doi:10.3390/ijms27146156)
Supplement: Supplementary file 1 [file ijms-27-06156-s001.zip › ijms-4327972-supplementary.pdf]

Supplementary Information

- Supplementary Tables S1-S4
- Supplementary Text S1 - Description of molecular landscape of urgency urinary incontinence in women, with Supplementary Figures S2a, S2b and S2c (representing urothelial cells, a neuron and muscle cells in the landscape)
- Supplementary Text S2 - Methodology of GWASs
- Supplementary Text S3 - Detailed description of methodology for PRS-based analyses
- Supplementary Figure S1 - Radial presentation of the six merged, overlapping networks resulting from the network enrichment analysis
- Supplementary Figure S3 - Bar plots from PRSice showing results for shared genetic etiology between AD and UUI
- Supplementary Figure S4 - Bar plots from PRSice showing results for shared genetic etiology between the Aβ42/Aβ40 blood level ratio and UUI

Supplementary Table S1. Twelve landscape genes/proteins implicated in UUI through evidence other than GWAS (underlined in this table and green in the molecular landscape in Fig 1) and 15 genes/proteins that have not been implicated in UUI (yet) but interact with at least two landscape proteins (white in the molecular landscape in Fig 1). The 12 genes that show nominally significant evidence of association with UUI (gene-wide p<0.05) in one of the four GWASs are designated with *

	**Best p values**				
**Additional genes**	**Richter et al. (update)** [1]	**Penney et al.** [2]	**Cartwright et al.** [3]	**HUNT**	**Corroborating evidence**
*ADM**	6.56E-02	1.74E-01	3.84E-02	4.28E-01	- Adrenomedullin levels were increased in children with detrusor instability compared to controls [4].
APOB	6.85E-02	2.42E-01	6.97E-01	2.26E-01	- APOB is a major protein constituent of chylomicrons, LDL, and VLDL [5]. Increased urinary levels of LDL are predictive factors for overactive bladder [6].
APP	4.99E-01	8.42E-01	5.17E-01	1.91E-01	-
AR	NA	NA	NA	NA	- Genetic variants in the AR gene have been found in patients with UUI [7].
*CALHM1**	9.26E-02	2.94E-01	2.55E-03	5.29E-02	- CALHM1 is involved in the release of ATP by urothelial cells in reaction to bladder distention [8] and increased ATP release is associated with.UUI [9-11].
CAT	1.81E-01	1.49E-01	4.22E-01	3.39E-01	- Antioxidant enzyme catalase is a potential treatment option in preventing detrusor overactivity after H2O2 induction in rats [12].
CD40L	NA	NA	NA	NA	- CD40L is increased in the urine of patients with overactive bladder syndrome [13].
*CDH1**	3.41E-01	6.19E-01	3.75E-01	3.02E-02	-
*CEBPZ**	2.54E-01	3.49E-03	1.98E-03	3.38E-01	-
ESR1	9.74E-01	5.96E-01	8.60E-02	2.69E-01	- Estrogen therapy can be useful in relieving overactive bladder symptoms [14, 15].<br>- Estrogen deficiency may be associated with UUI [16].<br>- Ovariectomy in animal models induces detrusor overactivity [17, 18].<br>- Bilateral oophorectomy is associated with a higher prevalence of OAB [19].
ESR2	4.18E-01	2.95E-01	9.76E-01	7.09E-01	- Estrogen therapy can be useful in relieving overactive bladder symptoms [14, 15].<br>- Estrogen deficiency may be associated with UUI [16].<br>- Ovariectomy in animal models induces detrusor overactivity [17, 18].<br>- Bilateral oophorectomy is associated with a higher prevalence of OAB [19].
EZH2	1.33E-01	7.94E-01	2.18E-01	9.63E-01	-
*HECW2**	7.25E-01	3.34E-01	2.82E-03	1.63E-01	-
*IL2RB**	9.66E-01	3.02E-01	1.05E-03	9.03E-01	-

IL10	3.18E-01	1.04E-01	1.31E-01	8.16E-01	- Urinary IL-10 is a potential biomarker for patients with OAB [13, 20].
*ITGA2**	3.46E-01	2.42E-01	4.33E-03	4.46E-01	-
ITGB1	2.88E-01	7.55E-02	9.85E-02	4.43E-01	- Conditional ITGB1 knockout in the urothelium of mice resulted in OAB [21].
MAPK8	4.50E-01	5.43E-01	6.30E-01	9.34E-01	-
*MYC**	3.05E-01	1.04E-01	4.63E-03	4.33E-01	-
*NEDD4L**	8.79E-02	1.73E-02	7.21E-01	1.22E-01	-
*NGF**	8.87E-01	2.31E-01	4.07E-02	3.85E-01	- Increased urinary levels of NGF are associated with UUI [22].
*NUDCD3**	9.06E-01	2.69E-02	1.51E-03	4.92E-01	-
RBFOX2	2.23E-01	4.45E-01	2.68E-01	8.36E-01	-
RYR1	1.27E-01	4.80E-01	4.58E-01	7.48E-01	- A lower expression of RYR1 is associated with detrusor overactivity in female rat [23].
*SAA1**	1.62E-01	3.78E-01	1.24E-03	5.76E-01	-
SUMO1	6.27E-01	2.07E-01	1.86E-01	4.32E-01	-
TP73	3.82E-01	4.83E-01	6.67E-02	5.06E-02	-
**Abbreviations:** ATP adenosine triphosphate, GWAS genome-wide association study, LDL low-density-lipoprotein, OAB overactive bladder, UUI urgency urinary incontinence, VLDL very-low-density-lipoprotein.					

**Supplementary Table S2.** Nine additional landscape molecules that have been associated with UUI (green in the molecular landscape in Fig 1 and underlined in this table) and one additional molecule that has not yet been associated with UUI (white in the molecular landscape in Fig 1).	
**Additional molecules**	**Corroborating evidence**
ATP	- Increased ATP release is associated with UUI [9-11].
Bicarbonate (HCO3-)	- Oral administration of sodium bicarbonate is a promising treatment for UUI [24, 25].
Calcium (Ca2+)	- Calcium antagonists are potential treatment options for overactive bladder [10].
Chondroitin sulfate (cs)	- Chondroitin sulfate is a promising therapeutic option for overactive bladder [26].<br>- Chondroitin sulfate reduces pelvic pain and urgency symptoms [27].
Dihydrotestosterone (DHT)	-
Estradiol (E)	- Estrogen therapy can be useful in relieving overactive bladder symptoms [14, 15].<br>- Estrogen deficiency may be associated with UUI [16].
Glutamate (GL)	- Glumateric signaling in afferent neurons is involved in regulating normal bladder function [28].<br>- Glutamate is associated with bladder overactivity in cats [29, 30].
IgE	- There is a clinical correlation between allergy and IgE plasma levels and OAB [31, 32].
Retinoic acid (RA)	- Retinoic acid induces bladder overactivity in rats [33].
**Abbreviations:** ATP adenosine triphosphate, OAB overactive bladder, UUI urgency urinary incontinence.	

Supplementary Table S3. PRS-based analyses, with three GWASs of AD as base sample (in bold) and four GWASs of UUI as target sample. The p values that remained significant after Bonferroni correction for all GWASs (=12 analyses) were indicated with †.

**FinnGen**	**Threshold**	**N SNPs**	**p value**	**Variance explained R^2**
Richter et al. (update) [1]	0.001	1918	3.96E-03	0.0022438348
	0.05	61053	1.71E-01	0.0002864972
	0.1	112192	3.53E-01	0.0000452216
	0.2	202890	1.48E-01	0.0003488119
	0.3	283775	1.97E-01	0.0002309224
	0.4	356299	2.59E-01	0.0001335462
	0.5	422257	3.29E-01	0.0000621587
Penney et al. [2]	0.001	1184	8.21E-02	0.0013751369
	0.05	33109	4.39E-02	0.0010264556
	0.1	59526	5.31E-03	0.0008519211
	0.2	105792	6.19E-03	0.0005204681
	0.3	146587	1.02E-03	0.0008344947
	0.4	182304	4.23E-03	0.0002982451
	0.5	214574	8.22E-03	0.0000521351
Cartwright et al. [3]	0.001	1767	1.53E-02	0.0000482350
	0.05	55274	3.10E-03	0.0000370998
	0.1	101062	5.09E-02	0.0000217439
	0.2	182510	2.47E-01	0.0000042845
	0.3	255474	2.55E-01	0.0000010813
	0.4	319898	2.82E-01	0.0000319658
	0.5	378262	3.29E-01	0.0000000422
HUNT	0.001	2605	3.89E-01	0.0000000713
	0.05	91255	4.44E-01	0.0000032333
	0.1	169806	2.21E-01	0.0000000060
	0.2	311335	4.89E-01	0.0002309224
	0.3	439439	4.86E-01	0.0001335462
	0.4	552446	4.03E-01	0.0000621587
	0.5	655995	4.96E-01	0.0002863860
**Jansen et al. [34]**				
Richter et al. (update) [1]	0.001	2953	4.18E-01	0.0000137099
	0.05	77459	4.07E-01	0.0000174787
	0.1	136619	4.98E-01	0.0000000105
	0.2	234583	3.94E-01	0.0000228895
	0.3	316469	4.61E-01	0.0000030669
	0.4	385783	3.52E-01	0.0000458524
	0.5	445721	3.45E-01	0.0000503918
Penney et al. [2]	0.001	2026	2.93E-02	0.0005296980
	0.05	45861	2.27E-01	0.0000828378
	0.1	79229	2.32E-01	0.0000794772
	0.2	133791	2.06E-02	0.0006172647
	0.3	178608	1.50E-02	0.0006967927

	0.4	216273	8.22E-03	0.0008518357
	0.5	248600	5.98E-03	0.0009349944
Cartwright et al. [3]	0.001	2904	4.11E-02	0.0003365459
	0.05	73500	2.36E-02	0.0004389115
	0.1	129563	1.79E-02	0.0004915800
	0.2	222678	1.67E-02	0.0005047189
	0.3	300823	1.26E-02	0.0005584456
	0.4	367515	4.35E-03	0.0007669877
	0.5	425081	4.72E-03	0.0007506676
HUNT	0.001	3472	3.37E-03	0.0003959924
	0.05	95766	9.11E-03	0.0003006559
	0.1	171538	5.73E-02	0.0001343196
	0.2	300070	1.02E-01	0.0000872845
	0.3	409317	3.17E-02	0.0001859532
	0.4	503721	1.86E-02	0.0002339557
	0.5	586146	2.50E-02	0.0002072869
**Bellenguez et al. [35]**				
Richter et al. (update) [1]	0.001	4071	1.57E-01	0.0003234832
	0.05	76644	3.65E-01	0.0000377671
	0.1	132500	2.96E-01	0.0000917705
	0.2	228958	9.55E-02	0.0005443668
	0.3	312829	1.56E-01	0.0003261650
	0.4	387114	2.59E-01	0.0001336875
	0.5	452870	2.08E-01	0.0002104715
Penney et al. [2]	0.001	2713	2.74E-01	0.0000536633
	0.05	42277	9.89E-02	0.0002454905
	0.1	71257	1.24E-01	0.0001979174
	0.2	120053	5.51E-02	0.0003778222
	0.3	161539	3.91E-02	0.0004593835
	0.3	198042	2.13E-02	0.0006091996
	0.4	229534	3.28E-02	0.0005014789
	0.5	3816	2.30E-03	0.0008948853
Cartwright et al. [3]	0.001	70273	4.85E-08†	0.0031641935
	0.05	121302	5.05E-06†	0.0021705109
	0.1	208758	2.77E-05†	0.0018105478
	0.2	284186	1.39E-04†	0.0014710537
	0.3	350634	6.21E-05†	0.0016403010
	0.3	409209	9.69E-05†	0.0015472464
	0.4	5378	1.89E-01	0.0000418214
	0.5	121328	9.93E-02	0.0000891644
HUNT	0.001	214430	1.11E-01	0.0000807478
	0.05	375315	2.66E-01	0.0000210664
	0.1	514498	2.71E-01	0.0000200496
	0.2	637036	1.96E-01	0.0000394469
	0.3	745573	3.20E-01	0.0000117747

0.3	4071	1.57E-01	0.0003234832
0.4	76644	3.65E-01	0.0000377671
0.5	132500	2.96E-01	0.0000917705

Abbreviations: AD Alzheimer's disease, GWAS genome-wide association study, PRS polygenic risk score, SNP single-nucleotide polymorphism.

Supplementary Table S4. PRS-based analyses, with one GWAS of the Aβ42/Aβ40 blood level ratio as base sample (in bold) and three GWASs of UUI as target sample. The p values that remained significant after Bonferroni correction for all GWASs (=3 analyses) were indicated with †.

**Damotte et al. [36]**	**Threshold**	**N SNPs**	**p value**	**Variance Explained R^2**
Penney et al. [2]	0.001	1247	5,29E-02	0,000387
	0.05	40012	4,18E-01	6,38E-06
	0.1	71527	2,00E-01	0,000105
	0.2	125003	1,55E-01	0,000153
	0.3	170524	2,06E-01	9,94E-05
	0.4	210078	2,89E-01	4,57E-05
	0.5	244387	2,33E-01	7,9E-05
Cartwright et al. [3]	0.001	1762	2,69E-01	4,22E-05
	0.05	59674	8,07E-03	0,000645
	0.1	108908	2,31E-03 †	0,000894
	0.2	196421	7,10E-03	0,00067
	0.3	274344	7,23E-03	0,000666
	0.4	343804	1,84E-02	0,000486
	0.5	405332	2,70E-02	0,000414
HUNT	0.001	1882	3,83E-01	7,19E-06
	0.05	77148	1,93E-01	6,08E-05
	0.1	145249	1,96E-01	5,92E-05
	0.2	273059	2,92E-01	2,42E-05
	0.3	390066	1,99E-01	5,76E-05
	0.4	494234	1,88E-01	6,33E-05
	0.5	588577	1,62E-01	7,86E-05

Supplementary text S1 - Description of molecular landscape of urgency urinary incontinence in women

Introduction

This molecular landscape is illustrative for multiple areas in the female lower urinary tract that are all associated with (the development of) UUI [37]. This description provides an overview of all protein-protein interactions that are drawn in and surrounding the different cell types in the molecular landscape, i.e., urothelial cells, muscle cells, and neurons. Although the bladder morphology is (much) more complex and many other cell types (including muscle cells, neurons, and fibroblasts) are involved, urothelial cells are the key players in many bladder signaling pathways [38]. The urothelial cells that are shown in Fig 1 represent cells in all layers of the urothelium, and in both the bladder and the urethra. The neuron that is shown represents both afferent and efferent neurons. Further, the extracellular matrix (ECM) represents the area surrounding both the bladder and urethra and the 'lumen' refers to both the bladder and urethral lumen, filled with urine. In addition, some of the described and drawn proteins and protein-protein interactions are cell type-specific while others are more generic. In this respect, the main location of each interaction was based on its links with other protein-protein interactions that, in turn, may be cell type-specific or most representative based on hypotheses about the pathophysiology of UUI. Furthermore, in Supplementary Figures 2a, 2b and 2c below, the specific interactions in and around urothelial cells, a neuron and muscle cells are shown, respectively

In the detailed description below, the names of proteins derived from the genome-wide association studies (GWASs) of UUI are indicated in bold and these proteins are blue. All genes/proteins and molecules that have been implicated in UUI through other genetic evidence (from e.g., candidate gene association studies), functional evidence (from e.g., animal studies) and/or that are differentially expressed in UUI (tissues) are underlined and green. Proteins and molecules that have not been linked to UUI (yet) are not bold or underlined in the description, and they are white in the landscape. In addition, GWAS-derived proteins are only shown in the landscape if they interact with other GWAS-derived proteins and/or other UUI-implicated proteins, if they have also been implicated in UUI through evidence other than GWAS, or both. An overview of all green and white landscape proteins and molecules - with the evidence implicating them in UUI - is provided in Supplementary Tables 1 and 2, while the blue proteins encoded by the top GWAS genes are shown in Table 2.

Overall, two assumptions have been made. First, when knock-out of gene/protein A in a cell or animal model increases the expression of gene/protein B, we assume that endogenous expression of gene/protein A leads to the opposite effect and decreases the expression of gene/protein B. Second, we assumed that all identified protein interactions (in any organism and/or cell type) can be extrapolated to the interactions in all types of human cells, and specifically the cell types in our landscape. Moreover, the abbreviations used for the gene name refer to both the gene and protein. When using the terms ‘activates’ or ‘inhibits’, this indicates the activation or inhibition of a specific protein by another protein, e.g., by (de)phosphorylation. Furthermore, when a difference in abundance of a specific mRNA/protein (directly or indirectly) is induced by another protein, the terms ‘increases/upregulates the expression’ or

‘decreases/downregulates the expression’ are used. Other possible interactions are 'is transported by/transports', 'degrades/cleaves', 'binds and (functionally) interacts with/forms a (functional) complex with', and 'produces'. Finally, some interactions were not drawn in the molecular landscape of UUI, mostly because these interactions only and specifically occur in other cell types. These are indicated with ‘(not shown)’ in the text.

The key process in the molecular landscape of UUI is APP signaling. APP or amyloid precursor protein is a transmembrane protein that is involved in neurite growth, neuronal adhesion and axonogenesis [5, 39]. Further, APP has been associated with cell mobility and - through the APP intracellular domain or AICD - transcriptional regulation [5, 40-42]. The peptide amyloid beta (Aβ) is produced (and released in the extracellular space) through cleavage of APP by β- and γ-secretases. In this respect, an accumulation and clustering of extracellular Aβ plaques in the brain is one of the key pathological features of (the development of) Alzheimer’s disease (AD) [43]

Urothelial cells

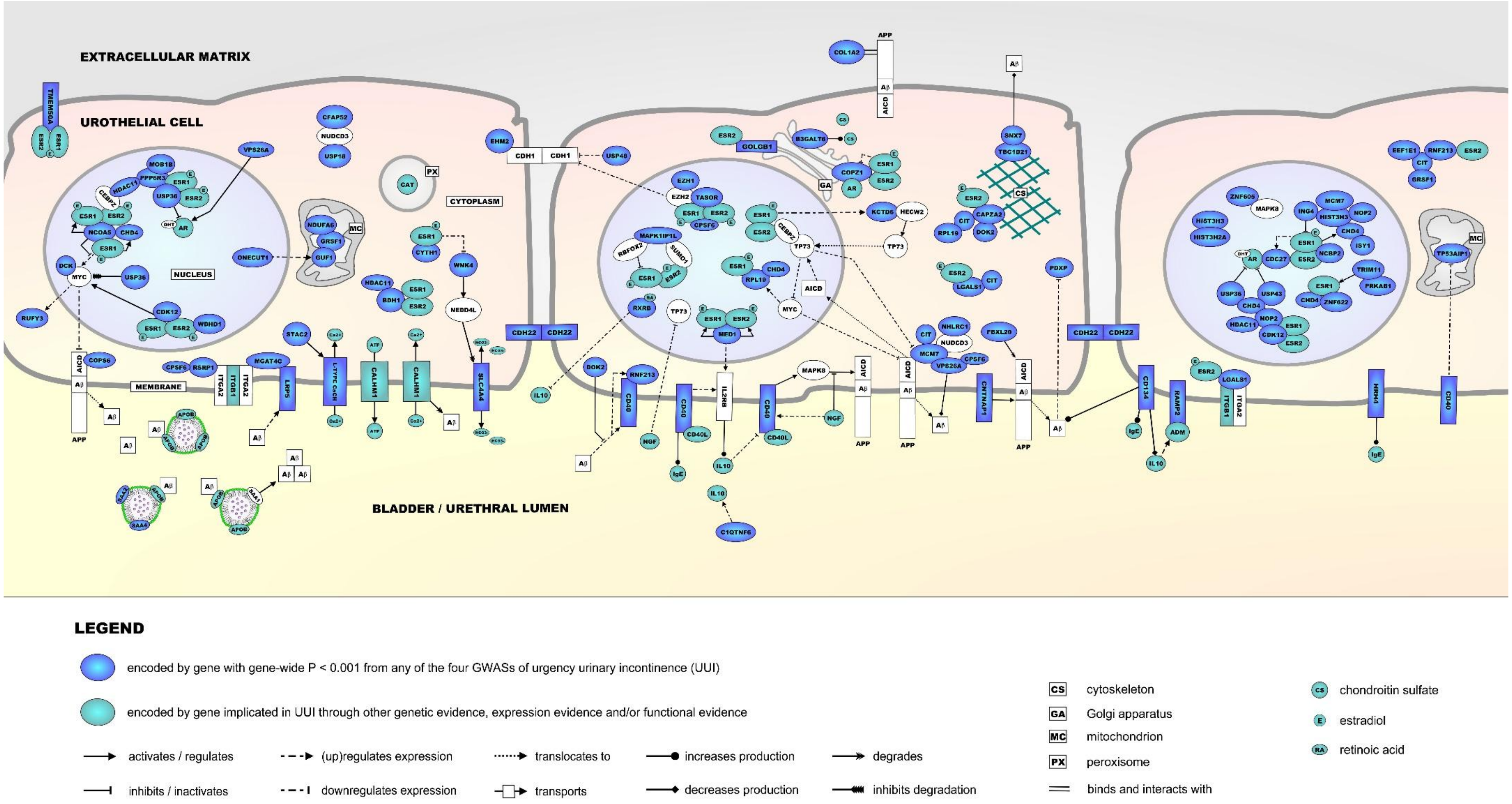

Supplementary Figure S2a. Specific landscape interactions in and around urothelial cells.

Nucleus

Following the cleavage of APP, AICD translocates to the nucleus, where it functions as a transcription factor [44, 45] and is involved in DNA repair and apoptosis [45]. For example, AICD upregulates the expression of TP73 [46], a protein with multiple functions in the landscape (see below). In addition to APP signaling, estrogen receptor signaling plays an important role in the molecular landscape. Estrogens are sex steroid hormones that are involved in the regulation of the menstrual cycle, reproduction, bone density, brain function, cholesterol mobilization, development of breast tissue and sexual organs, and in controlling inflammation [47]. ESR1 and ESR2 are predominantly nuclear hormone receptors that, when bound by the female sex hormone estradiol [47-49], regulate the expression of multiple genes. ESR1 and ESR2 bind each other and form a transcription factor complex [50, 51]. Further, this complex regulates cellular proliferation and differentiation [5].

In the nucleus of urothelial cells, the estradiol-bound ESR1-ESR2 complex is bound and activated/regulated by **MED1** [52-55] and **NCOA5** [56]. **MED1** acts a transcriptional regulator [5] that upregulates the expression of IL2RB [57] (see below) and is involved in translocating CDH1 from the cytoplasm to the membrane [58] (not shown). **NCOA5** is a nuclear receptor and transcriptional coregulator that inhibits the ESR1-induced upregulation of MYC expression [59] (see below). In addition to the ESR1-ESR2 complex [56], **NCOA5** forms a functional complex with **CHD4** [60].

Moreover, ESR1 is cleaved and hence stabilized by the ubiquitin-protein ligase **TRIM11** [61]. **TRIM11** also complexes with **PRKAB1** [62], a protein that regulates the cellular energy metabolism [5]. In addition, ESR1 (function) is negatively regulated by **ING4** [63] (a protein involved in cell proliferation [5]) and by the RNA splicing factor RBFOX2 [5, 64]. RBFOX2 also forms a functional complex with **MAPK1IP1L** [65], a protein of unknown function that is nevertheless detected in the human urine [66] and also complexes with SUMO1 [65], an interactor of ESR2 (see below). Furthermore, activated ESR1 increases the expression of **CDC27** [67], **KCTD6** [68] - a cytoplasmic ubiquitination protein [5] -, and MYC [59], and it regulates the activity of CDH1 [69] (not shown) and **CHD4** [70]. **CDC27** is a ubiquitin ligase [5, 71] that also binds and interacts with the androgen receptor (AR) [72] and ESR2 [73] (see below). Interestingly, genetic variants in the AR gene have been found in patients with UUI [7]. MYC is a transcription factor [74] that upregulates the expression of the RNA-binding protein **RPL19** [5, 75] and regulates the splicing (not shown) and expression of **RUFY3** [76], a protein that is involved in cytoskeletal organization and neuronal development [5] and that is upregulated in the olfactory bulb of AD patients [77]. In addition, MYC downregulates the expression of APP [78] and complexes with the nuclear kinase **DCK** [5, 79]. **CHD4** is involved in epigenetic transcriptional repression [5], highly expressed in the urothelium [80] and, apart from its abovementioned complex with **NCOA5** [60], it binds and functionally interacts with ESR1 [81], histone **HIST3H3** [82] (see also below), the component of the pre-mRNA spliceosome **ISY1** [5, 83], as well as the nuclear proteins **NOP2** [60], **RPL19** [60], **USP36** [60, 84], **USP43** [85], and the transcription factor **ZNF622** [60]. Moreover, ESR1 binds and interacts with (nuclear) **CDC27** [86] (not shown), **CDK12** [86], **CPSF6** [86], EZH2 [86, 87], **NCBP2** [86], **NOP2** [88], **PPP6R3** [86], **RPL19** [89], **TASOR** [86], **USP36** [86], and **ZNF622**

[86]. As for ESR2, this protein (also) complexes with (nuclear) **CDC27** [73], **CDK12** [73, 90], CEBPZ [73], **CPSF6** [73], **NCBP2** [73], SUMO1 [73], **TASOR** [73], **USP36** [73], and the replication initiation factor [5] **WDHD1** [73]. **CDK12** is a key regulator of transcription elongation [5], is highly expressed in urothelial cells [80], activates MYC [91, 92] and (also) complexes with **NOP2** [62]. **CPSF6** is involved in RNA cleavage and processing and plays a role in mRNA export [5, 93]. EZH2 is a transcriptional repressor [5] that downregulates the expression of CDH1 [94] and forms a functional complex with the transcriptional repressor **EZH1** [95] and the epigenetic repressor **TASOR** [5, 96]. **NCBP2** is highly expressed in the urothelium [80] and is involved in RNA processing [5].

NOP2 is involved in RNA methylation [5] and, in addition to its prementioned complexes with **CDK12** [62], **CHD4** [60], and ESR1 [88] (see above), forms a complex with **HDAC11** [97], a histone deacetylase that modulates epigenetic transcriptional repression [5], and the epigenetic transcription regulator histone protein **HIST3H3** [5, 98]. **HIST3H3**, in turn, complexes with **CHD4** [82] (see above), **HIST3H2A** [99], **ING4** [100], and **MCM7** [101]. Further, **PPP6R3** - which is involved in protein phosphatase binding and maintaining immune self-tolerance [102, 103] - complexes with **HDAC11** [97], **MOB1B** [104], and **USP36** [84]. **MOB1B** is regulates skeletal muscle development and regeneration [105] (not shown). **USP36** is a deubiquitinase [5] that inhibits the degradation of MYC [106] and AR function [107].

CEBPZ is a transcriptional coactivator [5] that binds and interacts with **HDAC11** [97] and TP73 [108], a protein that regulates the response to DNA damage [109] and itself decreases the expression of **MCM7** [110] and MYC [110]. Lastly, SUMO1 is an ubiquitin-like protein involved in DNA repair [5].

In addition, the transcriptional activator **ONECUT1** [5] upregulates the expression of **GUF1** [111], a protein that is involved in mitochondrial protein synthesis [5] (see below). Further, when bound and activated by its agonist retinoic acid (RA), the nuclear receptor **RXRB** downregulates the expression of IL10 [112] and inhibits the secretion of immunoglobulin E (IgE) antibodies [112] (not shown). Interestingly, increased IgE blood/urine levels have been associated with the allergic response and have specifically been implicated in overactive bladder [13, 20, 31, 32]. Moreover, RA has been found to protect against AD disease and inhibit Aβ accumulation [113, 114], but it also induces bladder overactivity in rats [33]. Lastly, the ubiquitination protein **USP43** [5] inhibits AR [107] while the transcription factor **ZNF605** [5] binds and interacts with MAPK8 [115] (see below).

Cytoplasmic interactions related to ESR1 and ESR2
Apart from their important role in the nucleus, (estradiol-bound and -activated) ESR1 and ESR2 also function in the cytoplasm [47, 116]. ESR1 downregulates the expression of **COPZ1** [67] and **WNK4** [117]. Further, ESR1 complexes with **BDH1** [86], **COPZ1** [86], **CYTH1** [86] - a protein involved in membrane trafficking during junctional remodeling and epithelial repolarization [5] - and with the transmembrane protein of unknown function **TMEM50A** [86] (see below). **WNK4** is a protein kinase that regulates electrolyte homeostasis and cell survival/proliferation [5, 118].

In line with this, **WNK4** activates NEDD4L [118], a ubiquitin protein ligase [5] that itself mediates the degradation of **SLC4A4** [119]. Moreover, NEDD4L is upregulated in chronic bladder ischemia [120], a process that has been associated with (refractory) overactive bladder [121]. **SLC4A4** is a membrane transporter that mediates bicarbonate influx and efflux in order to regulate the intracellular pH [5], and in this way also the urinary pH [122]. In this respect, it is interesting that oral administration of sodium bicarbonate seems to improve UUI symptoms [24, 25]. Estradiol [123] and the male sex hormone dihydrotestosterone - the ligand of the AR - also decrease and increase [124] the expression of **SLC4A4**, respectively (not shown). **BDH1** is an enzyme that regulates lipid metabolism [5] and that has been associated with aging [125] and with the conversion of mild cognitive impairment to AD [126]. Moreover, **BDH1** (also) binds and interacts with ESR2 [73] and **HDAC11** [97].

COPZ1, a Golgi membrane protein that modulates Golgi-to-endoplasmic reticulum transport [127] also complexes with (cytoplasmic) AR [107] and ESR2 [73]. In addition to the abovementioned proteins, cytoplasmic ESR2 binds and interacts with **CAPZA2** [73], CDH1 [73] (not shown), **GOLGB1** [73], **LGALS1** [73], **RNF213** [73] (see below), and, together with ESR1 (see above), **TMEM50A** [73]. **CAPZA2** binds the actin filaments and is hence involved in organizing the actin cytoskeleton [5]. Further, it binds and interacts with the adaptor protein **DOK2** [128]. **GOLGB1** is an important regulator of Golgi apparatus function [5]. Lastly, **LGALS1** is involved in in regulating apoptosis and cell proliferation/differentiation [5], and it complexes with **CIT** [129] as well as the membrane-located integrin proteins ITGA2 [62] and ITGB1 [62] (which also form a functional complex) (see below).

Other cytoplasmic and cytoplasmic-membrane interactions

B3GALT6 is a Golgi membrane-located enzyme that metabolizes glycosaminoglycans such as chondroitin sulfate [130, 131]. In this respect, it is interesting that chondroitin sulfate is a promising novel therapeutic option for overactive bladder [26] and reduces pelvic pain and urgency symptoms [27]. Further, the peroxisomal antioxidant enzyme catalase (CAT) prevents detrusor overactivity after hydrogen peroxide induction in rats [12] and it was found to reduce Aβ toxicity in the brain [132]. Moreover, **CIT**, a kinase that - among other functions - is involved in the development of the central nervous system [5] is also highly expressed in the urothelium [80] and forms functional complexes with (cytoplasmic) **CAPZA2** [129], **EEF1E1** [129], **GRSF1** [129], **LGALS1** [129] (see above), **MCM7** [129], **RNF213** [129], and **RPL19** [129]. **EEF1E1** regulates the response to DNA damage [5]. **GRSF1** regulates the post-transcriptional gene expression of mitochondria [5] and forms a functional complex with **GUF1** [133] and **NDUFA6** [133] - a subunit of the mitochondrial membrane respiratory chain NADH dehydrogenase [5] (Complex I) - in the mitochondria. Furthermore, in addition to binding **CAPZA2** (see above), **DOK2** is involved in negatively regulating the Aβ-induced increase of **CD40** - see below – expression [134]. Apart from being upregulated by ESR1 (see above), **KCTD6** complexes with HECW2 [135], a ubiquitin-protein ligase that stabilizes and hence induces the nuclear translocation and transcriptional activity of TP73 [136].

NUDCD3 is involved in the binding of unfolded proteins [5] and is highly expressed in urothelial cells [80]. Further, it complexes with (cytoplasmic) **CFAP52** [137], **MCM7** [137], **NHLRC1** [137], and **USP18** [137].

CFAP52 regulates cell growth and survival [138] while **MCM7** is a component of the MCM complex, a replicative helicase involved in DNA replication and elongation [5]. Moreover, **MCM7** complexes with APP [139] and **VPS26A** [140]. **NHLRC1** is a ubiquitin-protein ligase involved in the clearance of toxic protein aggregates and misfolded proteins [5], whereas **USP18** negatively regulates the inflammatory response [141]. Further, protein of unknown function **RSRP1** binds and interacts with **CPSF6** [142] and ITGA2 [143]. ITGA2 forms functional heterodimers with ITGB1 [5] and complexes with the glycosyltransferase **MGAT4C** [5, 140]. Interestingly, a decrease of ITGB1 expression in the urothelium has been associated with UUI [21], and the ITGA2/ITGB1 heterodimer is involved in mediating Aβ deposition and neurotoxicity by/in human cortical primary neurons [144]. **SNX7** is a protein involved in endocytosis and intracellular trafficking [145] that was also found to decrease the production of Aβ by increasing the lysosomal degradation of APP [146]. In addition, **SNX7** complexes with **TBC1D21** [147], a GTPase with a role in cytoskeletal reorganization [148]. In addition to binding **MCM7** [140], **VPS26A**, a protein implicated in intracellular transport [5], is involved in decreasing the production of Aβ [149, 150]. Further, it increases the transcriptional activity of AR [107] and complexes with **CPSF6** [143].

Interactions in and around the cell membrane, converging on APP processing

Firstly, the cytoplasmic protein kinase MAPK8 - which is expressed in both the urothelium [151] and bladder smooth muscle cells [152] - regulates bladder inflammation [152] and it activates APP (in the membrane) [153, 154]. Further, the cytoplasmic ubiquitin-protein ligase **FBXL20** [5] promotes APP degradation and inhibition of APP endocytosis [155]. **FBXL20** also inhibits the secretion of Aβ [155], and, in keeping with this, a lower expression of **FBXL20** has been associated with AD progression [156]. Moreover, **CNTNAP1**, an important player in myelination [5], is involved increasing the production of Aβ [157]. Furthermore APP binds and interacts with intracellular (cytoplasmic) **COPS6** [158], a protein that is involved in the ubiquitin conjugation pathway [5], ITGB1 [159] (not shown), **MCM7** [139] (see above), and extracellular **COL1A2** [160]. **CD40** is a receptor for (extracellular/urinary) CD40L that is (also) expressed in urothelial cells [161] and urine [66] and that regulates the secretion of immunoglobulins [5]. Interestingly, CD40L levels were found to be increased in the urine of patients with overactive bladder syndrome [13]. Further, the extracellular/urinary growth factor and cytokine NGF [162] and IL10 [163] increase and decrease the expression of **CD40**, respectively. Moreover, **CD40** upregulates IL2RB [164], downregulates the mitochondrial mediator of apoptosis **TP53AIP1** [5, 165], and activates MAPK8 [166, 167]. **CD40** also increases the production of IgE [168] and complexes with **RNF213** [62]. In addition, Aβ is involved in upregulating the expression of **CD40** [169] - which is inhibited by **DOK2**, see above - and **RNF213** [169], an atypical ubiquitin ligase involved in lipid metabolism [5]. Further, NGF inhibits the phosphorylation of APP [170] and decreases the expression of TP73 [171]. Increased urinary levels of NGF have also been associated with UUI [22], whereas the interleukin membrane receptor IL2RB increases the production of IL10 [172].

Moreover, **CACNB1** is a regulatory subunit of **L-type calcium channels** (transporting calcium (Ca^{2+}) into urothelial cells) [173, 174]. These channels are located in both the urothelium [175] and the detrusor muscle [10] (see below). The cytoplasmic **STAC2** slows down the inactivation rate of these channels [176, 177].

Moreover, modulators (agonists) of **L-type Ca2+ channels** [178, 179] and Ca2+ antagonists [10] are potential treatment options for overactive bladder. In addition, the membrane channel CALHM1 (also) transports Ca2+ into (urothelial) cells and it regulates the release of ATP by urothelial cells in reaction to bladder distention [8]. Further, increased ATP release has been associated with UUI [9-11] and it is produced by both the bladder urothelium and detrusor muscle [8, 10]. Importantly, CALHM1 is also involved in controlling APP cleavage and Aβ levels in a Ca2+-dependent manner [180]. Lastly, **CD134** is an immunity-related membrane receptor [5] that increases the production of IgE [181] and Aβ [182], while it decreases the production of IL10 [183].

Interactions involving urinary Aβ

Aβ is involved in regulating the expression of **LRP5** [184] and in downregulating **PDXP** expression [185]. **LRP5** is a membrane receptor that complexes with **MGAT4C** [140] (see above). Further, the cytoplasmic phosphatase **PDXP** [5] activates cofilin proteins [186] (not shown) that are affected by Rho kinase inhibitors that have been suggested as a novel treatment of overactive bladder [187]. In addition, serum amyloid A (SAA) is a biomarker for overactive bladder [188].

In addition, in chronic inflammatory conditions such as metabolic syndrome and diabetes mellitus, elevated (blood) levels of SAA have been found [189], and these conditions are also associated with overactive bladder/UUI [37, 121]. SAA proteins such as SAA1, **SAA2** and **SAA4** are located on apolipoprotein B (APOB)-containing lipoproteins [189, 190], e.g., LDL particles [5], and increased (urinary) levels of APOB-containing LDL particles have been linked to overactive bladder [6, 191, 192]. Moreover and importantly, Aβ binds and interacts with APOB [193] and SAA1 increases the aggregation of Aβ [194].

Other membrane-linked and urinary interactions

The Ca2+-dependent cell adhesion proteins Cadherin-1 (CDH1) and **CDH22** bind and interact with themselves, forming tight junctions between urothelial cells [5]. Further, **EHM2**, an epithelial cytoplasmic protein that promotes cellular adhesion, migration and motility [195], complexes with CHD1 [196], while the ubiquitination-linked protein **USP48** [197] is involved in downregulating CDH1 expression [198]. Moreover, the membrane histamine receptor **HRH4** is highly expressed in the urothelium [80] and mediates allergic inflammation through increasing the production of IgE [199]. **RAMP2** is a receptor for adrenomedullin (ADM), with increased ADM levels having been linked to detrusor instability [4]. ADM is expressed by both the urothelium and bladder smooth muscle [200], and IL10 upregulates the expression of ADM [201]. Lastly, **C1QTNF6** is a protein of unknown function that increases the production of IL10 [202].

Neuron

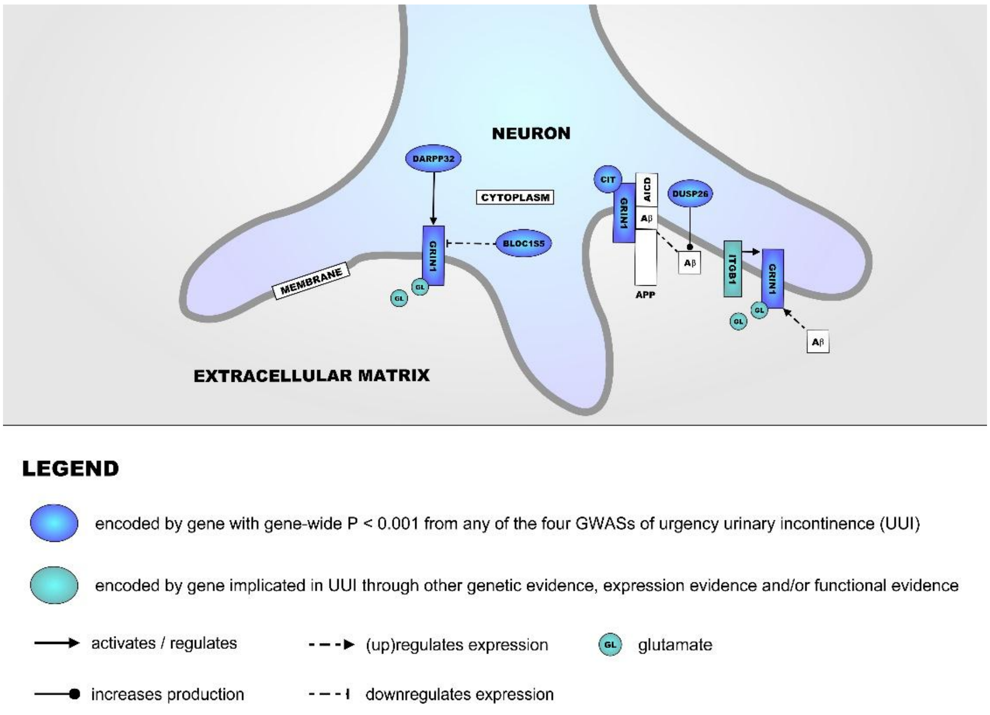

Supplementary Figure S2b. Specific landscape interactions in and around a neuron.

For the bladder and urethra to properly function, a complex interplay between afferent and efferent neurons that interact with both (detrusor) muscle cells and more directly with urothelial cells is required [203]. In this respect, cytoplasmic **DARPP32** - a protein that is highly expressed and has multiple functions in neurons [204] - activates the membrane glutamate (GL) receptor **GRIN1** [205]. Further, **BLOC1S5** - a component of the cytoplasmic that is involved in neurite extension and intracellular vesicle trafficking [5], - decreases the expression of **GRIN1** [206]. **GRIN1** is itself a component of NMDA glutamate receptor complexes that are expressed in the lower urinary tract [207]. Moreover, GL signaling involving afferent neurons is required for regulating normal bladder function [28] and (increased) GL signaling has been associated with bladder overactivity in cats [29, 30]. Inhibition of **GRIN1** was also found to reduce (cystitis-induced) bladder overactivity in rats [208]. In addition, Aβ regulates the expression of **GRIN1** [209, 210], while ESR1 decreases its expression [211] (not shown). ITGB1 - which also has multiple functions in urothelial cells, see above - activates **GRIN1** [212], and **GRIN1** forms a functional complex with APP [213] and **CIT** (the cytoplasmic kinase that also has multiple interactions with other landscape proteins in urothelial cells, see above) [214]. Lastly, the cytoplasmic protein phosphatase **DUSP26** [5] stimulates the generation of the pathological Aβ42 by promoting amyloid precursor protein axonal transport during hypoxia [215].

Muscle cells

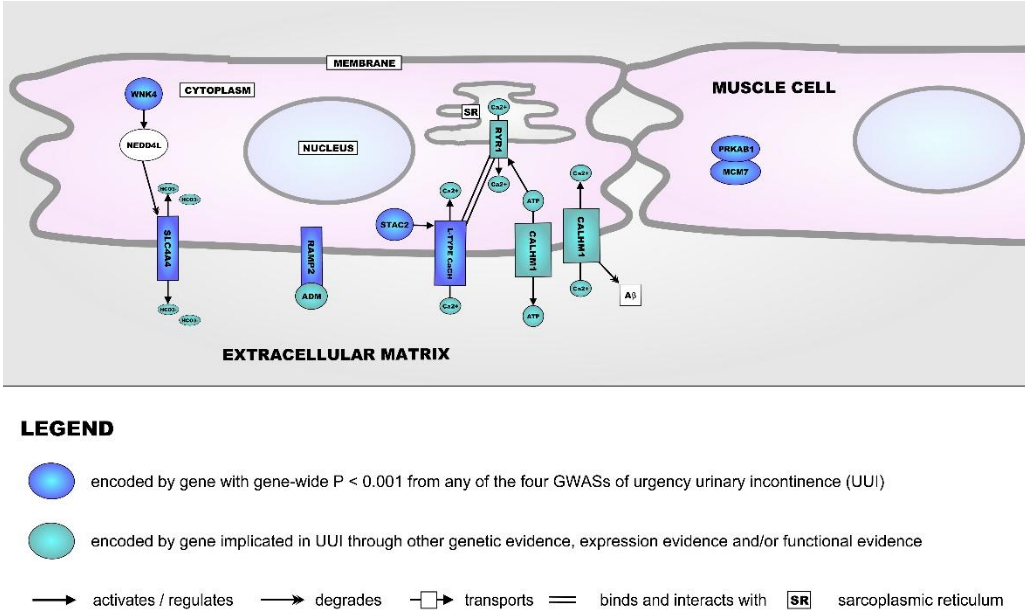

Supplementary Figure S2c. Specific landscape interactions in and around muscle cells.

In muscle cells, **PRKAB1** - a subunit of the cytoplasmic AMP-activated protein kinase (AMPK) [5] - forms a functional complex with **MCM7** [216] which, in addition to interacting with several landscape proteins in urothelial cells (see above), is a regulator of (smooth) muscle function [217]. Furthermore and as already indicated above, **L-type Ca2+ channels** - of which **CACNB1** is a regulatory subunit - transport Ca2+ into cells [173, 174] and are located in the membranes of both urothelial [175] and detrusor muscle cells [10]. Like in urothelial cells, cytoplasmic **STAC2** slows down the inactivation rate of these channels in muscle cells [176, 177]. Moreover, as already pointed out, modulators (agonists) of **L-type calcium channels** [178, 179] and Ca2+ antagonists [10] are potential treatment options for overactive bladder. In addition, again like in urothelial cells, the membrane channel CALHM1 transports Ca2+ into (detrusor) muscle cells and it regulates the release of ATP by these cells [8], with increased ATP release having been associated with UUI [9-11]. Further, as in urothelial cells, CALHM1 is also involved in controlling APP cleavage and Aβ levels in/by muscle cells [180]. In addition, RYR1 complexes with **CACNB1** [218]. RYR1 is a membrane protein located in the sarcoplasmic reticulum (SR) that mediates the release of Ca2+ from the SR into the cytoplasm, which subsequently, triggers muscle contractions [219, 220]. RYR1 is activated by Ca2+ and ATP [221] and a decrease of RYR1 expression has been associated with overactive bladder [23]. In neurons, RYR1 is also involved in increasing the release of Aβ [222] (not shown).

Furthermore, as already indicated above, **RAMP2** is a receptor for adrenomedullin (ADM), which is expressed by both the urothelium and bladder (smooth) muscle [200] and of which increased levels have been linked to detrusor instability [4]. Lastly, in addition to urothelial cells [80], the bicarbonate transporter **SLC4A4** (see above) is expressed in muscle cells [223, 224] and, as in urothelial cells, it is (negatively) regulated by the **WNK4-**NEDD4L cascade [118, 119] (see above)**.**

Other cells (not shown)

Two landscape proteins were not shown in the landscape figure, as they are specifically/predominantly expressed - and function - in other cell types. First, **MTTP** is specifically expressed in/by liver cells and is involved in the assembly and secretion of plasma lipoproteins that contain APOB [5, 225, 226]. Second, **SHISA7** is mainly expressed in the brain, and it regulates gamma-aminobutyric acid type A receptor (GABA(A)R) trafficking and AMPA-type GL receptors (AMPAR) [5]. In this respect, it is interesting that GABA(A)R agonists inhibit detrusor contractions and may therefore be a useful treatment option for UUI [187].

Supplementary text S2 – Methodology of GWASs

Richter et al. 2015 updated GWAS [1]

The Women's Health Initiative (WHI) is a long-term, multi-site longitudinal study funded by the National Heart, Lung, and Blood Institute. The WHI collected data on urgency urinary incontinence from all participants at enrollment and closeout and from a subset of women at intervals throughout the study. The WHI-Genomics & Randomized Trials Network (GARNET) is a sub-study with 4,894 genotyped post-reproductive white women funded by the National Human Genome Research Institute.

We previously published a GWAS using the GARNET sub-study comparing 1,102 cases to 405 controls in the discovery cohort and 1,133 cases to 371 controls in the replication cohort (2,235 UI cases and 776 UI controls total). For this study, we updated the GARNET study UI GWAS using a modern automated analytic pipeline. The updated analysis includes a revised list of modeled covariates, ancestry defined by comparison to 1000 Genomes instead of HapMap, and an extra LiftOver step that allows for imputation against the most updated reference panel (TOPMed). See details below. The newly generated GWAS results closely resemble the original study and the top genes remained consistent [1] (https://github.com/RTIInternational/Genetic-Contributions-UUI). The data were downloaded from the database of Genotype and Phenotypes (dbGaP) (http://www.ncbi.nlm.nih.gov/sites/entrez?db=gap) Study Accessions: phs000200.v12.p3 and phs000315.v8.p3.

Quality control was performed with an open-source automated genotype array qc workflow developed by RTI International (https://github.com/RTIInternational/biocloud_gwas_workflows/tree/master/genotype_array_qc). Quality control was performed on each consent group separately. Genotype variants were filtered based on deviation from Hardy-Weinberg equilibrium (0.0001) and low call rate (0.03). Ancestral background of the samples was determined using STRUCTURE [227] with the 1000G reference populations EUR, CHB, and YRI using the definition EUR=CHB<0.25 && YRI<0.25. Samples were removed based on the following filtering criteria: relatedness, low call rate, excessive homozygosity, and sex discrepancies. The two consent groups were combined post genotype QC and imputed together with the TOPMed imputation server (https://imputation.biodatacatalyst.nhlbi.nih.gov/#!pages/home) using the TOPMed reference panel with Minimac4 v1.5.7 and Eagle v2.4. TOPMed imputation converts genomic coordinates to GRCh38. SNPs were filtered based on a sample MAF filter of 0.01 and imputation quality (rsq) of 0.8.

Cases were defined as women who at any time during the study (self-) reported they had symptoms of UUI, reporting that they usually leak when they feel the need to urinate and can't get to the toilet fast enough, more than once a month, and who leaked sufficiently to wet or soak their underpants or clothes. Controls were defined as women who initially did not report UUI and did not develop it during the study. Covariates included (1) age, defined as the participant age at enrollment and treated as a continuous variable, (2) obesity, defined as body mass index (BMI) of 30 kg/m2 or greater, (3) diabetes, defined as reporting being told by a doctor they had diabetes, (4) parity, defined as nulliparous, 1–2 births, 3 or more births, and (5) genotype principal components that explained at least 75% of the phenotypic variance. The final analysis included 2,322 cases and 817 controls.

The imputed genotype data were tested for association with UUI using logistic regression models. These tests were performed using the RVTESTS software [228] implemented in the open-source automated workflow (https://github.com/RTIInternational/biocloud_gwas_workflows/tree/master/association_testing/rvtests/ developed by RTI International.

A gene analysis was then performed using FUMA [229] (https://fuma.ctglab.nl/) FUMA requires GRCh37 coordinates, so the GWAS summary statistics were then converted back to GRCh37 from GRCh38 LiftOver (http://hgdownload.soe.ucsc.edu/admin/exe/linux.x86_64/liftOver).

GWAS by Penney et al. [2]

Participants in the GWAS came from both the Nurses' Health Study (NHS) and the second NHS (NHSII).

Phenotyping was performed using the answers to (seven) biennial questionnaires. The following questions were used to determine if patients experienced urinary incontinence: 1) "During the past 12 months, how often have you leaked urine or lost control of your urine?" with response options of: never, <1/month, 1/month, 2–3x/month, 1/week, almost every day and 2) "When you lose urine, how much usually leaks?" with response options of: a few drops, enough to wet your underwear, enough to wet outer clothing, enough to wet the floor. Participants were classified as cases (UUI) if they reported at least weekly urinary incontinence on a majority of questionnaires (≥4 in NHS, ≥3 in NHSII) and most leaking episodes related to a feeling of urgency. Controls were women with GWAS data who reported never experiencing urinary incontinence or no more than leaking a few drops less than once a month on all questionnaires to which they responded; all eligible participants must have responded to the majority of questionnaires. Furthermore, covariates for the UI analyses were included from the questionnaire when a participant first answered the urine leakage questions. These included age (continuous), BMI (<25, 25 to <30, and ≥30), parity (nulliparous, 1–2 births, 3 or more births), and Type II diabetes (yes/no). For those missing BMI (n=8) or parity (n=63), the median category was used.

Genotyping was performed using five different arrays in different genotyping centers. Standard quality control filters for call rate, Hardy-Weinberg equilibrium (HWE), and other measures were applied to the genotyped SNPs and samples. Studies were grouped by genotyping platform. Each grouped data set was imputed using the 1000 Genomes Project ALL Phase 3 Version 5 as the reference panel, resulting in 1,410,640 variants across the five platforms (each SNP is present in 1-5 platforms). Further, the analysis was restricted to participants with self-reported European ancestry and outliers that were identified as non-white by principal component analysis (PCA) were removed.

After phenotyping and genotyping the participants, a GWAS using a case-control design was conducted (1,942 UUI cases and 4,811 controls). The RVTests program was used to test the association of all 1,410,640 SNPs with UUI, using a logistic regression model and adjusting for the top 4 principal components (PCs) - to account for population structure - as well as age, BMI, parity, and Type II diabetes. Each genotyping platform-grouped data set was analyzed

separately. SNPs with poor imputation quality (R^2<0.3) were excluded. We also applied a minor allele frequency (MAF) threshold to each platform, which varied depending on the number of cases and controls, and was therefore different for each analysis. Subsequently, a meta-analysis was conducted to combine the results from each platform using a fixed effects model in METAL.

GWAS by Cartwright et al. [3]

Participants in the GWAS came from the Northern Finnish Birth Cohort 1966 (NFBC1966), the United Kingdom Twin Cohort (TwinsUK), and the Avon Longitudinal Study of Parents and Children (ALSPAC).

Phenotyping was performed using different self-reported questionnaires. For the NFBC1966, a Finnish translation and modification of the Danish Prostatic Symptom Score (DAN-PSS) questions related to UUI ('Do you experience an imperative (strong) urge to urinate?' and 'Is the urge to urinate so strong that urine starts to flow before you reach the toilet?') was used for phenotyping the participants. For TwinsUK, participants were considered UUI cases when they reported to have UUI or reported to experience urinary incontinence related to rushing to the WC. For the ALSPAC cohort, only the phenotypes collected in 2002/2003 were used. In addition, ALSPAC participants were considered UUI cases if they affirmatively answered 'Does urine leak before you can get to the toilet?'. Further details about UUI phenotype harmonization are available in the supplementary methods of the paper by Cartwright et al. [3].

As for genotyping the GWAS participants from the three cohorts, this has been extensively described by the teams that conducted this work in previous publications (see Cartwright et al. [3] for more details). After genotyping, imputation of the data was performed using the 1000 Genomes Phase 1 data, IMPUTE v2 (Twins UK / NFBC1966) or MiniMac (ALSPAC). Although exact procedures differ slightly between cohorts, the principles for deriving and quality controlling the genotyping data were consistent. The SNP quality control procedures in the three cohorts included exclusions for HWE deviation ($p<0.00001$ and $p<0.001$), MAF<1%, and call rate <95%.

After phenotyping and genotyping the participants, a GWAS using a case-control design was conducted (870 UUI cases and 8,127 controls). The primary GWAS analyses were run separately for the three cohorts. An almost identical analytic strategy was used in each cohort. With the dichotomous case definitions as each outcome, logistic regression models were used, based on expected allelic dosage model for each variant. For the ALSPAC data, these models were run using MACH2DAT, while for TwinsUK and NFBC1966, the models were run using SNPTEST (https://www.mathgen.stats.ox.ac.uk/genetics_software/snptest/snptest.html).

The analyses were firstly run unadjusted, and then adjusted for age (continuous), BMI (continuous), and parity (discrete metric). Unmappable SNPs without current known positions were excluded. Poorly imputed SNPs (info metric or R^2<0.6) were also excluded. For NFBC1966, the analyses were also adjusted for the PCs derived from a principal component analysis (PCA) of all directly genotyped variants, to control for population substructure.

Subsequently, meta-analyses between the three discovery cohorts were conducted using METAL (http://www.sph.umich.edu/csg/abecasis/metal/).

HUNT GWAS (this study)

Participants in the GWAS came from the Nord-Trøndelag Health Study (HUNT), a large population-based cohort from the county Nord-Trøndelag in Norway. All residents in the county, aged 20 years and older, have been invited to participate. Data was collected through three cross-sectional surveys, HUNT1 (1984-1986), HUNT2 (1995-1997) and HUNT3 (2006-2008), and has been described in detail previously [230], with the fourth survey recently completed (HUNT4, 2017-2019). DNA from whole blood was collected from HUNT2 and HUNT3, with genotypes available from 71,860 participants. All genotyped participants have signed a written informed consent regarding the use of data from questionnaires, biological samples and linkage to other registries for research purposes.

All participants answered a questionnaire in order to determine their UUI phenotype. Participants were considered UUI cases when they reported involuntary loss of urine at least once or more times a month, and when they reported leaking urine in connection with a sudden and strong urge to void and a negative answer to having involuntary loss of urine when they cough, sneeze, laugh, or lift something heavy. Participants were considered controls if they reported no involuntary loss of urine or less than once a month, with the leaking of urine not in connection with a sudden and strong urge to void. Participants were excluded when they were pregnant or within 9 months (or 42 weeks) peripartum.

As for genotyping the participants (that were all from HUNT2 and HUNT3, see above), DNA from blood samples was genotyped using one of three different Illumina HumanCoreExome arrays (HumanCoreExome12 v1.0, HumanCoreExome12 v1.1 and UM HUNT Biobank v1.0). Samples that failed to reach a 99% call rate, had contamination >2.5% as estimated with BAF Regress [231], large chromosomal copy number variants, lower call rate of a technical duplicate pair and twins, gonosomal constellations other than XX and XY, or whose inferred sex contradicted the reported gender, were excluded. Samples that passed quality control were analyzed in a second round of genotype calling following the Genome Studio quality control protocol described elsewhere [232]. Genomic position, strand orientation and the reference allele of genotyped variants were determined by aligning their probe sequences against the human genome (Genome Reference Consortium Human genome build 37 and revised Cambridge Reference Sequence of the human mitochondrial DNA;http://genome.ucsc.edu) using BLAT [233]. Variants were excluded if (1) their probe sequences could not be perfectly mapped to the reference genome, cluster separation was <0.3, Gentrain score was <0.15, showed deviations from HWE in unrelated samples of European ancestry with p <0.0001), their call rate was <99%, or another assay with higher call rate genotyped the same variant.

Ancestry of all samples was inferred by projecting all genotyped samples into the space of the principal components of the Human Genome Diversity Project (HGDP) reference panel (938 unrelated individuals; downloaded fromhttp://csg.sph.umich.edu/chaolong/LASER/) [234, 235], using PLINK v1.90 [236]. Recent European ancestry was defined as samples that fell into an ellipsoid spanning exclusively European populations of the HGDP panel. The different

arrays were harmonized by reducing to a set of overlapping variants and excluding variants that showed frequency differences >15% between data sets, or that were monomorphic in one and had MAF >1% in another data set. The resulting genotype data were phased using Eagle2 v2.3 [237]. Subsequently, imputation was performed on the samples of recent European ancestry using Minimac3 (v2.0.1,http://genome.sph.umich.edu/wiki/Minimac3) [238] with default settings (2.5 Mb reference-based chunking with 500kb windows) and a customized Haplotype Reference consortium release 1.1 (HRC v1.1) for autosomal variants and HRC v1.1 for chromosome X variants [239]. The customized reference panel represented the merged panel of two reciprocally imputed reference panels: (1) 2,201 low-coverage whole-genome sequences samples from the HUNT study and (2) HRC v1.1 with 1,023 HUNT WGS samples removed before merging. Imputed variants with R2<0.3 were also excluded, resulting in over 24.9 million well-imputed variants.

After phenotyping and genotyping the participants, a GWAS using a case-control design was conducted (693 UUI cases and 23,055 controls). For this, we used SAIGE Version 0.44.6.4 (https://github.com/weizhouUMICH/SAIGE, and https://www.medrxiv.org/content/10.1101/2021.07.12.21260400v2) with default QC parameters - apart from using a minimal MAF<0.001 and minimal minor allele count (MAC) ≥3) - and adjusted for birth year, BMI and parity.

GWAS meta-analysis

We performed a GWAS meta-analysis using the tool METAL (build date 5 May 2020; [240] and the summary statistics from the GWAS by Penney et al. (N=6753), the GWAS by Cartwright et al. (N=8971), and the GWAS in the HUNT samples (N=18537). All GWAS summary statistics were filtered prior to analysis (MAF > 0.01, INFO > 0.8, N > 0.67, indels, ambiguous SNPs and valid ranges for P-values and standard errors) and the total number of SNPs included was 7,869,234. The meta analysis (N=34,261) was conducted using default settings of taking into account sample size as weights and the direction of effect. Automatic genomic control correction was applied to account for small amounts of population stratification or unaccounted for relatedness.

Supplementary Text S3 – Detailed description of methodology for PRS-based analyses

These polygenic risk score (PRS)-based analyses were performed to assess the genetic overlap between Alzheimer's disease (AD) and UUI. For this, the publicly available summary statistics data from three GWASs of AD - i.e., the FinnGen consortium (https://www.finngen.fi/en/access_results), Jansen et al. [34], and Bellenguez et al. [35] - were used as 'base samples' and all four GWASs of UUI were used as 'target samples'.

The GWAS by the FinnGen consortium encompasses an AD cohort of 1,798 cases with a clinical ICD-10 diagnosis of AD and 72,206 controls. The GWAS by Jansen et al. encompasses 71,880 AD and AD 'by proxy' cases, e.g., people with a parental history of diagnosed AD, which show a strong genetic correlation with AD [241], and 383,378 controls. The GWAS by Bellenguez et al. examined 111,326 AD and AD 'by proxy' cases and 677,663 controls. The cohort from the AD GWAS by Bellenguez overlaps with the cohorts from both the GWAS by the FinnGen cohort and Jansen et al. As far as we have been able to ascertain, there is no sample overlap between the base and target samples.

In addition, we performed PRS-based analyses to assess the genetic overlap between the Aβ42/Aβ40 blood level ratio and UUI. For this, the publicly available summary statistics data from the GWAS of the Aβ42/Aβ40 blood level ratio of Damotte et al. [36] was used as 'base sample' and three of the four GWASs of UUI – the GWASs by Cartwright et al. [3] and Penny et al. [2], and the HUNT GWAS (this study) – were used as 'target samples'. The GWAS by Damotte et al. encompasses 12,369 non-demented, European-descent participants from eight population-based studies [36], and again, as far as we have been able to ascertain, there is no sample overlap between the base and target samples.

PRSice version 1 was used to perform the PRS-based analyses [242]. First, clumping was performed based on the p values of the SNPs in the base samples to select the most significant SNP among correlated SNPs that are in LD (R^2>0.25) within a window of 500kb [243, 244]. Subsequently, the summary-level PRS was calculated by regressing the weights of selected AD risk SNPs (based on their p value in the AD GWASs) on to the calculated weighted multi-SNP risk scores of UUI, using the gtx package implemented in PRSice. The PRS-based analyses were performed for all SNPs that exceed seven default p value thresholds (p_{TS}), i.e., 0.001, 0.005, 0.1, 0.2, 0.3, 0.4, and 0.5. In addition, we performed a correction for multiple testing using a stringent Bonferroni-corrected threshold for the groups of PRS-based analyses, i.e., (1) a Bonferroni-corrected threshold for all 7 tested p_{TS} and all 12 phenotypes (combinations of 3 x AD and 4 x UUI), i.e., p < 0.05/ 84 tests (= 7 p_{TS} x 12 phenotypes) = 5.95E-04 (designated as significant genetic overlap), and (2) a Bonferroni-corrected threshold for all 7 tested p_{TS} and all 3 phenotypes (combinations of 1 x Aβ42/Aβ40 blood level ratio and 3 x UUI), i.e., p < 0.05/ 21 tests (= 7 p_{TS} x 3 phenotypes) = 2.38E-03 (designated as significant genetic overlap).
For those PRS-based analyses for which we found (nominally) significant genetic overlap, we also performed a SNP effect concordance analysis (SECA) to determine the direction (or 'concordance') of the overlap (see [245] for more details about SECA).

Supplementary Figure S1

This figure shows a radial presentation of the six merged, overlapping networks resulting from the network enrichment analysis. APP is located in the center of a very large number of protein-protein interactions. All the proteins encoded by genes from the genome-wide association studies (GWASs) of urgency urinary incontinence (UUI) are indicated in various degrees of redness. The more red a protein is, the lower the gene-wide p value for the corresponding gene is (see Methods section).

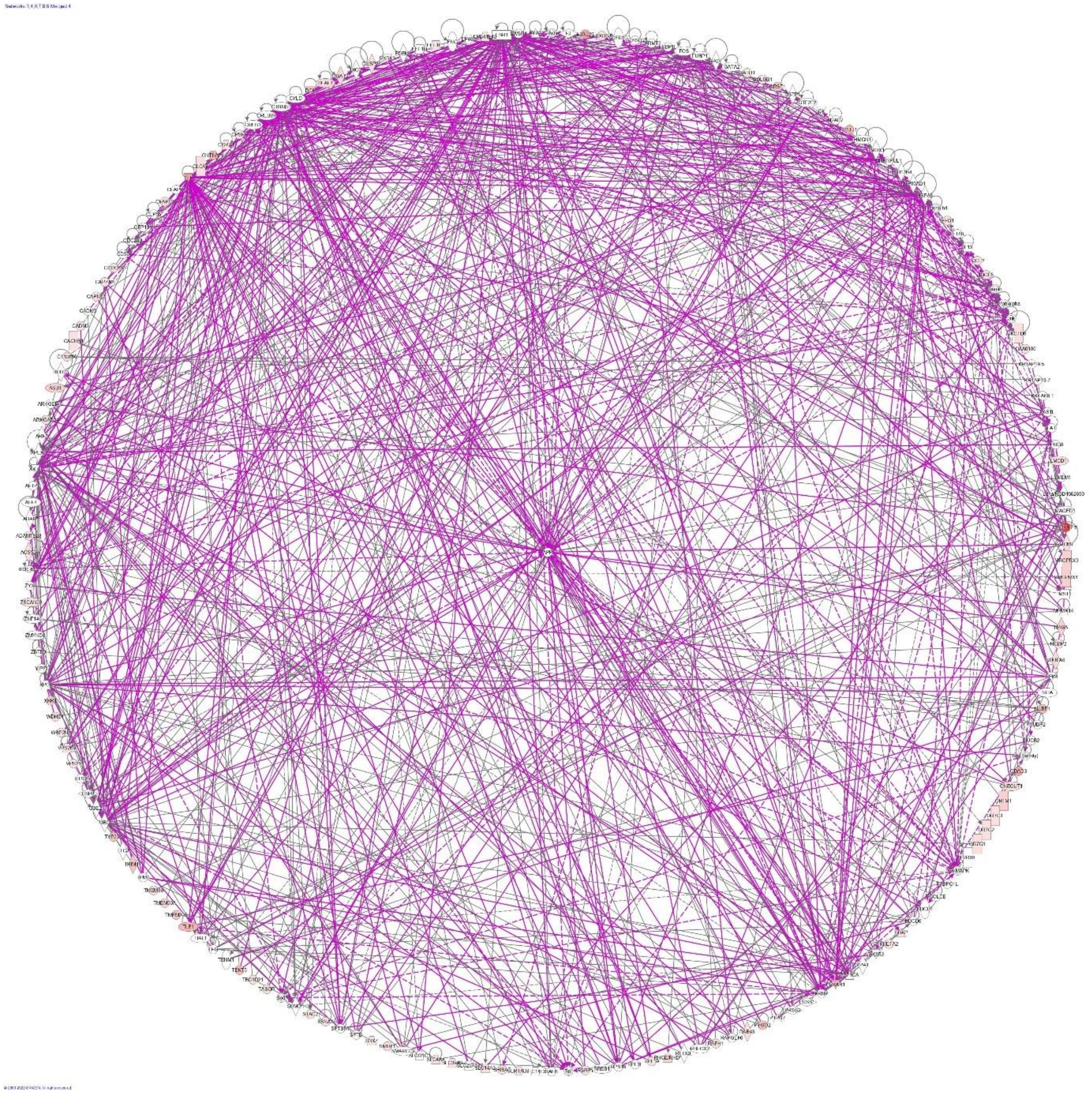

Supplementary Figure S3. Bar plots from PRSice [242] showing results at seven broad P-value thresholds (P_T) for shared genetic etiology between AD (based on the GWAS by Bellenguez et al. [35]) and UUI (based on the GWAS by Cartwright et al. [3]) (see Materials and methods). The numbers above the bars indicate the P-values for shared genetic etiology, and these P-values were corrected using the Bonferroni method.

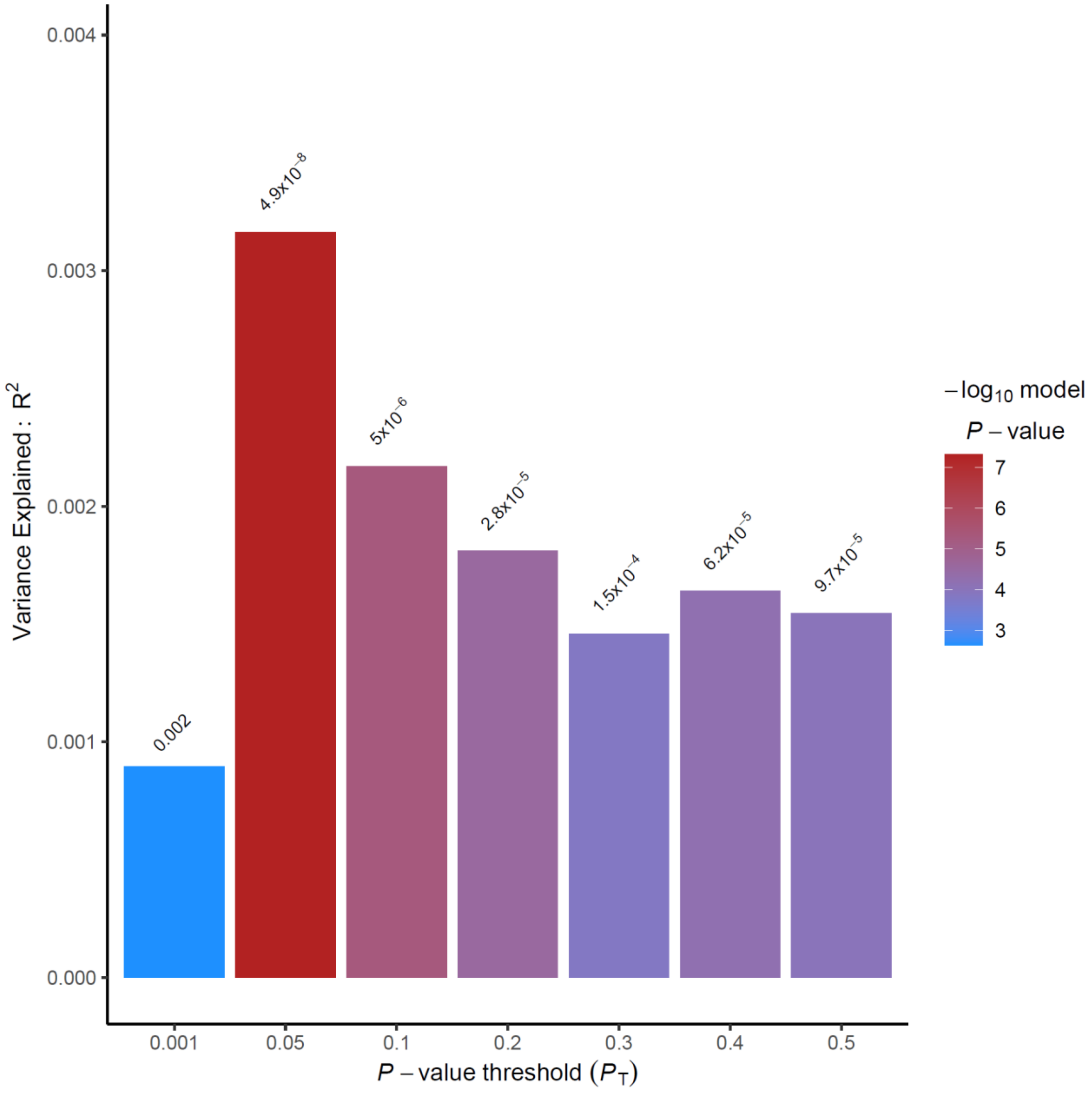

Supplementary Figure S4. Bar plots from PRSice [242] showing results at seven broad P-value thresholds (P_T) for shared genetic etiology between the Aβ42/Aβ40 blood level ratio (based on the GWAS by Damotte et al. [36]) and UUI (based on the GWAS by Cartwright et al. [3]) (see Materials and methods). The numbers above the bars indicate the P-values for shared genetic etiology, and these P-values were corrected using the Bonferroni method.

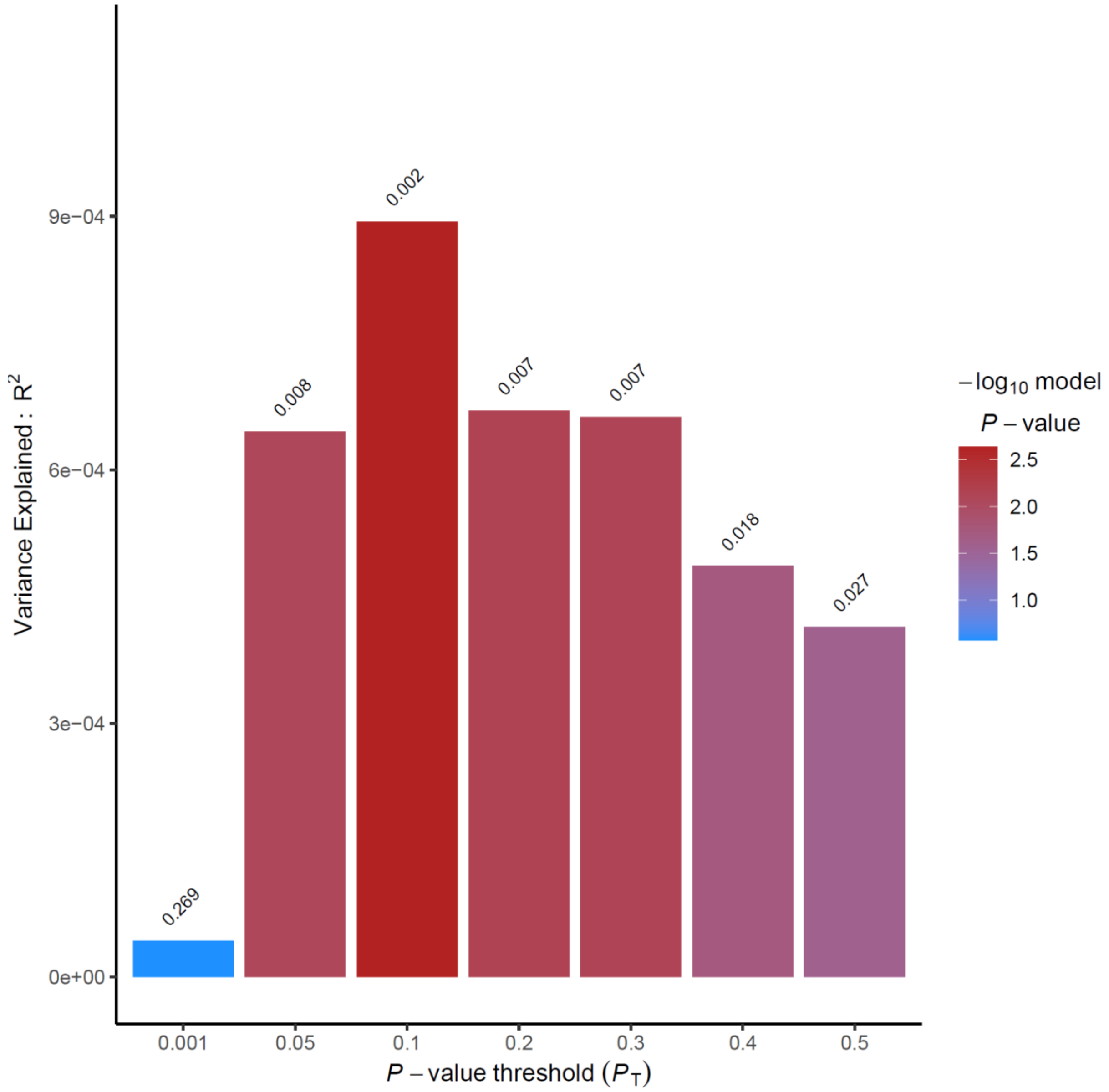

References

1. Richter, H. E.; Whitehead, N.; Arya, L.; Ridgeway, B.; Allen-Brady, K.; Norton, P.; Sung, V.; Shepherd, J. P.; Komesu, Y.; Gaddis, N.; Fraser, M. O.; Tan-Kim, J.; Meikle, S.; Page, G. P.; Pelvic Floor Disorders, N., Genetic contributions to urgency urinary incontinence in women. *J Urol* **2015,** 193, (6), 2020-7.
2. Penney, K. L.; Townsend, M. K.; Turman, C.; Glass, K.; Staller, K.; Kraft, P.; Grodstein, F.; Minassian, V. A., Genome-Wide Association Study for Urinary and Fecal Incontinence in Women. *J Urol* **2020,** 203, (5), 978-983.
3. Cartwright, R.; Franklin, L.; Tikkinen, K. A. O.; Kalliala, I.; Miotla, P.; Rechberger, T.; Offiah, I.; McMahon, S.; O'Reilly, B.; Lince, S.; Kluivers, K.; Post, W. M.; Poelmans, G.; Palmer, M. R.; Wessells, H.; Wong, A.; Kuh, D.; Kivimaki, M.; Kumari, M.; Mangino, M.; Spector, T.; Guggenheim, J. A.; Lehne, B.; De Silva, N. M. G.; Evans, D. M.; Lawlor, D.; Karhunen, V.; Mannikko, M.; Marczak, M.; Bennett, P. R.; Khullar, V.; Jarvelin, M. R.; Walley, A., Genome-Wide Association Study Identifies Two Novel Loci Associated with Female Stress and Urgency Urinary Incontinence. *J Urol* **2021,** 206, (3), 679-687.
4. Balat, A.; Sarica, K.; Cekmen, M.; Yurekli, M.; Yagci, F.; Erbagci, A., Adrenomedullin and nitric oxide in children with detrusor instability. *Pediatr Nephrol* **2003,** 18, (5), 422-5.
5. UniProt, C., UniProt: the Universal Protein Knowledgebase in 2025. *Nucleic Acids Res* **2025,** 53, (D1), D609-D617.
6. Baytaroglu, C.; Sevgili, E., Association of Metabolic Syndrome Components and Overactive Bladder in Women. *Cureus* **2021,** 13, (4), e14765.
7. Cornu, J. N.; Merlet, B.; Cussenot, O.; Cancel-Tassin, G.; Ciofu, C.; Amarenco, G.; Haab, F., Genetic susceptibility to urinary incontinence: implication of polymorphisms of androgen and oestrogen pathways. *World J Urol* **2011,** 29, (2), 239-42.
8. Sana-Ur-Rehman, H.; Markus, I.; Moore, K. H.; Mansfield, K. J.; Liu, L., Expression and localization of pannexin-1 and CALHM1 in porcine bladder and their involvement in modulating ATP release. *Am J Physiol Regul Integr Comp Physiol* **2017,** 312, (5), R763-R772.
9. Kumar, V.; Chapple, C. R.; Rosario, D.; Tophill, P. R.; Chess-Williams, R., In vitro release of adenosine triphosphate from the urothelium of human bladders with detrusor overactivity, both neurogenic and idiopathic. *Eur Urol* **2010,** 57, (6), 1087-92.
10. Badawi, J. K.; Langbein, S., Selective beta-adrenoceptor agonists, calcium antagonists and potassium channel openers as a possible medical treatment of the overactive bladder and urge incontinence. *Pharmazie* **2006,** 61, (3), 175-8.
11. Antunes-Lopes, T.; Cruz, F., Urinary Biomarkers in Overactive Bladder: Revisiting the Evidence in 2019. *Eur Urol Focus* **2019,** 5, (3), 329-336.
12. Masuda, H.; Kihara, K.; Saito, K.; Matsuoka, Y.; Yoshida, S.; Chancellor, M. B.; de Groat, W. C.; Yoshimura, N., Reactive oxygen species mediate detrusor overactivity via sensitization of afferent pathway in the bladder of anaesthetized rats. *BJU Int* **2008,** 101, (6), 775-80.
13. Tyagi, P.; Barclay, D.; Zamora, R.; Yoshimura, N.; Peters, K.; Vodovotz, Y.; Chancellor, M., Urine cytokines suggest an inflammatory response in the overactive bladder: a pilot study. *Int Urol Nephrol* **2010,** 42, (3), 629-35.
14. Robinson, D.; Cardozo, L., Managing overactive bladder. *Climacteric* **2019,** 22, (3), 250-256.
15. Hillard, T., The postmenopausal bladder. *Menopause Int* **2010,** 16, (2), 74-80.
16. Robinson, D.; Toozs-Hobson, P.; Cardozo, L., The effect of hormones on the lower urinary tract. *Menopause Int* **2013,** 19, (4), 155-62.
17. Kullmann, F. A.; Limberg, B. J.; Artim, D. E.; Shah, M.; Downs, T. R.; Contract, D.; Wos, J.; Rosenbaum, J. S.; de Groat, W. C., Effects of beta3-adrenergic receptor activation on rat urinary bladder hyperactivity induced by ovariectomy. *J Pharmacol Exp Ther* **2009,** 330, (3), 704-17.

18. Liang, W.; Afshar, K.; Stothers, L.; Laher, I., The influence of ovariectomy and estrogen replacement on voiding patterns and detrusor muscarinic receptor affinity in the rat. *Life Sci* **2002,** 71, (3), 351-62.
19. Juliato, C. R.; Baccaro, L. F.; Pedro, A. O.; Costa-Paiva, L.; Lui-Filho, J.; Pinto-Neto, A. M., Subjective urinary urgency in middle age women: A population-based study. *Maturitas* **2016,** 85, 82-7.
20. Pillalamarri, N.; Shalom, D. F.; Pilkinton, M. L.; Winkler, H. A.; Chatterjee, P. K.; Solanki, M.; Metz, C. N., Inflammatory Urinary Cytokine Expression and Quality of Life in Patients With Overactive Bladder. *Female Pelvic Med Reconstr Surg* **2018,** 24, (6), 449-453.
21. Kanasaki, K.; Yu, W.; von Bodungen, M.; Larigakis, J. D.; Kanasaki, M.; Ayala de la Pena, F.; Kalluri, R.; Hill, W. G., Loss of beta1-integrin from urothelium results in overactive bladder and incontinence in mice: a mechanosensory rather than structural phenotype. *FASEB J* **2013,** 27, (5), 1950-61.
22. Post, W. M.; Ruiz-Zapata, A. M.; Grens, H.; de Vries, R. B. M.; Poelmans, G.; Coenen, M. J. H.; Janssen, D. A. W.; Heesakkers, J.; Oosterwijk, E.; Kluivers, K. B., Genetic variants and expression changes in urgency urinary incontinence: A systematic review. *Neurourol Urodyn* **2020,** 39, (8), 2089-2110.
23. Jiang, H. H.; Song, B.; Lu, G. S.; Wen, Q. J.; Jin, X. Y., Loss of ryanodine receptor calcium-release channel expression associated with overactive urinary bladder smooth muscle contractions in a detrusor instability model. *BJU Int* **2005,** 96, (3), 428-33.
24. Sonmez, M. G.; Goger, Y. E.; Ecer, G.; Atici, A.; Ozkent, M. S.; Ozturk, A., Effects of urine alkalinization with sodium bicarbonate orally on lower urinary tract symptoms in female patients: a pilot study. *Int Urogynecol J* **2018,** 29, (7), 1029-1033.
25. Sonmez, M. G.; Ecer, G.; Atici, A.; Ozkent, M. S.; Iyisoy, M. S.; Ozturk, A., Comparison of Oral Sodium Bicarbonate and Solifenacin Treatment in Female Overactive Bladder Patients With Acidic Urine pH. *Female Pelvic Med Reconstr Surg* **2020,** 26, (10), 649-653.
26. Irkilata, L.; Aydin, M.; Riza Aydin, H.; Cihan Demirel, H.; Kadihasanoglu, M.; Kemal Atilla, M., Intravesical Sodium Chondroitin Sulphate to Treat Overactive Bladder: Preliminary Result. *Int Neurourol J* **2015,** 19, (2), 85-9.
27. Morelli, M.; Mocciaro, R.; Venturella, R.; Albano, A.; Sacchinelli, A.; Zullo, F., Hyaluronic acid-chondroitin sulfate: a potential factor to select pure stress urinary incontinence in patients with interstitial cystitispainful bladder syndrome and mixed incontinence symptoms. *Minerva Ginecol* **2015,** 67, (2), 121-5.
28. de Groat, W. C.; Yoshimura, N., Afferent nerve regulation of bladder function in health and disease. *Handb Exp Pharmacol* **2009**, (194), 91-138.
29. Schwen, Z.; Matsuta, Y.; Shen, B.; Wang, J.; Roppolo, J. R.; de Groat, W. C.; Tai, C., Involvement of 5-HT3 receptors in pudendal inhibition of bladder overactivity in cats. *Am J Physiol Renal Physiol* **2013,** 305, (5), F663-71.
30. Larson, J. A.; Ogagan, P. D.; Chen, G.; Shen, B.; Wang, J.; Roppolo, J. R.; de Groat, W. C.; Tai, C., Involvement of metabotropic glutamate receptor 5 in pudendal inhibition of nociceptive bladder activity in cats. *J Physiol* **2011,** 589, (Pt 23), 5833-43.
31. Yin, L.; Zhang, Z.; Zheng, Y.; Hou, L.; Zhao, C. G.; Wang, X. L.; Jiang, K. L.; Du, Y., Clinical Correlation Between Overactive Bladder and Allergy in Children. *Front Pediatr* **2021,** 9, 813161.
32. Zheng, Y.; Zhang, Z.; Hou, L.; Wang, X.; Jiang, K.; Zhang, S.; Du, Y., Clinical Observation of the Correlation Between Overactive Bladder and Atopic Constitution in Children. *Front Pediatr* **2021,** 9, 646118.
33. Wrobel, A.; Rechberger, E.; Rechberger, T., The influence of duloxetine on detrusor overactivity in rats with depression induced by 13-cis-retinoic acid. *Int Urogynecol J* **2018,** 29, (7), 987-995.
34. Jansen, I. E.; Savage, J. E.; Watanabe, K.; Bryois, J.; Williams, D. M.; Steinberg, S.; Sealock, J.; Karlsson, I. K.; Hagg, S.; Athanasiu, L.; Voyle, N.; Proitsi, P.; Witoelar, A.; Stringer, S.; Aarsland,

D.; Almdahl, I. S.; Andersen, F.; Bergh, S.; Bettella, F.; Bjornsson, S.; Braekhus, A.; Brathen, G.; de Leeuw, C.; Desikan, R. S.; Djurovic, S.; Dumitrescu, L.; Fladby, T.; Hohman, T. J.; Jonsson, P. V.; Kiddle, S. J.; Rongve, A.; Saltvedt, I.; Sando, S. B.; Selbaek, G.; Shoai, M.; Skene, N. G.; Snaedal, J.; Stordal, E.; Ulstein, I. D.; Wang, Y.; White, L. R.; Hardy, J.; Hjerling-Leffler, J.; Sullivan, P. F.; van der Flier, W. M.; Dobson, R.; Davis, L. K.; Stefansson, H.; Stefansson, K.; Pedersen, N. L.; Ripke, S.; Andreassen, O. A.; Posthuma, D., Genome-wide meta-analysis identifies new loci and functional pathways influencing Alzheimer's disease risk. *Nat Genet* **2019,** 51, (3), 404-413.

35. Bellenguez, C.; Kucukali, F.; Jansen, I. E.; Kleineidam, L.; Moreno-Grau, S.; Amin, N.; Naj, A. C.; Campos-Martin, R.; Grenier-Boley, B.; Andrade, V.; Holmans, P. A.; Boland, A.; Damotte, V.; van der Lee, S. J.; Costa, M. R.; Kuulasmaa, T.; Yang, Q.; de Rojas, I.; Bis, J. C.; Yaqub, A.; Prokic, I.; Chapuis, J.; Ahmad, S.; Giedraitis, V.; Aarsland, D.; Garcia-Gonzalez, P.; Abdelnour, C.; Alarcon-Martin, E.; Alcolea, D.; Alegret, M.; Alvarez, I.; Alvarez, V.; Armstrong, N. J.; Tsolaki, A.; Antunez, C.; Appollonio, I.; Arcaro, M.; Archetti, S.; Pastor, A. A.; Arosio, B.; Athanasiu, L.; Bailly, H.; Banaj, N.; Baquero, M.; Barral, S.; Beiser, A.; Pastor, A. B.; Below, J. E.; Benchek, P.; Benussi, L.; Berr, C.; Besse, C.; Bessi, V.; Binetti, G.; Bizarro, A.; Blesa, R.; Boada, M.; Boerwinkle, E.; Borroni, B.; Boschi, S.; Bossu, P.; Brathen, G.; Bressler, J.; Bresner, C.; Brodaty, H.; Brookes, K. J.; Brusco, L. I.; Buiza-Rueda, D.; Burger, K.; Burholt, V.; Bush, W. S.; Calero, M.; Cantwell, L. B.; Chene, G.; Chung, J.; Cuccaro, M. L.; Carracedo, A.; Cecchetti, R.; Cervera-Carles, L.; Charbonnier, C.; Chen, H. H.; Chillotti, C.; Ciccone, S.; Claassen, J.; Clark, C.; Conti, E.; Corma-Gomez, A.; Costantini, E.; Custodero, C.; Daian, D.; Dalmasso, M. C.; Daniele, A.; Dardiotis, E.; Dartigues, J. F.; de Deyn, P. P.; de Paiva Lopes, K.; de Witte, L. D.; Debette, S.; Deckert, J.; Del Ser, T.; Denning, N.; DeStefano, A.; Dichgans, M.; Diehl-Schmid, J.; Diez-Fairen, M.; Rossi, P. D.; Djurovic, S.; Duron, E.; Duzel, E.; Dufouil, C.; Eiriksdottir, G.; Engelborghs, S.; Escott-Price, V.; Espinosa, A.; Ewers, M.; Faber, K. M.; Fabrizio, T.; Nielsen, S. F.; Fardo, D. W.; Farotti, L.; Fenoglio, C.; Fernandez-Fuertes, M.; Ferrari, R.; Ferreira, C. B.; Ferri, E.; Fin, B.; Fischer, P.; Fladby, T.; Fliessbach, K.; Fongang, B.; Fornage, M.; Fortea, J.; Foroud, T. M.; Fostinelli, S.; Fox, N. C.; Franco-Macias, E.; Bullido, M. J.; Frank-Garcia, A.; Froelich, L.; Fulton-Howard, B.; Galimberti, D.; Garcia-Alberca, J. M.; Garcia-Gonzalez, P.; Garcia-Madrona, S.; Garcia-Ribas, G.; Ghidoni, R.; Giegling, I.; Giorgio, G.; Goate, A. M.; Goldhardt, O.; Gomez-Fonseca, D.; Gonzalez-Perez, A.; Graff, C.; Grande, G.; Green, E.; Grimmer, T.; Grunblatt, E.; Grunin, M.; Gudnason, V.; Guetta-Baranes, T.; Haapasalo, A.; Hadjigeorgiou, G.; Haines, J. L.; Hamilton-Nelson, K. L.; Hampel, H.; Hanon, O.; Hardy, J.; Hartmann, A. M.; Hausner, L.; Harwood, J.; Heilmann-Heimbach, S.; Helisalmi, S.; Heneka, M. T.; Hernandez, I.; Herrmann, M. J.; Hoffmann, P.; Holmes, C.; Holstege, H.; Vilas, R. H.; Hulsman, M.; Humphrey, J.; Biessels, G. J.; Jian, X.; Johansson, C.; Jun, G. R.; Kastumata, Y.; Kauwe, J.; Kehoe, P. G.; Kilander, L.; Stahlbom, A. K.; Kivipelto, M.; Koivisto, A.; Kornhuber, J.; Kosmidis, M. H.; Kukull, W. A.; Kuksa, P. P.; Kunkle, B. W.; Kuzma, A. B.; Lage, C.; Laukka, E. J.; Launer, L.; Lauria, A.; Lee, C. Y.; Lehtisalo, J.; Lerch, O.; Lleo, A.; Longstreth, W., Jr.; Lopez, O.; de Munain, A. L.; Love, S.; Lowemark, M.; Luckcuck, L.; Lunetta, K. L.; Ma, Y.; Macias, J.; MacLeod, C. A.; Maier, W.; Mangialasche, F.; Spallazzi, M.; Marquie, M.; Marshall, R.; Martin, E. R.; Montes, A. M.; Rodriguez, C. M.; Masullo, C.; Mayeux, R.; Mead, S.; Mecocci, P.; Medina, M.; Meggy, A.; Mehrabian, S.; Mendoza, S.; Menendez-Gonzalez, M.; Mir, P.; Moebus, S.; Mol, M.; Molina-Porcel, L.; Montrreal, L.; Morelli, L.; Moreno, F.; Morgan, K.; Mosley, T.; Nothen, M. M.; Muchnik, C.; Mukherjee, S.; Nacmias, B.; Ngandu, T.; Nicolas, G.; Nordestgaard, B. G.; Olaso, R.; Orellana, A.; Orsini, M.; Ortega, G.; Padovani, A.; Paolo, C.; Papenberg, G.; Parnetti, L.; Pasquier, F.; Pastor, P.; Peloso, G.; Perez-Cordon, A.; Perez-Tur, J.; Pericard, P.; Peters, O.; Pijnenburg, Y. A. L.; Pineda, J. A.; Pinol-Ripoll, G.; Pisanu, C.; Polak, T.; Popp, J.; Posthuma, D.; Priller, J.; Puerta, R.; Quenez, O.; Quintela, I.; Thomassen, J. Q.; Rabano, A.; Rainero, I.; Rajabli, F.; Ramakers, I.; Real, L. M.; Reinders, M. J. T.; Reitz, C.; Reyes-Dumeyer, D.; Ridge, P.; Riedel-Heller, S.; Riederer, P.; Roberto, N.; Rodriguez-Rodriguez, E.; Rongve, A.; Allende, I. R.; Rosende-Roca, M.; Royo, J. L.; Rubino, E.; Rujescu, D.; Saez, M. E.;

Sakka, P.; Saltvedt, I.; Sanabria, A.; Sanchez-Arjona, M. B.; Sanchez-Garcia, F.; Juan, P. S.; Sanchez-Valle, R.; Sando, S. B.; Sarnowski, C.; Satizabal, C. L.; Scamosci, M.; Scarmeas, N.; Scarpini, E.; Scheltens, P.; Scherbaum, N.; Scherer, M.; Schmid, M.; Schneider, A.; Schott, J. M.; Selbaek, G.; Seripa, D.; Serrano, M.; Sha, J.; Shadrin, A. A.; Skrobot, O.; Slifer, S.; Snijders, G. J. L.; Soininen, H.; Solfrizzi, V.; Solomon, A.; Song, Y.; Sorbi, S.; Sotolongo-Grau, O.; Spalletta, G.; Spottke, A.; Squassina, A.; Stordal, E.; Tartan, J. P.; Tarraga, L.; Tesi, N.; Thalamuthu, A.; Thomas, T.; Tosto, G.; Traykov, L.; Tremolizzo, L.; Tybjaerg-Hansen, A.; Uitterlinden, A.; Ullgren, A.; Ulstein, I.; Valero, S.; Valladares, O.; Broeckhoven, C. V.; Vance, J.; Vardarajan, B. N.; van der Lugt, A.; Dongen, J. V.; van Rooij, J.; van Swieten, J.; Vandenberghe, R.; Verhey, F.; Vidal, J. S.; Vogelgsang, J.; Vyhnalek, M.; Wagner, M.; Wallon, D.; Wang, L. S.; Wang, R.; Weinhold, L.; Wiltfang, J.; Windle, G.; Woods, B.; Yannakoulia, M.; Zare, H.; Zhao, Y.; Zhang, X.; Zhu, C.; Zulaica, M.; Eadb; Gr@Ace; Degesco; Eadi; Gerad; Demgene; FinnGen; Adgc; Charge; Farrer, L. A.; Psaty, B. M.; Ghanbari, M.; Raj, T.; Sachdev, P.; Mather, K.; Jessen, F.; Ikram, M. A.; de Mendonca, A.; Hort, J.; Tsolaki, M.; Pericak-Vance, M. A.; Amouyel, P.; Williams, J.; Frikke-Schmidt, R.; Clarimon, J.; Deleuze, J. F.; Rossi, G.; Seshadri, S.; Andreassen, O. A.; Ingelsson, M.; Hiltunen, M.; Sleegers, K.; Schellenberg, G. D.; van Duijn, C. M.; Sims, R.; van der Flier, W. M.; Ruiz, A.; Ramirez, A.; Lambert, J. C., New insights into the genetic etiology of Alzheimer's disease and related dementias. *Nat Genet* **2022,** 54, (4), 412-436.

36. Damotte, V.; van der Lee, S. J.; Chouraki, V.; Grenier-Boley, B.; Simino, J.; Adams, H.; Tosto, G.; White, C.; Terzikhan, N.; Cruchaga, C.; Knol, M. J.; Li, S.; Schraen, S.; Grove, M. L.; Satizabal, C.; Amin, N.; Berr, C.; Younkin, S.; Alzheimer's Disease Neuroimaging, I.; Gottesman, R. F.; Buee, L.; Beiser, A.; Knopman, D. S.; Uitterlinden, A.; DeCarli, C.; Bressler, J.; DeStefano, A.; Dartigues, J. F.; Yang, Q.; Boerwinkle, E.; Tzourio, C.; Fornage, M.; Ikram, M. A.; Amouyel, P.; de Jager, P.; Reitz, C.; Mosley, T. H.; Lambert, J. C.; Seshadri, S.; van Duijn, C. M., Plasma amyloid beta levels are driven by genetic variants near APOE, BACE1, APP, PSEN2: A genome-wide association study in over 12,000 non-demented participants. *Alzheimers Dement* **2021,** 17, (10), 1663-1674.

37. Peyronnet, B.; Mironska, E.; Chapple, C.; Cardozo, L.; Oelke, M.; Dmochowski, R.; Amarenco, G.; Game, X.; Kirby, R.; Van Der Aa, F.; Cornu, J. N., A Comprehensive Review of Overactive Bladder Pathophysiology: On the Way to Tailored Treatment. *Eur Urol* **2019,** 75, (6), 988-1000.

38. Birder, L.; Andersson, K. E., Urothelial signaling. *Physiol Rev* **2013,** 93, (2), 653-80.

39. Baumkotter, F.; Schmidt, N.; Vargas, C.; Schilling, S.; Weber, R.; Wagner, K.; Fiedler, S.; Klug, W.; Radzimanowski, J.; Nickolaus, S.; Keller, S.; Eggert, S.; Wild, K.; Kins, S., Amyloid precursor protein dimerization and synaptogenic function depend on copper binding to the growth factor-like domain. *J Neurosci* **2014,** 34, (33), 11159-72.

40. Cha, H. J.; Shen, J.; Kang, J., Regulation of gene expression by the APP family in the adult cerebral cortex. *Sci Rep* **2022,** 12, (1), 66.

41. Sabo, S. L.; Ikin, A. F.; Buxbaum, J. D.; Greengard, P., The Alzheimer amyloid precursor protein (APP) and FE65, an APP-binding protein, regulate cell movement. *J Cell Biol* **2001,** 153, (7), 1403-14.

42. Cao, X.; Sudhof, T. C., A transcriptionally [correction of transcriptively] active complex of APP with Fe65 and histone acetyltransferase Tip60. *Science* **2001,** 293, (5527), 115-20.

43. Chen, G. F.; Xu, T. H.; Yan, Y.; Zhou, Y. R.; Jiang, Y.; Melcher, K.; Xu, H. E., Amyloid beta: structure, biology and structure-based therapeutic development. *Acta Pharmacol Sin* **2017,** 38, (9), 1205-1235.

44. Konietzko, U., AICD nuclear signaling and its possible contribution to Alzheimer's disease. *Curr Alzheimer Res* **2012,** 9, (2), 200-16.

45. Bukhari, H.; Glotzbach, A.; Kolbe, K.; Leonhardt, G.; Loosse, C.; Muller, T., Small things matter: Implications of APP intracellular domain AICD nuclear signaling in the progression and pathogenesis of Alzheimer's disease. *Prog Neurobiol* **2017,** 156, 189-213.

46. Benosman, S.; Meng, X.; Von Grabowiecki, Y.; Palamiuc, L.; Hritcu, L.; Gross, I.; Mellitzer, G.; Taya, Y.; Loeffler, J. P.; Gaiddon, C., Complex regulation of p73 isoforms after alteration of amyloid precursor polypeptide (APP) function and DNA damage in neurons. *J Biol Chem* **2011,** 286, (50), 43013-25.
47. Fuentes, N.; Silveyra, P., Estrogen receptor signaling mechanisms. *Adv Protein Chem Struct Biol* **2019,** 116, 135-170.
48. Wittmann, B. M.; Sherk, A.; McDonnell, D. P., Definition of functionally important mechanistic differences among selective estrogen receptor down-regulators. *Cancer Res* **2007,** 67, (19), 9549-60.
49. Frasor, J.; Stossi, F.; Danes, J. M.; Komm, B.; Lyttle, C. R.; Katzenellenbogen, B. S., Selective estrogen receptor modulators: discrimination of agonistic versus antagonistic activities by gene expression profiling in breast cancer cells. *Cancer Res* **2004,** 64, (4), 1522-33.
50. Bai, Y.; Giguere, V., Isoform-selective interactions between estrogen receptors and steroid receptor coactivators promoted by estradiol and ErbB-2 signaling in living cells. *Mol Endocrinol* **2003,** 17, (4), 589-99.
51. Ogawa, S.; Inoue, S.; Watanabe, T.; Hiroi, H.; Orimo, A.; Hosoi, T.; Ouchi, Y.; Muramatsu, M., The complete primary structure of human estrogen receptor beta (hER beta) and its heterodimerization with ER alpha in vivo and in vitro. *Biochem Biophys Res Commun* **1998,** 243, (1), 122-6.
52. Wu, Q.; Burghardt, R.; Safe, S., Vitamin D-interacting protein 205 (DRIP205) coactivation of estrogen receptor alpha (ERalpha) involves multiple domains of both proteins. *J Biol Chem* **2004,** 279, (51), 53602-12.
53. Yun, J.; Son, C. H.; Um, S. J.; Kwon, H. C.; Lee, K. E.; Choi, P. J.; Roh, M. S., A different TRAP220 expression in distinct histologic subtypes of lung adenocarcinoma and the prognostic significance. *Lung Cancer* **2011,** 71, (3), 312-8.
54. Fujita, T.; Kobayashi, Y.; Wada, O.; Tateishi, Y.; Kitada, L.; Yamamoto, Y.; Takashima, H.; Murayama, A.; Yano, T.; Baba, T.; Kato, S.; Kawabe, Y.; Yanagisawa, J., Full activation of estrogen receptor alpha activation function-1 induces proliferation of breast cancer cells. *J Biol Chem* **2003,** 278, (29), 26704-14.
55. Burakov, D.; Wong, C. W.; Rachez, C.; Cheskis, B. J.; Freedman, L. P., Functional interactions between the estrogen receptor and DRIP205, a subunit of the heteromeric DRIP coactivator complex. *J Biol Chem* **2000,** 275, (27), 20928-34.
56. Sauve, F.; McBroom, L. D.; Gallant, J.; Moraitis, A. N.; Labrie, F.; Giguere, V., CIA, a novel estrogen receptor coactivator with a bifunctional nuclear receptor interacting determinant. *Mol Cell Biol* **2001,** 21, (1), 343-53.
57. Yue, X.; Izcue, A.; Borggrefe, T., Essential role of Mediator subunit Med1 in invariant natural killer T-cell development. *Proc Natl Acad Sci U S A* **2011,** 108, (41), 17105-10.
58. Oda, Y.; Chalkley, R. J.; Burlingame, A. L.; Bikle, D. D., The transcriptional coactivator DRIP/mediator complex is involved in vitamin D receptor function and regulates keratinocyte proliferation and differentiation. *J Invest Dermatol* **2010,** 130, (10), 2377-88.
59. Jiang, C.; Ito, M.; Piening, V.; Bruck, K.; Roeder, R. G.; Xiao, H., TIP30 interacts with an estrogen receptor alpha-interacting coactivator CIA and regulates c-myc transcription. *J Biol Chem* **2004,** 279, (26), 27781-9.
60. Hoffmeister, H.; Fuchs, A.; Erdel, F.; Pinz, S.; Grobner-Ferreira, R.; Bruckmann, A.; Deutzmann, R.; Schwartz, U.; Maldonado, R.; Huber, C.; Dendorfer, A. S.; Rippe, K.; Langst, G., CHD3 and CHD4 form distinct NuRD complexes with different yet overlapping functionality. *Nucleic Acids Res* **2017,** 45, (18), 10534-10554.
61. Tang, J.; Luo, Y.; Tian, Z.; Liao, X.; Cui, Q.; Yang, Q.; Wu, G., TRIM11 promotes breast cancer cell proliferation by stabilizing estrogen receptor alpha. *Neoplasia* **2020,** 22, (9), 343-351.
62. Huttlin, E. L.; Bruckner, R. J.; Navarrete-Perea, J.; Cannon, J. R.; Baltier, K.; Gebreab, F.; Gygi, M. P.; Thornock, A.; Zarraga, G.; Tam, S.; Szpyt, J.; Gassaway, B. M.; Panov, A.; Parzen, H.; Fu, S.; Golbazi, A.; Maenpaa, E.; Stricker, K.; Guha Thakurta, S.; Zhang, T.; Rad, R.; Pan, J.;

Nusinow, D. P.; Paulo, J. A.; Schweppe, D. K.; Vaites, L. P.; Harper, J. W.; Gygi, S. P., Dual proteome-scale networks reveal cell-specific remodeling of the human interactome. *Cell* **2021,** 184, (11), 3022-3040 e28.
63. Keenen, M. M.; Kim, S., Tumor suppressor ING4 inhibits estrogen receptor activity in breast cancer cells. *Breast Cancer (Dove Med Press)* **2016,** 8, 211-221.
64. Norris, J. D.; Fan, D.; Sherk, A.; McDonnell, D. P., A negative coregulator for the human ER. *Mol Endocrinol* **2002,** 16, (3), 459-68.
65. Rolland, T.; Tasan, M.; Charloteaux, B.; Pevzner, S. J.; Zhong, Q.; Sahni, N.; Yi, S.; Lemmens, I.; Fontanillo, C.; Mosca, R.; Kamburov, A.; Ghiassian, S. D.; Yang, X.; Ghamsari, L.; Balcha, D.; Begg, B. E.; Braun, P.; Brehme, M.; Broly, M. P.; Carvunis, A. R.; Convery-Zupan, D.; Corominas, R.; Coulombe-Huntington, J.; Dann, E.; Dreze, M.; Dricot, A.; Fan, C.; Franzosa, E.; Gebreab, F.; Gutierrez, B. J.; Hardy, M. F.; Jin, M.; Kang, S.; Kiros, R.; Lin, G. N.; Luck, K.; MacWilliams, A.; Menche, J.; Murray, R. R.; Palagi, A.; Poulin, M. M.; Rambout, X.; Rasla, J.; Reichert, P.; Romero, V.; Ruyssinck, E.; Sahalie, J. M.; Scholz, A.; Shah, A. A.; Sharma, A.; Shen, Y.; Spirohn, K.; Tam, S.; Tejeda, A. O.; Trigg, S. A.; Twizere, J. C.; Vega, K.; Walsh, J.; Cusick, M. E.; Xia, Y.; Barabasi, A. L.; Iakoucheva, L. M.; Aloy, P.; De Las Rivas, J.; Tavernier, J.; Calderwood, M. A.; Hill, D. E.; Hao, T.; Roth, F. P.; Vidal, M., A proteome-scale map of the human interactome network. *Cell* **2014,** 159, (5), 1212-1226.
66. Adachi, J.; Kumar, C.; Zhang, Y.; Olsen, J. V.; Mann, M., The human urinary proteome contains more than 1500 proteins, including a large proportion of membrane proteins. *Genome Biol* **2006,** 7, (9), R80.
67. Notas, G.; Kampa, M.; Pelekanou, V.; Troullinaki, M.; Jacquot, Y.; Leclercq, G.; Castanas, E., Whole transcriptome analysis of the ERalpha synthetic fragment P295-T311 (ERalpha17p) identifies specific ERalpha-isoform (ERalpha, ERalpha36)-dependent and -independent actions in breast cancer cells. *Mol Oncol* **2013,** 7, (3), 595-610.
68. Stender, J. D.; Kim, K.; Charn, T. H.; Komm, B.; Chang, K. C.; Kraus, W. L.; Benner, C.; Glass, C. K.; Katzenellenbogen, B. S., Genome-wide analysis of estrogen receptor alpha DNA binding and tethering mechanisms identifies Runx1 as a novel tethering factor in receptor-mediated transcriptional activation. *Mol Cell Biol* **2010,** 30, (16), 3943-55.
69. Ye, Y.; Xiao, Y.; Wang, W.; Yearsley, K.; Gao, J. X.; Shetuni, B.; Barsky, S. H., ERalpha signaling through slug regulates E-cadherin and EMT. *Oncogene* **2010,** 29, (10), 1451-62.
70. Liu, X. F.; Bagchi, M. K., Recruitment of distinct chromatin-modifying complexes by tamoxifen-complexed estrogen receptor at natural target gene promoters in vivo. *J Biol Chem* **2004,** 279, (15), 15050-8.
71. Jin, L.; Williamson, A.; Banerjee, S.; Philipp, I.; Rape, M., Mechanism of ubiquitin-chain formation by the human anaphase-promoting complex. *Cell* **2008,** 133, (4), 653-65.
72. Bott, L. C.; Salomons, F. A.; Maric, D.; Liu, Y.; Merry, D.; Fischbeck, K. H.; Dantuma, N. P., The polyglutamine-expanded androgen receptor responsible for spinal and bulbar muscular atrophy inhibits the APC/C(Cdh1) ubiquitin ligase complex. *Sci Rep* **2016,** 6, 27703.
73. Giurato, G.; Nassa, G.; Salvati, A.; Alexandrova, E.; Rizzo, F.; Nyman, T. A.; Weisz, A.; Tarallo, R., Quantitative mapping of RNA-mediated nuclear estrogen receptor beta interactome in human breast cancer cells. *Sci Data* **2018,** 5, 180031.
74. Shi, Y.; Xu, X.; Zhang, Q.; Fu, G.; Mo, Z.; Wang, G. S.; Kishi, S.; Yang, X. L., tRNA synthetase counteracts c-Myc to develop functional vasculature. *Elife* **2014,** 3, e02349.
75. Guo, Q. M.; Malek, R. L.; Kim, S.; Chiao, C.; He, M.; Ruffy, M.; Sanka, K.; Lee, N. H.; Dang, C. V.; Liu, E. T., Identification of c-myc responsive genes using rat cDNA microarray. *Cancer Res* **2000,** 60, (21), 5922-8.
76. Hsu, T. Y.; Simon, L. M.; Neill, N. J.; Marcotte, R.; Sayad, A.; Bland, C. S.; Echeverria, G. V.; Sun, T.; Kurley, S. J.; Tyagi, S.; Karlin, K. L.; Dominguez-Vidana, R.; Hartman, J. D.; Renwick, A.; Scorsone, K.; Bernardi, R. J.; Skinner, S. O.; Jain, A.; Orellana, M.; Lagisetti, C.; Golding, I.; Jung, S. Y.; Neilson, J. R.; Zhang, X. H.; Cooper, T. A.; Webb, T. R.; Neel, B. G.; Shaw, C. A.;

Westbrook, T. F., The spliceosome is a therapeutic vulnerability in MYC-driven cancer. *Nature* **2015,** 525, (7569), 384-8.
77. Zelaya, M. V.; Perez-Valderrama, E.; de Morentin, X. M.; Tunon, T.; Ferrer, I.; Luquin, M. R.; Fernandez-Irigoyen, J.; Santamaria, E., Olfactory bulb proteome dynamics during the progression of sporadic Alzheimer's disease: identification of common and distinct olfactory targets across Alzheimer-related co-pathologies. *Oncotarget* **2015,** 6, (37), 39437-56.
78. O'Connell, B. C.; Cheung, A. F.; Simkevich, C. P.; Tam, W.; Ren, X.; Mateyak, M. K.; Sedivy, J. M., A large scale genetic analysis of c-Myc-regulated gene expression patterns. *J Biol Chem* **2003,** 278, (14), 12563-73.
79. Heidelberger, J. B.; Voigt, A.; Borisova, M. E.; Petrosino, G.; Ruf, S.; Wagner, S. A.; Beli, P., Proteomic profiling of VCP substrates links VCP to K6-linked ubiquitylation and c-Myc function. *EMBO Rep* **2018,** 19, (4).
80. Uhlen, M.; Fagerberg, L.; Hallstrom, B. M.; Lindskog, C.; Oksvold, P.; Mardinoglu, A.; Sivertsson, A.; Kampf, C.; Sjostedt, E.; Asplund, A.; Olsson, I.; Edlund, K.; Lundberg, E.; Navani, S.; Szigyarto, C. A.; Odeberg, J.; Djureinovic, D.; Takanen, J. O.; Hober, S.; Alm, T.; Edqvist, P. H.; Berling, H.; Tegel, H.; Mulder, J.; Rockberg, J.; Nilsson, P.; Schwenk, J. M.; Hamsten, M.; von Feilitzen, K.; Forsberg, M.; Persson, L.; Johansson, F.; Zwahlen, M.; von Heijne, G.; Nielsen, J.; Ponten, F., Proteomics. Tissue-based map of the human proteome. *Science* **2015,** 347, (6220), 1260419.
81. Mohammed, H.; D'Santos, C.; Serandour, A. A.; Ali, H. R.; Brown, G. D.; Atkins, A.; Rueda, O. M.; Holmes, K. A.; Theodorou, V.; Robinson, J. L.; Zwart, W.; Saadi, A.; Ross-Innes, C. S.; Chin, S. F.; Menon, S.; Stingl, J.; Palmieri, C.; Caldas, C.; Carroll, J. S., Endogenous purification reveals GREB1 as a key estrogen receptor regulatory factor. *Cell Rep* **2013,** 3, (2), 342-9.
82. Vermeulen, M.; Eberl, H. C.; Matarese, F.; Marks, H.; Denissov, S.; Butter, F.; Lee, K. K.; Olsen, J. V.; Hyman, A. A.; Stunnenberg, H. G.; Mann, M., Quantitative interaction proteomics and genome-wide profiling of epigenetic histone marks and their readers. *Cell* **2010,** 142, (6), 967-80.
83. Marques, J. G.; Gryder, B. E.; Pavlovic, B.; Chung, Y.; Ngo, Q. A.; Frommelt, F.; Gstaiger, M.; Song, Y.; Benischke, K.; Laubscher, D.; Wachtel, M.; Khan, J.; Schafer, B. W., NuRD subunit CHD4 regulates super-enhancer accessibility in rhabdomyosarcoma and represents a general tumor dependency. *Elife* **2020,** 9.
84. Sowa, M. E.; Bennett, E. J.; Gygi, S. P.; Harper, J. W., Defining the human deubiquitinating enzyme interaction landscape. *Cell* **2009,** 138, (2), 389-403.
85. He, L.; Liu, X.; Yang, J.; Li, W.; Liu, S.; Liu, X.; Yang, Z.; Ren, J.; Wang, Y.; Shan, L.; Guan, C.; Pei, F.; Lei, L.; Zhang, Y.; Yi, X.; Yang, X.; Liang, J.; Liu, R.; Sun, L.; Shang, Y., Imbalance of the reciprocally inhibitory loop between the ubiquitin-specific protease USP43 and EGFR/PI3K/AKT drives breast carcinogenesis. *Cell Res* **2018,** 28, (9), 934-951.
86. Nassa, G.; Giurato, G.; Salvati, A.; Gigantino, V.; Pecoraro, G.; Lamberti, J.; Rizzo, F.; Nyman, T. A.; Tarallo, R.; Weisz, A., The RNA-mediated estrogen receptor alpha interactome of hormone-dependent human breast cancer cell nuclei. *Sci Data* **2019,** 6, (1), 173.
87. Shi, B.; Liang, J.; Yang, X.; Wang, Y.; Zhao, Y.; Wu, H.; Sun, L.; Zhang, Y.; Chen, Y.; Li, R.; Zhang, Y.; Hong, M.; Shang, Y., Integration of estrogen and Wnt signaling circuits by the polycomb group protein EZH2 in breast cancer cells. *Mol Cell Biol* **2007,** 27, (14), 5105-19.
88. Shin, E. M.; Huynh, V. T.; Neja, S. A.; Liu, C. Y.; Raju, A.; Tan, K.; Tan, N. S.; Gunaratne, J.; Bi, X.; Iyer, L. M.; Aravind, L.; Tergaonkar, V., GREB1: An evolutionarily conserved protein with a glycosyltransferase domain links ERalpha glycosylation and stability to cancer. *Sci Adv* **2021,** 7, (12).
89. Tarallo, R.; Bamundo, A.; Nassa, G.; Nola, E.; Paris, O.; Ambrosino, C.; Facchiano, A.; Baumann, M.; Nyman, T. A.; Weisz, A., Identification of proteins associated with ligand-activated estrogen receptor alpha in human breast cancer cell nuclei by tandem affinity purification and nano LC-MS/MS. *Proteomics* **2011,** 11, (1), 172-9.

90. Iorns, E.; Martens-de Kemp, S. R.; Lord, C. J.; Ashworth, A., CRK7 modifies the MAPK pathway and influences the response to endocrine therapy. *Carcinogenesis* **2009,** 30, (10), 1696-701.
91. Davidson, L.; Muniz, L.; West, S., 3' end formation of pre-mRNA and phosphorylation of Ser2 on the RNA polymerase II CTD are reciprocally coupled in human cells. *Genes Dev* **2014,** 28, (4), 342-56.
92. Toyoshima, M.; Howie, H. L.; Imakura, M.; Walsh, R. M.; Annis, J. E.; Chang, A. N.; Frazier, J.; Chau, B. N.; Loboda, A.; Linsley, P. S.; Cleary, M. A.; Park, J. R.; Grandori, C., Functional genomics identifies therapeutic targets for MYC-driven cancer. *Proc Natl Acad Sci U S A* **2012,** 109, (24), 9545-50.
93. Ruepp, M. D.; Aringhieri, C.; Vivarelli, S.; Cardinale, S.; Paro, S.; Schumperli, D.; Barabino, S. M., Mammalian pre-mRNA 3' end processing factor CF I m 68 functions in mRNA export. *Mol Biol Cell* **2009,** 20, (24), 5211-23.
94. Du, L.; Fakih, M. G.; Rosen, S. T.; Chen, Y., SUMOylation of E2F1 Regulates Expression of EZH2. *Cancer Res* **2020,** 80, (19), 4212-4223.
95. Margueron, R.; Li, G.; Sarma, K.; Blais, A.; Zavadil, J.; Woodcock, C. L.; Dynlacht, B. D.; Reinberg, D., Ezh1 and Ezh2 maintain repressive chromatin through different mechanisms. *Mol Cell* **2008,** 32, (4), 503-18.
96. Oliviero, G.; Brien, G. L.; Waston, A.; Streubel, G.; Jerman, E.; Andrews, D.; Doyle, B.; Munawar, N.; Wynne, K.; Crean, J.; Bracken, A. P.; Cagney, G., Dynamic Protein Interactions of the Polycomb Repressive Complex 2 during Differentiation of Pluripotent Cells. *Mol Cell Proteomics* **2016,** 15, (11), 3450-3460.
97. Joshi, P.; Greco, T. M.; Guise, A. J.; Luo, Y.; Yu, F.; Nesvizhskii, A. I.; Cristea, I. M., The functional interactome landscape of the human histone deacetylase family. *Mol Syst Biol* **2013,** 9, 672.
98. Fasci, D.; van Ingen, H.; Scheltema, R. A.; Heck, A. J. R., Histone Interaction Landscapes Visualized by Crosslinking Mass Spectrometry in Intact Cell Nuclei. *Mol Cell Proteomics* **2018,** 17, (10), 2018-2033.
99. Pichler, G.; Jack, A.; Wolf, P.; Hake, S. B., Versatile toolbox for high throughput biochemical and functional studies with fluorescent fusion proteins. *PLoS One* **2012,** 7, (5), e36967.
100. Varier, R. A.; Outchkourov, N. S.; de Graaf, P.; van Schaik, F. M.; Ensing, H. J.; Wang, F.; Higgins, J. M.; Kops, G. J.; Timmers, H. T., A phospho/methyl switch at histone H3 regulates TFIID association with mitotic chromosomes. *EMBO J* **2010,** 29, (23), 3967-78.
101. Campos, E. I.; Fillingham, J.; Li, G.; Zheng, H.; Voigt, P.; Kuo, W. H.; Seepany, H.; Gao, Z.; Day, L. A.; Greenblatt, J. F.; Reinberg, D., The program for processing newly synthesized histones H3.1 and H4. *Nat Struct Mol Biol* **2010,** 17, (11), 1343-51.
102. Twells, R. C.; Metzker, M. L.; Brown, S. D.; Cox, R.; Garey, C.; Hammond, H.; Hey, P. J.; Levy, E.; Nakagawa, Y.; Philips, M. S.; Todd, J. A.; Hess, J. F., The sequence and gene characterization of a 400-kb candidate region for IDDM4 on chromosome 11q13. *Genomics* **2001,** 72, (3), 231-42.
103. Stefansson, B.; Brautigan, D. L., Protein phosphatase 6 subunit with conserved Sit4-associated protein domain targets IkappaBepsilon. *J Biol Chem* **2006,** 281, (32), 22624-34.
104. Couzens, A. L.; Knight, J. D.; Kean, M. J.; Teo, G.; Weiss, A.; Dunham, W. H.; Lin, Z. Y.; Bagshaw, R. D.; Sicheri, F.; Pawson, T.; Wrana, J. L.; Choi, H.; Gingras, A. C., Protein interaction network of the mammalian Hippo pathway reveals mechanisms of kinase-phosphatase interactions. *Sci Signal* **2013,** 6, (302), rs15.
105. Zhang, Y.; Yao, Y.; Wang, Z.; Lu, D.; Zhang, Y.; Adetula, A. A.; Liu, S.; Zhu, M.; Yang, Y.; Fan, X.; Chen, M.; Tang, Y.; Chen, Y.; Liu, Y.; Yi, G.; Tang, Z., MiR-743a-5p regulates differentiation of myoblast by targeting Mob1b in skeletal muscle development and regeneration. *Genes Dis* **2022,** 9, (4), 1038-1048.
106. Sun, X. X.; He, X.; Yin, L.; Komada, M.; Sears, R. C.; Dai, M. S., The nucleolar ubiquitin-specific protease USP36 deubiquitinates and stabilizes c-Myc. *Proc Natl Acad Sci U S A* **2015,** 112, (12), 3734-9.

107. Hsiao, J. J.; Smits, M. M.; Ng, B. H.; Lee, J.; Wright, M. E., Discovery Proteomics Identifies a Molecular Link between the Coatomer Protein Complex I and Androgen Receptor-dependent Transcription. *J Biol Chem* **2016,** 291, (36), 18818-42.
108. Uramoto, H.; Izumi, H.; Nagatani, G.; Ohmori, H.; Nagasue, N.; Ise, T.; Yoshida, T.; Yasumoto, K.; Kohno, K., Physical interaction of tumour suppressor p53/p73 with CCAAT-binding transcription factor 2 (CTF2) and differential regulation of human high-mobility group 1 (HMG1) gene expression. *Biochem J* **2003,** 371, (Pt 2), 301-10.
109. Flores, E. R.; Tsai, K. Y.; Crowley, D.; Sengupta, S.; Yang, A.; McKeon, F.; Jacks, T., p63 and p73 are required for p53-dependent apoptosis in response to DNA damage. *Nature* **2002,** 416, (6880), 560-4.
110. Scian, M. J.; Carchman, E. H.; Mohanraj, L.; Stagliano, K. E.; Anderson, M. A.; Deb, D.; Crane, B. M.; Kiyono, T.; Windle, B.; Deb, S. P.; Deb, S., Wild-type p53 and p73 negatively regulate expression of proliferation related genes. *Oncogene* **2008,** 27, (18), 2583-93.
111. Odom, D. T.; Zizlsperger, N.; Gordon, D. B.; Bell, G. W.; Rinaldi, N. J.; Murray, H. L.; Volkert, T. L.; Schreiber, J.; Rolfe, P. A.; Gifford, D. K.; Fraenkel, E.; Bell, G. I.; Young, R. A., Control of pancreas and liver gene expression by HNF transcription factors. *Science* **2004,** 303, (5662), 1378-81.
112. Li, M.; Messaddeq, N.; Teletin, M.; Pasquali, J. L.; Metzger, D.; Chambon, P., Retinoid X receptor ablation in adult mouse keratinocytes generates an atopic dermatitis triggered by thymic stromal lymphopoietin. *Proc Natl Acad Sci U S A* **2005,** 102, (41), 14795-800.
113. Kawahara, K.; Suenobu, M.; Ohtsuka, H.; Kuniyasu, A.; Sugimoto, Y.; Nakagomi, M.; Fukasawa, H.; Shudo, K.; Nakayama, H., Cooperative therapeutic action of retinoic acid receptor and retinoid x receptor agonists in a mouse model of Alzheimer's disease. *J Alzheimers Dis* **2014,** 42, (2), 587-605.
114. Lee, H. P.; Casadesus, G.; Zhu, X.; Lee, H. G.; Perry, G.; Smith, M. A.; Gustaw-Rothenberg, K.; Lerner, A., All-trans retinoic acid as a novel therapeutic strategy for Alzheimer's disease. *Expert Rev Neurother* **2009,** 9, (11), 1615-21.
115. Vinayagam, A.; Stelzl, U.; Foulle, R.; Plassmann, S.; Zenkner, M.; Timm, J.; Assmus, H. E.; Andrade-Navarro, M. A.; Wanker, E. E., A directed protein interaction network for investigating intracellular signal transduction. *Sci Signal* **2011,** 4, (189), rs8.
116. Monje, P.; Zanello, S.; Holick, M.; Boland, R., Differential cellular localization of estrogen receptor alpha in uterine and mammary cells. *Mol Cell Endocrinol* **2001,** 181, (1-2), 117-29.
117. Zhang, Y.; Li, C.; Li, W.; Zhao, Y., Estrogen regulation of human with-no-lysine (K) kinase-4 gene expression involves AP-1 transcription factor. *Mol Cell Endocrinol* **2011,** 332, (1-2), 140-8.
118. Heise, C. J.; Xu, B. E.; Deaton, S. L.; Cha, S. K.; Cheng, C. J.; Earnest, S.; Sengupta, S.; Juang, Y. C.; Stippec, S.; Xu, Y.; Zhao, Y.; Huang, C. L.; Cobb, M. H., Serum and glucocorticoid-induced kinase (SGK) 1 and the epithelial sodium channel are regulated by multiple with no lysine (WNK) family members. *J Biol Chem* **2010,** 285, (33), 25161-7.
119. Tu, J.; Zhang, B.; Fang, G.; Chang, W.; Zhao, Y., Neddylation-mediated Nedd4-2 activation regulates ubiquitination modification of renal NBCe1. *Exp Cell Res* **2020,** 390, (2), 111958.
120. Su, N.; Choi, H. P.; Wang, F.; Su, H.; Fei, Z.; Yang, J. H.; Azadzoi, K. M., Quantitative Proteomic Analysis of Differentially Expressed Proteins and Downstream Signaling Pathways in Chronic Bladder Ischemia. *J Urol* **2016,** 195, (2), 515-23.
121. Chen, L. C.; Kuo, H. C., Pathophysiology of refractory overactive bladder. *Low Urin Tract Symptoms* **2019,** 11, (4), 177-181.
122. Handlogten, M. E.; Osis, G.; Lee, H. W.; Romero, M. F.; Verlander, J. W.; Weiner, I. D., NBCe1 expression is required for normal renal ammonia metabolism. *Am J Physiol Renal Physiol* **2015,** 309, (7), F658-66.
123. Sarvari, M.; Hrabovszky, E.; Kallo, I.; Galamb, O.; Solymosi, N.; Liko, I.; Molnar, B.; Tihanyi, K.; Szombathelyi, Z.; Liposits, Z., Gene expression profiling identifies key estradiol targets in the frontal cortex of the rat. *Endocrinology* **2010,** 151, (3), 1161-76.

124. Comstock, C. E. S.; Augello, M. A.; Schiewer, M. J.; Karch, J.; Burd, C. J.; Ertel, A.; Knudsen, E. S.; Jessen, W. J.; Aronow, B. J.; Knudsen, K. E., Cyclin D1 is a selective modifier of androgen-dependent signaling and androgen receptor function. *J Biol Chem* **2011,** 286, (10), 8117-8127.
125. Shi, H.; Belbin, O.; Medway, C.; Brown, K.; Kalsheker, N.; Carrasquillo, M.; Proitsi, P.; Powell, J.; Lovestone, S.; Goate, A.; Younkin, S.; Passmore, P.; Genetic; Environmental Risk for Alzheimer's Disease, C.; Morgan, K.; Alzheimer's Research, U. K. C., Genetic variants influencing human aging from late-onset Alzheimer's disease (LOAD) genome-wide association studies (GWAS). *Neurobiol Aging* **2012,** 33, (8), 1849 e5-18.
126. Lee, E.; Giovanello, K. S.; Saykin, A. J.; Xie, F.; Kong, D.; Wang, Y.; Yang, L.; Ibrahim, J. G.; Doraiswamy, P. M.; Zhu, H., Single-nucleotide polymorphisms are associated with cognitive decline at Alzheimer's disease conversion within mild cognitive impairment patients. *Alzheimers Dement (Amst)* **2017,** 8, 86-95.
127. Yamazaki, S.; Harashima, S.; Sakaguchi, M.; Mihara, K., Identification and functional characterization of yeast zeta-COP. *J Biochem* **1997,** 121, (1), 8-14.
128. Kennedy, S. A.; Jarboui, M. A.; Srihari, S.; Raso, C.; Bryan, K.; Dernayka, L.; Charitou, T.; Bernal-Llinares, M.; Herrera-Montavez, C.; Krstic, A.; Matallanas, D.; Kotlyar, M.; Jurisica, I.; Curak, J.; Wong, V.; Stagljar, I.; LeBihan, T.; Imrie, L.; Pillai, P.; Lynn, M. A.; Fasterius, E.; Al-Khalili Szigyarto, C.; Breen, J.; Kiel, C.; Serrano, L.; Rauch, N.; Rukhlenko, O.; Kholodenko, B. N.; Iglesias-Martinez, L. F.; Ryan, C. J.; Pilkington, R.; Cammareri, P.; Sansom, O.; Shave, S.; Auer, M.; Horn, N.; Klose, F.; Ueffing, M.; Boldt, K.; Lynn, D. J.; Kolch, W., Extensive rewiring of the EGFR network in colorectal cancer cells expressing transforming levels of KRAS(G13D). *Nat Commun* **2020,** 11, (1), 499.
129. Capalbo, L.; Bassi, Z. I.; Geymonat, M.; Todesca, S.; Copoiu, L.; Enright, A. J.; Callaini, G.; Riparbelli, M. G.; Yu, L.; Choudhary, J. S.; Ferrero, E.; Wheatley, S.; Douglas, M. E.; Mishima, M.; D'Avino, P. P., The midbody interactome reveals unexpected roles for PP1 phosphatases in cytokinesis. *Nat Commun* **2019,** 10, (1), 4513.
130. Nakajima, M.; Mizumoto, S.; Miyake, N.; Kogawa, R.; Iida, A.; Ito, H.; Kitoh, H.; Hirayama, A.; Mitsubuchi, H.; Miyazaki, O.; Kosaki, R.; Horikawa, R.; Lai, A.; Mendoza-Londono, R.; Dupuis, L.; Chitayat, D.; Howard, A.; Leal, G. F.; Cavalcanti, D.; Tsurusaki, Y.; Saitsu, H.; Watanabe, S.; Lausch, E.; Unger, S.; Bonafe, L.; Ohashi, H.; Superti-Furga, A.; Matsumoto, N.; Sugahara, K.; Nishimura, G.; Ikegawa, S., Mutations in B3GALT6, which encodes a glycosaminoglycan linker region enzyme, cause a spectrum of skeletal and connective tissue disorders. *Am J Hum Genet* **2013,** 92, (6), 927-34.
131. Bai, X.; Zhou, D.; Brown, J. R.; Crawford, B. E.; Hennet, T.; Esko, J. D., Biosynthesis of the linkage region of glycosaminoglycans: cloning and activity of galactosyltransferase II, the sixth member of the beta 1,3-galactosyltransferase family (beta 3GalT6). *J Biol Chem* **2001,** 276, (51), 48189-95.
132. Nell, H. J.; Au, J. L.; Giordano, C. R.; Terlecky, S. R.; Walton, P. A.; Whitehead, S. N.; Cechetto, D. F., Targeted Antioxidant, Catalase-SKL, Reduces Beta-Amyloid Toxicity in the Rat Brain. *Brain Pathol* **2017,** 27, (1), 86-94.
133. Antonicka, H.; Lin, Z. Y.; Janer, A.; Aaltonen, M. J.; Weraarpachai, W.; Gingras, A. C.; Shoubridge, E. A., A High-Density Human Mitochondrial Proximity Interaction Network. *Cell Metab* **2020,** 32, (3), 479-497 e9.
134. Lyons, A.; Downer, E. J.; Costello, D. A.; Murphy, N.; Lynch, M. A., Dok2 mediates the CD200Fc attenuation of Abeta-induced changes in glia. *J Neuroinflammation* **2012,** 9, 107.
135. Lu, L.; Hu, S.; Wei, R.; Qiu, X.; Lu, K.; Fu, Y.; Li, H.; Xing, G.; Li, D.; Peng, R.; He, F.; Zhang, L., The HECT type ubiquitin ligase NEDL2 is degraded by anaphase-promoting complex/cyclosome (APC/C)-Cdh1, and its tight regulation maintains the metaphase to anaphase transition. *J Biol Chem* **2013,** 288, (50), 35637-50.

136. Miyazaki, K.; Ozaki, T.; Kato, C.; Hanamoto, T.; Fujita, T.; Irino, S.; Watanabe, K.; Nakagawa, T.; Nakagawara, A., A novel HECT-type E3 ubiquitin ligase, NEDL2, stabilizes p73 and enhances its transcriptional activity. *Biochem Biophys Res Commun* **2003,** 308, (1), 106-13.
137. Taipale, M.; Tucker, G.; Peng, J.; Krykbaeva, I.; Lin, Z. Y.; Larsen, B.; Choi, H.; Berger, B.; Gingras, A. C.; Lindquist, S., A quantitative chaperone interaction network reveals the architecture of cellular protein homeostasis pathways. *Cell* **2014,** 158, (2), 434-448.
138. Silva, F. P.; Hamamoto, R.; Nakamura, Y.; Furukawa, Y., WDRPUH, a novel WD-repeat-containing protein, is highly expressed in human hepatocellular carcinoma and involved in cell proliferation. *Neoplasia* **2005,** 7, (4), 348-55.
139. Virok, D. P.; Simon, D.; Bozso, Z.; Rajko, R.; Datki, Z.; Balint, E.; Szegedi, V.; Janaky, T.; Penke, B.; Fulop, L., Protein array based interactome analysis of amyloid-beta indicates an inhibition of protein translation. *J Proteome Res* **2011,** 10, (4), 1538-47.
140. Huttlin, E. L.; Bruckner, R. J.; Paulo, J. A.; Cannon, J. R.; Ting, L.; Baltier, K.; Colby, G.; Gebreab, F.; Gygi, M. P.; Parzen, H.; Szpyt, J.; Tam, S.; Zarraga, G.; Pontano-Vaites, L.; Swarup, S.; White, A. E.; Schweppe, D. K.; Rad, R.; Erickson, B. K.; Obar, R. A.; Guruharsha, K. G.; Li, K.; Artavanis-Tsakonas, S.; Gygi, S. P.; Harper, J. W., Architecture of the human interactome defines protein communities and disease networks. *Nature* **2017,** 545, (7655), 505-509.
141. Arimoto, K. I.; Lochte, S.; Stoner, S. A.; Burkart, C.; Zhang, Y.; Miyauchi, S.; Wilmes, S.; Fan, J. B.; Heinisch, J. J.; Li, Z.; Yan, M.; Pellegrini, S.; Colland, F.; Piehler, J.; Zhang, D. E., STAT2 is an essential adaptor in USP18-mediated suppression of type I interferon signaling. *Nat Struct Mol Biol* **2017,** 24, (3), 279-289.
142. Huttlin, E. L.; Ting, L.; Bruckner, R. J.; Gebreab, F.; Gygi, M. P.; Szpyt, J.; Tam, S.; Zarraga, G.; Colby, G.; Baltier, K.; Dong, R.; Guarani, V.; Vaites, L. P.; Ordureau, A.; Rad, R.; Erickson, B. K.; Wuhr, M.; Chick, J.; Zhai, B.; Kolippakkam, D.; Mintseris, J.; Obar, R. A.; Harris, T.; Artavanis-Tsakonas, S.; Sowa, M. E.; De Camilli, P.; Paulo, J. A.; Harper, J. W.; Gygi, S. P., The BioPlex Network: A Systematic Exploration of the Human Interactome. *Cell* **2015,** 162, (2), 425-440.
143. Hein, M. Y.; Hubner, N. C.; Poser, I.; Cox, J.; Nagaraj, N.; Toyoda, Y.; Gak, I. A.; Weisswange, I.; Mansfeld, J.; Buchholz, F.; Hyman, A. A.; Mann, M., A human interactome in three quantitative dimensions organized by stoichiometries and abundances. *Cell* **2015,** 163, (3), 712-23.
144. Wright, S.; Malinin, N. L.; Powell, K. A.; Yednock, T.; Rydel, R. E.; Griswold-Prenner, I., Alpha2beta1 and alphaVbeta1 integrin signaling pathways mediate amyloid-beta-induced neurotoxicity. *Neurobiol Aging* **2007,** 28, (2), 226-37.
145. Anton, Z.; Betin, V. M. S.; Simonetti, B.; Traer, C. J.; Attar, N.; Cullen, P. J.; Lane, J. D., A heterodimeric SNX4--SNX7 SNX-BAR autophagy complex coordinates ATG9A trafficking for efficient autophagosome assembly. *J Cell Sci* **2020,** 133, (14).
146. Xu, S.; Zhang, L.; Brodin, L., Overexpression of SNX7 reduces Abeta production by enhancing lysosomal degradation of APP. *Biochem Biophys Res Commun* **2018,** 495, (1), 12-19.
147. Luck, K.; Kim, D. K.; Lambourne, L.; Spirohn, K.; Begg, B. E.; Bian, W.; Brignall, R.; Cafarelli, T.; Campos-Laborie, F. J.; Charloteaux, B.; Choi, D.; Cote, A. G.; Daley, M.; Deimling, S.; Desbuleux, A.; Dricot, A.; Gebbia, M.; Hardy, M. F.; Kishore, N.; Knapp, J. J.; Kovacs, I. A.; Lemmens, I.; Mee, M. W.; Mellor, J. C.; Pollis, C.; Pons, C.; Richardson, A. D.; Schlabach, S.; Teeking, B.; Yadav, A.; Babor, M.; Balcha, D.; Basha, O.; Bowman-Colin, C.; Chin, S. F.; Choi, S. G.; Colabella, C.; Coppin, G.; D'Amata, C.; De Ridder, D.; De Rouck, S.; Duran-Frigola, M.; Ennajdaoui, H.; Goebels, F.; Goehring, L.; Gopal, A.; Haddad, G.; Hatchi, E.; Helmy, M.; Jacob, Y.; Kassa, Y.; Landini, S.; Li, R.; van Lieshout, N.; MacWilliams, A.; Markey, D.; Paulson, J. N.; Rangarajan, S.; Rasla, J.; Rayhan, A.; Rolland, T.; San-Miguel, A.; Shen, Y.; Sheykhkarimli, D.; Sheynkman, G. M.; Simonovsky, E.; Tasan, M.; Tejeda, A.; Tropepe, V.; Twizere, J. C.; Wang, Y.; Weatheritt, R. J.; Weile, J.; Xia, Y.; Yang, X.; Yeger-Lotem, E.; Zhong, Q.; Aloy, P.; Bader, G. D.; De Las Rivas, J.; Gaudet, S.; Hao, T.; Rak, J.; Tavernier, J.; Hill, D. E.; Vidal, M.; Roth, F. P.; Calderwood, M. A., A reference map of the human binary protein interactome. *Nature* **2020,** 580, (7803), 402-408.

148. Lin, Y. H.; Lin, Y. M.; Kuo, Y. C.; Wang, Y. Y.; Kuo, P. L., Identification and characterization of a novel Rab GTPase-activating protein in spermatids. *Int J Androl* **2011,** 34, (5 Pt 2), e358-67.
149. Muhammad, A.; Flores, I.; Zhang, H.; Yu, R.; Staniszewski, A.; Planel, E.; Herman, M.; Ho, L.; Kreber, R.; Honig, L. S.; Ganetzky, B.; Duff, K.; Arancio, O.; Small, S. A., Retromer deficiency observed in Alzheimer's disease causes hippocampal dysfunction, neurodegeneration, and Abeta accumulation. *Proc Natl Acad Sci U S A* **2008,** 105, (20), 7327-32.
150. Curtis, M. E.; Yu, D.; Pratico, D., Dysregulation of the Retromer Complex System in Down Syndrome. *Ann Neurol* **2020,** 88, (1), 137-147.
151. Chung, C. W.; Zhang, Q. L.; Qiao, L. Y., Endogenous nerve growth factor regulates collagen expression and bladder hypertrophy through Akt and MAPK pathways during cystitis. *J Biol Chem* **2010,** 285, (6), 4206-4212.
152. Kushida, N.; Yamaguchi, O.; Kawashima, Y.; Akaihata, H.; Hata, J.; Ishibashi, K.; Aikawa, K.; Kojima, Y., Uni-axial stretch induces actin stress fiber reorganization and activates c-Jun NH2 terminal kinase via RhoA and Rho kinase in human bladder smooth muscle cells. *BMC Urol* **2016,** 16, 9.
153. Scheinfeld, M. H.; Ghersi, E.; Davies, P.; D'Adamio, L., Amyloid beta protein precursor is phosphorylated by JNK-1 independent of, yet facilitated by, JNK-interacting protein (JIP)-1. *J Biol Chem* **2003,** 278, (43), 42058-63.
154. Taru, H.; Suzuki, T., Facilitation of stress-induced phosphorylation of beta-amyloid precursor protein family members by X11-like/Mint2 protein. *J Biol Chem* **2004,** 279, (20), 21628-36.
155. Watanabe, T.; Hikichi, Y.; Willuweit, A.; Shintani, Y.; Horiguchi, T., FBL2 regulates amyloid precursor protein (APP) metabolism by promoting ubiquitination-dependent APP degradation and inhibition of APP endocytosis. *J Neurosci* **2012,** 32, (10), 3352-65.
156. Watanabe, T.; von der Kammer, H.; Wang, X.; Shintani, Y.; Horiguchi, T., Neuronal expression of F-box and leucine-rich-repeat protein 2 decreases over Braak stages in the brains of Alzheimer's disease patients. *Neurodegener Dis* **2013,** 11, (1), 1-12.
157. Hur, J. Y.; Teranishi, Y.; Kihara, T.; Yamamoto, N. G.; Inoue, M.; Hosia, W.; Hashimoto, M.; Winblad, B.; Frykman, S.; Tjernberg, L. O., Identification of novel gamma-secretase-associated proteins in detergent-resistant membranes from brain. *J Biol Chem* **2012,** 287, (15), 11991-2005.
158. Del Prete, D.; Rice, R. C.; Rajadhyaksha, A. M.; D'Adamio, L., Amyloid Precursor Protein (APP) May Act as a Substrate and a Recognition Unit for CRL4CRBN and Stub1 E3 Ligases Facilitating Ubiquitination of Proteins Involved in Presynaptic Functions and Neurodegeneration. *J Biol Chem* **2016,** 291, (33), 17209-27.
159. Young-Pearse, T. L.; Chen, A. C.; Chang, R.; Marquez, C.; Selkoe, D. J., Secreted APP regulates the function of full-length APP in neurite outgrowth through interaction with integrin beta1. *Neural Dev* **2008,** 3, 15.
160. Beher, D.; Hesse, L.; Masters, C. L.; Multhaup, G., Regulation of amyloid protein precursor (APP) binding to collagen and mapping of the binding sites on APP and collagen type I. *J Biol Chem* **1996,** 271, (3), 1613-20.
161. Georgopoulos, N. T.; Steele, L. P.; Thomson, M. J.; Selby, P. J.; Southgate, J.; Trejdosiewicz, L. K., A novel mechanism of CD40-induced apoptosis of carcinoma cells involving TRAF3 and JNK/AP-1 activation. *Cell Death Differ* **2006,** 13, (10), 1789-801.
162. Jiang, Y.; Chen, G.; Zhang, Y.; Lu, L.; Liu, S.; Cao, X., Nerve growth factor promotes TLR4 signaling-induced maturation of human dendritic cells in vitro through inducible p75NTR 1. *J Immunol* **2007,** 179, (9), 6297-304.
163. Qin, H.; Wilson, C. A.; Roberts, K. L.; Baker, B. J.; Zhao, X.; Benveniste, E. N., IL-10 inhibits lipopolysaccharide-induced CD40 gene expression through induction of suppressor of cytokine signaling-3. *J Immunol* **2006,** 177, (11), 7761-71.
164. de Totero, D.; Meazza, R.; Capaia, M.; Fabbi, M.; Azzarone, B.; Balleari, E.; Gobbi, M.; Cutrona, G.; Ferrarini, M.; Ferrini, S., The opposite effects of IL-15 and IL-21 on CLL B cells

correlate with differential activation of the JAK/STAT and ERK1/2 pathways. *Blood* **2008,** 111, (2), 517-24.
165. Hase, H.; Kanno, Y.; Kojima, H.; Morimoto, C.; Okumura, K.; Kobata, T., CD27 and CD40 inhibit p53-independent mitochondrial pathways in apoptosis of B cells induced by B cell receptor ligation. *J Biol Chem* **2002,** 277, (49), 46950-8.
166. Sutherland, C. L.; Heath, A. W.; Pelech, S. L.; Young, P. R.; Gold, M. R., Differential activation of the ERK, JNK, and p38 mitogen-activated protein kinases by CD40 and the B cell antigen receptor. *J Immunol* **1996,** 157, (8), 3381-90.
167. Schwabe, R. F.; Schnabl, B.; Kweon, Y. O.; Brenner, D. A., CD40 activates NF-kappa B and c-Jun N-terminal kinase and enhances chemokine secretion on activated human hepatic stellate cells. *J Immunol* **2001,** 166, (11), 6812-9.
168. Ensminger, S. M.; Spriewald, B. M.; Sorensen, H. V.; Witzke, O.; Flashman, E. G.; Bushell, A.; Morris, P. J.; Rose, M. L.; Rahemtulla, A.; Wood, K. J., Critical role for IL-4 in the development of transplant arteriosclerosis in the absence of CD40-CD154 costimulation. *J Immunol* **2001,** 167, (1), 532-41.
169. Woodling, N. S.; Wang, Q.; Priyam, P. G.; Larkin, P.; Shi, J.; Johansson, J. U.; Zagol-Ikapitte, I.; Boutaud, O.; Andreasson, K. I., Suppression of Alzheimer-associated inflammation by microglial prostaglandin-E2 EP4 receptor signaling. *J Neurosci* **2014,** 34, (17), 5882-94.
170. Triaca, V.; Sposato, V.; Bolasco, G.; Ciotti, M. T.; Pelicci, P.; Bruni, A. C.; Cupidi, C.; Maletta, R.; Feligioni, M.; Nistico, R.; Canu, N.; Calissano, P., NGF controls APP cleavage by downregulating APP phosphorylation at Thr668: relevance for Alzheimer's disease. *Aging Cell* **2016,** 15, (4), 661-72.
171. Zhang, J.; Chen, X., DeltaNp73 modulates nerve growth factor-mediated neuronal differentiation through repression of TrkA. *Mol Cell Biol* **2007,** 27, (10), 3868-80.
172. Hwang, E. S.; White, I. A.; Ho, I. C., An IL-4-independent and CD25-mediated function of c-maf in promoting the production of Th2 cytokines. *Proc Natl Acad Sci U S A* **2002,** 99, (20), 13026-30.
173. Williams, M. E.; Feldman, D. H.; McCue, A. F.; Brenner, R.; Velicelebi, G.; Ellis, S. B.; Harpold, M. M., Structure and functional expression of alpha 1, alpha 2, and beta subunits of a novel human neuronal calcium channel subtype. *Neuron* **1992,** 8, (1), 71-84.
174. Brust, P. F.; Simerson, S.; McCue, A. F.; Deal, C. R.; Schoonmaker, S.; Williams, M. E.; Velicelebi, G.; Johnson, E. C.; Harpold, M. M.; Ellis, S. B., Human neuronal voltage-dependent calcium channels: studies on subunit structure and role in channel assembly. *Neuropharmacology* **1993,** 32, (11), 1089-102.
175. Olsen, S. M.; Stover, J. D.; Nagatomi, J., Examining the role of mechanosensitive ion channels in pressure mechanotransduction in rat bladder urothelial cells. *Ann Biomed Eng* **2011,** 39, (2), 688-97.
176. Polster, A.; Perni, S.; Bichraoui, H.; Beam, K. G., Stac adaptor proteins regulate trafficking and function of muscle and neuronal L-type Ca2+ channels. *Proc Natl Acad Sci U S A* **2015,** 112, (2), 602-6.
177. Campiglio, M.; Coste de Bagneaux, P.; Ortner, N. J.; Tuluc, P.; Van Petegem, F.; Flucher, B. E., STAC proteins associate to the IQ domain of CaV1.2 and inhibit calcium-dependent inactivation. *Proc Natl Acad Sci U S A* **2018,** 115, (6), 1376-1381.
178. Darblade, B.; Behr-Roussel, D.; Oger, S.; Hieble, J. P.; Lebret, T.; Gorny, D.; Benoit, G.; Alexandre, L.; Giuliano, F., Effects of potassium channel modulators on human detrusor smooth muscle myogenic phasic contractile activity: potential therapeutic targets for overactive bladder. *Urology* **2006,** 68, (2), 442-8.
179. Chen, H.; Vandorpe, D. H.; Xie, X.; Alper, S. L.; Zeidel, M. L.; Yu, W., Disruption of Cav1.2-mediated signaling is a pathway for ketamine-induced pathology. *Nat Commun* **2020,** 11, (1), 4328.
180. Dreses-Werringloer, U.; Lambert, J. C.; Vingtdeux, V.; Zhao, H.; Vais, H.; Siebert, A.; Jain, A.; Koppel, J.; Rovelet-Lecrux, A.; Hannequin, D.; Pasquier, F.; Galimberti, D.; Scarpini, E.; Mann,

D.; Lendon, C.; Campion, D.; Amouyel, P.; Davies, P.; Foskett, J. K.; Campagne, F.; Marambaud, P., A polymorphism in CALHM1 influences Ca2+ homeostasis, Abeta levels, and Alzheimer's disease risk. *Cell* **2008,** 133, (7), 1149-61.
181. Lei, F.; Song, J.; Haque, R.; Xiong, X.; Fang, D.; Wu, Y.; Lens, S. M.; Croft, M.; Song, J., Transgenic expression of survivin compensates for OX40-deficiency in driving Th2 development and allergic inflammation. *Eur J Immunol* **2013,** 43, (7), 1914-24.
182. Lambracht-Washington, D.; Rosenberg, R. N., Co-stimulation with TNF receptor superfamily 4/25 antibodies enhances in-vivo expansion of CD4+CD25+Foxp3+ T cells (Tregs) in a mouse study for active DNA Abeta42 immunotherapy. *J Neuroimmunol* **2015,** 278, 90-9.
183. Wang, H. C.; Klein, J. R., Multiple levels of activation of murine CD8(+) intraepithelial lymphocytes defined by OX40 (CD134) expression: effects on cell-mediated cytotoxicity, IFN-gamma, and IL-10 regulation. *J Immunol* **2001,** 167, (12), 6717-23.
184. Paris, D.; Ait-Ghezala, G.; Mathura, V. S.; Patel, N.; Quadros, A.; Laporte, V.; Mullan, M., Anti-angiogenic activity of the mutant Dutch A(beta) peptide on human brain microvascular endothelial cells. *Brain Res Mol Brain Res* **2005,** 136, (1-2), 212-30.
185. Sebollela, A.; Freitas-Correa, L.; Oliveira, F. F.; Paula-Lima, A. C.; Saraiva, L. M.; Martins, S. M.; Mota, L. D.; Torres, C.; Alves-Leon, S.; de Souza, J. M.; Carraro, D. M.; Brentani, H.; De Felice, F. G.; Ferreira, S. T., Amyloid-beta oligomers induce differential gene expression in adult human brain slices. *J Biol Chem* **2012,** 287, (10), 7436-45.
186. Zoudilova, M.; Kumar, P.; Ge, L.; Wang, P.; Bokoch, G. M.; DeFea, K. A., Beta-arrestin-dependent regulation of the cofilin pathway downstream of protease-activated receptor-2. *J Biol Chem* **2007,** 282, (28), 20634-46.
187. Wrobel, A., Overactive bladder syndrome pharmacotherapy: future treatment options. *Prz Menopauzalny* **2015,** 14, (4), 211-7.
188. Ma, E.; Vetter, J.; Bliss, L.; Lai, H. H.; Mysorekar, I. U.; Jain, S., A multiplexed analysis approach identifies new association of inflammatory proteins in patients with overactive bladder. *Am J Physiol Renal Physiol* **2016,** 311, (1), F28-34.
189. Wilson, P. G.; Thompson, J. C.; Shridas, P.; McNamara, P. J.; de Beer, M. C.; de Beer, F. C.; Webb, N. R.; Tannock, L. R., Serum Amyloid A Is an Exchangeable Apolipoprotein. *Arterioscler Thromb Vasc Biol* **2018,** 38, (8), 1890-1900.
190. Jahangiri, A.; Wilson, P. G.; Hou, T.; Brown, A.; King, V. L.; Tannock, L. R., Serum amyloid A is found on ApoB-containing lipoproteins in obese humans with diabetes. *Obesity (Silver Spring)* **2013,** 21, (5), 993-6.
191. Pieper, R.; Gatlin, C. L.; McGrath, A. M.; Makusky, A. J.; Mondal, M.; Seonarain, M.; Field, E.; Schatz, C. R.; Estock, M. A.; Ahmed, N.; Anderson, N. G.; Steiner, S., Characterization of the human urinary proteome: a method for high-resolution display of urinary proteins on two-dimensional electrophoresis gels with a yield of nearly 1400 distinct protein spots. *Proteomics* **2004,** 4, (4), 1159-74.
192. Spahr, C. S.; Davis, M. T.; McGinley, M. D.; Robinson, J. H.; Bures, E. J.; Beierle, J.; Mort, J.; Courchesne, P. L.; Chen, K.; Wahl, R. C.; Yu, W.; Luethy, R.; Patterson, S. D., Towards defining the urinary proteome using liquid chromatography-tandem mass spectrometry. I. Profiling an unfractionated tryptic digest. *Proteomics* **2001,** 1, (1), 93-107.
193. Nelson, T. J.; Alkon, D. L., Protection against beta-amyloid-induced apoptosis by peptides interacting with beta-amyloid. *J Biol Chem* **2007,** 282, (43), 31238-49.
194. Jang, S.; Jang, W. Y.; Choi, M.; Lee, J.; Kwon, W.; Yi, J.; Park, S. J.; Yoon, D.; Lee, S.; Kim, M. O.; Ryoo, Z. Y., Serum amyloid A1 is involved in amyloid plaque aggregation and memory decline in amyloid beta abundant condition. *Transgenic Res* **2019,** 28, (5-6), 499-508.
195. Bosanquet, D. C.; Ye, L.; Harding, K. G.; Jiang, W. G., Expressed in high metastatic cells (Ehm2) is a positive regulator of keratinocyte adhesion and motility: The implication for wound healing. *J Dermatol Sci* **2013,** 71, (2), 115-21.

196. Guo, Z.; Neilson, L. J.; Zhong, H.; Murray, P. S.; Zanivan, S.; Zaidel-Bar, R., E-cadherin interactome complexity and robustness resolved by quantitative proteomics. *Sci Signal* **2014,** 7, (354), rs7.
197. Tzimas, C.; Michailidou, G.; Arsenakis, M.; Kieff, E.; Mosialos, G.; Hatzivassiliou, E. G., Human ubiquitin specific protease 31 is a deubiquitinating enzyme implicated in activation of nuclear factor-kappaB. *Cell Signal* **2006,** 18, (1), 83-92.
198. Li, S.; Wang, D.; Zhao, J.; Weathington, N. M.; Shang, D.; Zhao, Y., The deubiquitinating enzyme USP48 stabilizes TRAF2 and reduces E-cadherin-mediated adherens junctions. *FASEB J* **2018,** 32, (1), 230-242.
199. Dunford, P. J.; O'Donnell, N.; Riley, J. P.; Williams, K. N.; Karlsson, L.; Thurmond, R. L., The histamine H4 receptor mediates allergic airway inflammation by regulating the activation of CD4+ T cells. *J Immunol* **2006,** 176, (11), 7062-70.
200. Nishimura, J.; Seguchi, H.; Sakihara, C.; Kureishi, Y.; Yoshimura, H.; Kobayashi, S.; Kanaide, H., The relaxant effect of adrenomedullin on particular smooth muscles despite a general expression of its mRNA in smooth muscle, endothelial and epithelial cells. *Br J Pharmacol* **1997,** 120, (2), 193-200.
201. Herrero, C.; Hu, X.; Li, W. P.; Samuels, S.; Sharif, M. N.; Kotenko, S.; Ivashkiv, L. B., Reprogramming of IL-10 activity and signaling by IFN-gamma. *J Immunol* **2003,** 171, (10), 5034-41.
202. Kim, M. J.; Lee, W.; Park, E. J.; Park, S. Y., C1qTNF-related protein-6 increases the expression of interleukin-10 in macrophages. *Mol Cells* **2010,** 30, (1), 59-64.
203. Birder, L. A.; de Groat, W. C., Mechanisms of disease: involvement of the urothelium in bladder dysfunction. *Nat Clin Pract Urol* **2007,** 4, (1), 46-54.
204. Avanes, A.; Lenz, G.; Momand, J., Darpp-32 and t-Darpp protein products of PPP1R1B: Old dogs with new tricks. *Biochem Pharmacol* **2019,** 160, 71-79.
205. Snyder, G. L.; Fienberg, A. A.; Huganir, R. L.; Greengard, P., A dopamine/D1 receptor/protein kinase A/dopamine- and cAMP-regulated phosphoprotein (Mr 32 kDa)/protein phosphatase-1 pathway regulates dephosphorylation of the NMDA receptor. *J Neurosci* **1998,** 18, (24), 10297-303.
206. Larimore, J.; Zlatic, S. A.; Gokhale, A.; Tornieri, K.; Singleton, K. S.; Mullin, A. P.; Tang, J.; Talbot, K.; Faundez, V., Mutations in the BLOC-1 subunits dysbindin and muted generate divergent and dosage-dependent phenotypes. *J Biol Chem* **2014,** 289, (20), 14291-300.
207. Gonzalez-Cadavid, N. F.; Ryndin, I.; Vernet, D.; Magee, T. R.; Rajfer, J., Presence of NMDA receptor subunits in the male lower urogenital tract. *J Androl* **2000,** 21, (4), 566-78.
208. Liu, M.; Shen, S.; Kendig, D. M.; Mahavadi, S.; Murthy, K. S.; Grider, J. R.; Qiao, L. Y., Inhibition of NMDAR reduces bladder hypertrophy and improves bladder function in cyclophosphamide induced cystitis. *J Urol* **2015,** 193, (5), 1676-83.
209. Leung, Y. F.; Ma, P.; Dowling, J. E., Gene expression profiling of zebrafish embryonic retinal pigment epithelium in vivo. *Invest Ophthalmol Vis Sci* **2007,** 48, (2), 881-90.
210. Jurgensen, S.; Antonio, L. L.; Mussi, G. E.; Brito-Moreira, J.; Bomfim, T. R.; De Felice, F. G.; Garrido-Sanabria, E. R.; Cavalheiro, E. A.; Ferreira, S. T., Activation of D1/D5 dopamine receptors protects neurons from synapse dysfunction induced by amyloid-beta oligomers. *J Biol Chem* **2011,** 286, (5), 3270-6.
211. Hinton, A. O., Jr.; He, Y.; Xia, Y.; Xu, P.; Yang, Y.; Saito, K.; Wang, C.; Yan, X.; Shu, G.; Henderson, A.; Clegg, D. J.; Khan, S. A.; Reynolds, C.; Wu, Q.; Tong, Q.; Xu, Y., Estrogen Receptor-alpha in the Medial Amygdala Prevents Stress-Induced Elevations in Blood Pressure in Females. *Hypertension* **2016,** 67, (6), 1321-30.
212. Liu, W. T.; Han, Y.; Liu, Y. P.; Song, A. A.; Barnes, B.; Song, X. J., Spinal matrix metalloproteinase-9 contributes to physical dependence on morphine in mice. *J Neurosci* **2010,** 30, (22), 7613-23.

213. Cousins, S. L.; Hoey, S. E.; Anne Stephenson, F.; Perkinton, M. S., Amyloid precursor protein 695 associates with assembled NR2A- and NR2B-containing NMDA receptors to result in the enhancement of their cell surface delivery. *J Neurochem* **2009,** 111, (6), 1501-13.
214. Husi, H.; Ward, M. A.; Choudhary, J. S.; Blackstock, W. P.; Grant, S. G., Proteomic analysis of NMDA receptor-adhesion protein signaling complexes. *Nat Neurosci* **2000,** 3, (7), 661-9.
215. Jung, S.; Nah, J.; Han, J.; Choi, S. G.; Kim, H.; Park, J.; Pyo, H. K.; Jung, Y. K., Dual-specificity phosphatase 26 (DUSP26) stimulates Abeta42 generation by promoting amyloid precursor protein axonal transport during hypoxia. *J Neurochem* **2016,** 137, (5), 770-81.
216. Ewing, R. M.; Chu, P.; Elisma, F.; Li, H.; Taylor, P.; Climie, S.; McBroom-Cerajewski, L.; Robinson, M. D.; O'Connor, L.; Li, M.; Taylor, R.; Dharsee, M.; Ho, Y.; Heilbut, A.; Moore, L.; Zhang, S.; Ornatsky, O.; Bukhman, Y. V.; Ethier, M.; Sheng, Y.; Vasilescu, J.; Abu-Farha, M.; Lambert, J. P.; Duewel, H. S.; Stewart, II; Kuehl, B.; Hogue, K.; Colwill, K.; Gladwish, K.; Muskat, B.; Kinach, R.; Adams, S. L.; Moran, M. F.; Morin, G. B.; Topaloglou, T.; Figeys, D., Large-scale mapping of human protein-protein interactions by mass spectrometry. *Mol Syst Biol* **2007,** 3, 89.
217. Bruemmer, D.; Yin, F.; Liu, J.; Kiyono, T.; Fleck, E.; Van Herle, A.; Graf, K.; Law, R. E., Atorvastatin inhibits expression of minichromosome maintenance proteins in vascular smooth muscle cells. *Eur J Pharmacol* **2003,** 462, (1-3), 15-23.
218. Rebbeck, R. T.; Karunasekara, Y.; Gallant, E. M.; Board, P. G.; Beard, N. A.; Casarotto, M. G.; Dulhunty, A. F., The beta(1a) subunit of the skeletal DHPR binds to skeletal RyR1 and activates the channel via its 35-residue C-terminal tail. *Biophys J* **2011,** 100, (4), 922-30.
219. Tilgen, N.; Zorzato, F.; Halliger-Keller, B.; Muntoni, F.; Sewry, C.; Palmucci, L. M.; Schneider, C.; Hauser, E.; Lehmann-Horn, F.; Muller, C. R.; Treves, S., Identification of four novel mutations in the C-terminal membrane spanning domain of the ryanodine receptor 1: association with central core disease and alteration of calcium homeostasis. *Hum Mol Genet* **2001,** 10, (25), 2879-87.
220. Monnier, N.; Kozak-Ribbens, G.; Krivosic-Horber, R.; Nivoche, Y.; Qi, D.; Kraev, N.; Loke, J.; Sharma, P.; Tegazzin, V.; Figarella-Branger, D.; Romero, N.; Mezin, P.; Bendahan, D.; Payen, J. F.; Depret, T.; Maclennan, D. H.; Lunardi, J., Correlations between genotype and pharmacological, histological, functional, and clinical phenotypes in malignant hyperthermia susceptibility. *Hum Mutat* **2005,** 26, (5), 413-25.
221. Yuan, Q.; Dridi, H.; Clarke, O. B.; Reiken, S.; Melville, Z.; Wronska, A.; Kushnir, A.; Zalk, R.; Sittenfeld, L.; Marks, A. R., RyR1-related myopathy mutations in ATP and calcium binding sites impair channel regulation. *Acta Neuropathol Commun* **2021,** 9, (1), 186.
222. Oules, B.; Del Prete, D.; Greco, B.; Zhang, X.; Lauritzen, I.; Sevalle, J.; Moreno, S.; Paterlini-Brechot, P.; Trebak, M.; Checler, F.; Benfenati, F.; Chami, M., Ryanodine receptor blockade reduces amyloid-beta load and memory impairments in Tg2576 mouse model of Alzheimer disease. *J Neurosci* **2012,** 32, (34), 11820-34.
223. Kristensen, J. M.; Kristensen, M.; Juel, C., Expression of Na+/HCO3- co-transporter proteins (NBCs) in rat and human skeletal muscle. *Acta Physiol Scand* **2004,** 182, (1), 69-76.
224. Parker, M. D.; Qin, X.; Williamson, R. C.; Toye, A. M.; Boron, W. F., HCO(3)(-)-independent conductance with a mutant Na(+)/HCO(3)(-) cotransporter (SLC4A4) in a case of proximal renal tubular acidosis with hypokalaemic paralysis. *J Physiol* **2012,** 590, (8), 2009-34.
225. Tietge, U. J.; Bakillah, A.; Maugeais, C.; Tsukamoto, K.; Hussain, M.; Rader, D. J., Hepatic overexpression of microsomal triglyceride transfer protein (MTP) results in increased in vivo secretion of VLDL triglycerides and apolipoprotein B. *J Lipid Res* **1999,** 40, (11), 2134-9.
226. Dikkers, A.; Annema, W.; de Boer, J. F.; Iqbal, J.; Hussain, M. M.; Tietge, U. J., Differential impact of hepatic deficiency and total body inhibition of MTP on cholesterol metabolism and RCT in mice. *J Lipid Res* **2014,** 55, (5), 816-25.
227. Pritchard, J. K.; Stephens, M.; Donnelly, P., Inference of population structure using multilocus genotype data. *Genetics* **2000,** 155, (2), 945-59.

228. Zhan, X.; Hu, Y.; Li, B.; Abecasis, G. R.; Liu, D. J., RVTESTS: an efficient and comprehensive tool for rare variant association analysis using sequence data. *Bioinformatics* **2016,** 32, (9), 1423-6.
229. Watanabe, K.; Taskesen, E.; van Bochoven, A.; Posthuma, D., Functional mapping and annotation of genetic associations with FUMA. *Nat Commun* **2017,** 8, (1), 1826.
230. Krokstad, S.; Langhammer, A.; Hveem, K.; Holmen, T. L.; Midthjell, K.; Stene, T. R.; Bratberg, G.; Heggland, J.; Holmen, J., Cohort Profile: the HUNT Study, Norway. *Int J Epidemiol* **2013,** 42, (4), 968-77.
231. Jun, G.; Flickinger, M.; Hetrick, K. N.; Romm, J. M.; Doheny, K. F.; Abecasis, G. R.; Boehnke, M.; Kang, H. M., Detecting and estimating contamination of human DNA samples in sequencing and array-based genotype data. *Am J Hum Genet* **2012,** 91, (5), 839-48.
232. Guo, Y.; He, J.; Zhao, S.; Wu, H.; Zhong, X.; Sheng, Q.; Samuels, D. C.; Shyr, Y.; Long, J., Illumina human exome genotyping array clustering and quality control. *Nat Protoc* **2014,** 9, (11), 2643-62.
233. Consortium, E. P., An integrated encyclopedia of DNA elements in the human genome. *Nature* **2012,** 489, (7414), 57-74.
234. Li, J. Z.; Absher, D. M.; Tang, H.; Southwick, A. M.; Casto, A. M.; Ramachandran, S.; Cann, H. M.; Barsh, G. S.; Feldman, M.; Cavalli-Sforza, L. L.; Myers, R. M., Worldwide human relationships inferred from genome-wide patterns of variation. *Science* **2008,** 319, (5866), 1100-4.
235. Wang, C.; Zhan, X.; Bragg-Gresham, J.; Kang, H. M.; Stambolian, D.; Chew, E. Y.; Branham, K. E.; Heckenlively, J.; Study, F.; Fulton, R.; Wilson, R. K.; Mardis, E. R.; Lin, X.; Swaroop, A.; Zollner, S.; Abecasis, G. R., Ancestry estimation and control of population stratification for sequence-based association studies. *Nat Genet* **2014,** 46, (4), 409-15.
236. Chang, C. C.; Chow, C. C.; Tellier, L. C.; Vattikuti, S.; Purcell, S. M.; Lee, J. J., Second-generation PLINK: rising to the challenge of larger and richer datasets. *Gigascience* **2015,** 4, 7.
237. Loh, P. R.; Danecek, P.; Palamara, P. F.; Fuchsberger, C.; Y, A. R.; H, K. F.; Schoenherr, S.; Forer, L.; McCarthy, S.; Abecasis, G. R.; Durbin, R.; A, L. P., Reference-based phasing using the Haplotype Reference Consortium panel. *Nat Genet* **2016,** 48, (11), 1443-1448.
238. Das, S.; Forer, L.; Schonherr, S.; Sidore, C.; Locke, A. E.; Kwong, A.; Vrieze, S. I.; Chew, E. Y.; Levy, S.; McGue, M.; Schlessinger, D.; Stambolian, D.; Loh, P. R.; Iacono, W. G.; Swaroop, A.; Scott, L. J.; Cucca, F.; Kronenberg, F.; Boehnke, M.; Abecasis, G. R.; Fuchsberger, C., Next-generation genotype imputation service and methods. *Nat Genet* **2016,** 48, (10), 1284-1287.
239. McCarthy, S.; Das, S.; Kretzschmar, W.; Delaneau, O.; Wood, A. R.; Teumer, A.; Kang, H. M.; Fuchsberger, C.; Danecek, P.; Sharp, K.; Luo, Y.; Sidore, C.; Kwong, A.; Timpson, N.; Koskinen, S.; Vrieze, S.; Scott, L. J.; Zhang, H.; Mahajan, A.; Veldink, J.; Peters, U.; Pato, C.; van Duijn, C. M.; Gillies, C. E.; Gandin, I.; Mezzavilla, M.; Gilly, A.; Cocca, M.; Traglia, M.; Angius, A.; Barrett, J. C.; Boomsma, D.; Branham, K.; Breen, G.; Brummett, C. M.; Busonero, F.; Campbell, H.; Chan, A.; Chen, S.; Chew, E.; Collins, F. S.; Corbin, L. J.; Smith, G. D.; Dedoussis, G.; Dorr, M.; Farmaki, A. E.; Ferrucci, L.; Forer, L.; Fraser, R. M.; Gabriel, S.; Levy, S.; Groop, L.; Harrison, T.; Hattersley, A.; Holmen, O. L.; Hveem, K.; Kretzler, M.; Lee, J. C.; McGue, M.; Meitinger, T.; Melzer, D.; Min, J. L.; Mohlke, K. L.; Vincent, J. B.; Nauck, M.; Nickerson, D.; Palotie, A.; Pato, M.; Pirastu, N.; McInnis, M.; Richards, J. B.; Sala, C.; Salomaa, V.; Schlessinger, D.; Schoenherr, S.; Slagboom, P. E.; Small, K.; Spector, T.; Stambolian, D.; Tuke, M.; Tuomilehto, J.; Van den Berg, L. H.; Van Rheenen, W.; Volker, U.; Wijmenga, C.; Toniolo, D.; Zeggini, E.; Gasparini, P.; Sampson, M. G.; Wilson, J. F.; Frayling, T.; de Bakker, P. I.; Swertz, M. A.; McCarroll, S.; Kooperberg, C.; Dekker, A.; Altshuler, D.; Willer, C.; Iacono, W.; Ripatti, S.; Soranzo, N.; Walter, K.; Swaroop, A.; Cucca, F.; Anderson, C. A.; Myers, R. M.; Boehnke, M.; McCarthy, M. I.; Durbin, R.; Haplotype Reference, C., A reference panel of 64,976 haplotypes for genotype imputation. *Nat Genet* **2016,** 48, (10), 1279-83.
240. Willer, C. J.; Li, Y.; Abecasis, G. R., METAL: fast and efficient meta-analysis of genomewide association scans. *Bioinformatics* **2010,** 26, (17), 2190-1.

241. Liu, J. Z.; Erlich, Y.; Pickrell, J. K., Case-control association mapping by proxy using family history of disease. *Nat Genet* **2017,** 49, (3), 325-331.
242. Euesden, J.; Lewis, C. M.; O'Reilly, P. F., PRSice: Polygenic Risk Score software. *Bioinformatics* **2015,** 31, (9), 1466-8.
243. Bralten, J.; van Hulzen, K. J.; Martens, M. B.; Galesloot, T. E.; Arias Vasquez, A.; Kiemeney, L. A.; Buitelaar, J. K.; Muntjewerff, J. W.; Franke, B.; Poelmans, G., Autism spectrum disorders and autistic traits share genetics and biology. *Mol Psychiatry* **2018,** 23, (5), 1205-1212.
244. Xicoy, H.; Klemann, C. J.; De Witte, W.; Martens, M. B.; Martens, G. J.; Poelmans, G., Shared genetic etiology between Parkinson's disease and blood levels of specific lipids. *NPJ Parkinsons Dis* **2021,** 7, (1), 23.
245. Nyholt, D. R., SECA: SNP effect concordance analysis using genome-wide association summary results. *Bioinformatics* **2014,** 30, (14), 2086-8.
